# Evaluation of noise regression techniques in resting-state fMRI studies using data of 434 older adults

**DOI:** 10.3389/fnins.2022.1006056

**Published:** 2022-10-19

**Authors:** Norman Scheel, Jeffrey N. Keller, Ellen F. Binder, Eric D. Vidoni, Jeffrey M. Burns, Binu P. Thomas, Ann M. Stowe, Linda S. Hynan, Diana R. Kerwin, Wanpen Vongpatanasin, Heidi Rossetti, C. Munro Cullum, Rong Zhang, David C. Zhu

**Affiliations:** ^1^Department of Radiology, Michigan State University, East Lansing, MI, United States; ^2^Pennington Biomedical Research Center, Baton Rouge, LA, United States; ^3^Division of Geriatrics and Nutritional Science, Washington University School of Medicine, St. Louis, MO, United States; ^4^Alzheimer’s Disease Center, University of Kansas, Fairway, KS, United States; ^5^UT Southwestern Medical Center, Dallas, TX, United States; ^6^Department of Neurology, University of Kentucky, Lexington, KY, United States; ^7^Texas Health Presbyterian Hospital, Dallas, TX, United States

**Keywords:** resting-state fMRI, aging, preprocessing, noise regression, multi-site studies

## Abstract

Subject motion is a well-known confound in resting-state functional MRI (rs-fMRI) and the analysis of functional connectivity. Consequently, several clean-up strategies have been established to minimize the impact of subject motion. Physiological signals in response to cardiac activity and respiration are also known to alter the apparent rs-fMRI connectivity. Comprehensive comparisons of common noise regression techniques showed that the “Independent Component Analysis based strategy for Automatic Removal of Motion Artifacts” (ICA-AROMA) was a preferred pre-processing technique for teenagers and adults. However, motion and physiological noise characteristics may differ substantially for older adults. Here, we present a comprehensive comparison of noise-regression techniques for older adults from a large multi-site clinical trial of exercise and intensive pharmacological vascular risk factor reduction. The Risk Reduction for Alzheimer’s Disease (rrAD) trial included hypertensive older adults (60–84 years old) at elevated risk of developing Alzheimer’s Disease (AD). We compared the performance of censoring, censoring combined with global signal regression, non-aggressive and aggressive ICA-AROMA, as well as the Spatially Organized Component Klassifikator (SOCK) on the rs-fMRI baseline scans from 434 rrAD subjects. All techniques were rated based on network reproducibility, network identifiability, edge activity, spatial smoothness, and loss of temporal degrees of freedom (tDOF). We found that non-aggressive ICA-AROMA did not perform as well as the other four techniques, which performed table with marginal differences, demonstrating the validity of these techniques. Considering reproducibility as the most important factor for longitudinal studies, given low false-positive rates and a better preserved, more cohesive temporal structure, currently aggressive ICA-AROMA is likely the most suitable noise regression technique for rs-fMRI studies of older adults.

## Introduction

Ever since the observation of low-frequency fluctuations (<0.1 Hz) and corresponding connectivity patterns in functional MRI (fMRI) recordings of resting subjects (Biswal et al., 1995), the investigation of resting-state fMRI has become a staple of modern neuroscientific research. During the last 25 years new analysis methods have been developed, yet from early on subject motion during scanning, as well as physiologic and system-related confounding factors, have been and still remain challenges for fMRI data analysis and interpretation (Jenkinson et al., 2002; Chang and Glover, 2010; Pruim et al., 2015a; Caballero-Gaudes and Reynolds, 2016).

Issues of subject motion in fMRI, as well as the impact of motion on functional connectivity, motion compensation, and motion cleaning strategies, are well established (Cole et al., 2010; Power et al., 2012; van Dijk et al., 2012; Murphy et al., 2013). Commonly, 3-dimensional fMRI volumes in a 4-dimensional timeline are linearly registered to each other (volume by volume), giving an estimation of and correction for the six motion parameters (three translational and three rotational directions). Typically, these six motion parameters are used within a General Linear Model (GLM) to regress motion-related signals that might still be present in the data after the initial alignment process. Early on, Friston et al. (1996) introduced motion derivatives and temporal shifts to better model subject motion. While this method can address gradual motion over time, it is not effective for correction of short sudden bursts of motion, thus motion spike regression, censoring, or “scrubbing” (i.e., regressing or cutting time points with high FWD rates) were designed to remedy sudden motion effects (Power et al., 2012). While these techniques can remedy motion effects, they come at the cost of altering the temporal cohesiveness and must be deployed carefully and with consideration of the subsequent analysis methods (Power et al., 2012; Pruim et al., 2015a; Scheel et al., 2018). As these procedures are widely used, residuals of subject motion-related signals are often still present after pre-processing and thus impact the results of higher-level analyses (Scheel et al., 2015). These impacts might manifest as subtle artifacts but can also be severe, like false long-range fronto-posterior connections, making the independence-verification of motion parameters from target variables indispensable (Power et al., 2012; Scheel et al., 2015).

In addition to motion, artifacts or confounds stemming from physiological fluctuations need to be addressed during pre-processing. Low-frequency fluctuations in both respiratory volume and cardiac rate can affect blood-oxygen-level-dependent (BOLD) signal, which then can obscure detection of neural activations. Regression of concurrently recorded signals of breathing and heart rate (RETROICOR-RVHR) can adequately address these issues assuming there is no temporal overlap of the intrinsic neuronal signals with these physiological variations (Chang et al., 2009; Glover et al., 2000; Scheel et al., 2014). In addition, not all scanners or sites are equipped with physiological monitoring devices. Another common way physiological artifacts are addressed is the regression of signals from the white matter (WM), cerebrospinal fluid (CSF), and the much-debated global signal (Zhu et al., 2015; Kassinopoulos and Mitsis, 2019). Average signals from regions of the WM and CSF are unlikely to contain neuro-vascular activity and are therefore commonly interpreted as broad average estimators of non-neural physiological fluctuations. The global signal, meaning the average signal of all brain voxels, has been shown to be dominated by signals stemming from upper-stream physiological fluctuations such as blood flow oscillations impacted by heart rate and respiration (Zhu et al., 2015; Kassinopoulos and Mitsis, 2019).

More recently, data-driven techniques such as the Independent Component Analysis (ICA) based “FMRIB’s ICA X-noiseifier” (ICA-FIX), the ICA based strategy for Automatic Removal of Motion Artifacts (ICA-AROMA), and the Spatially Organized Component Klassifikator (SOCK) have been introduced to improve motion and general artifact estimation and regression (Bhaganagarapu et al., 2013; Salimi-Khorshidi et al., 2014; Pruim et al., 2015b). All these procedures apply spatial Independent Component Analysis (sICA) to extract noise components from fMRI data (Beckmann and Smith, 2004). Spatial ICA components are classified as either non-noise or noise components and the corresponding temporal noise signals are used as regressors. The main differences between these techniques are how and which signal components are classified as noise. ICA-FIX uses an elaborate combination of stacked classifiers (multiple Support Vector Machines, k-Nearest Neighbors, and Trees) with a multitude of extracted features. Additionally, ICA-FIX needs to be manually trained on a subset of subjects. In the case of multi-site or multi-scanner studies, manual training is also needed on a subset of data from each site or scanner due to unique site or scanner characteristics and thus can potentially lead to dataset biases as well as high workloads (Pruim et al., 2015a). Since ICA-FIX is not well suited for multi-site studies, it was not selected for our comparison study. ICA-AROMA employs a more straightforward Linear Discriminant Analysis (LDA) approach with a basic set of signal features and operates without the need to retrain the classifier, as it is provided pretrained and automatically adapts to all rs-fMRI recordings. SOCK (Bhaganagarapu et al., 2013) was not initially developed for the purpose of noise regression. In classical ICA analysis, signal components are classified as noise or neural components so that subsequent analyses can be targeted at specific neural networks. SOCK aimed to facilitate this *post hoc* rating process by creating a classifier that mimics an expert’s decision tree when rating spatial independent components. Nonetheless, SOCK implements a noise component classification that can also be used in the context of noise identification and regression. Most importantly, ICA-AROMA and SOCK contain pre-trained classifiers and thus do not require re-training on new data, making them suitable for studies that aggregate data from multiple scanners and study sites.

The noise regression techniques discussed above are diverse and their use is much debated in the neuroimaging field (Murphy et al., 2009, 2013; Cole et al., 2010; Wu et al., 2011; Mowinckel et al., 2012; Power et al., 2012; van Dijk et al., 2012; Griffanti et al., 2014; Caballero-Gaudes and Reynolds, 2016; Liu, 2016; Satterthwaite et al., 2019). A comprehensive comparison was first conducted by Pruim et al. (2015a), on a healthy young cohort with a mean age of 15.4 ± 3.7 years. The authors compared the regression of 0, 6, and 24 motion parameters, spike regression, scrubbing, aCompCor, SOCK, ICA-FIX, and ICA-AROMA. Based on the criteria of motion artifact removal quality, preservation of signal of interest, reproducibility, identifiability, and loss of tDOF, they concluded that ICA-AROMA (non-aggressive) was the most suitable method for noise reduction. Censoring or scrubbing techniques performed reasonably well but alter the temporal autocorrelation structure. ICA-FIX showed the best reproducibility at the cost of a profound impact on neuro-vascular activity. SOCK and aCompCor had the lowest efficacy in reducing motion-related noise signals. A second comprehensive comparison was conducted using data from 34 obsessive-compulsive disorder patients, 50 schizophrenic patients, as well as 381 healthy adult controls from four independent data sets (Parkes et al., 2018). Nineteen different denoising pipelines, combining different techniques, were compared using a range of quality control metrics. The comparisons showed that the censoring-based pipelines performed best, but also had the highest degrees of data loss, making (non-aggressive) ICA-AROMA the more favorable option.

Risk Reduction for Alzheimer’s Disease (rrAD) is a multi-center clinical trial (NCT02913664) designed to assess the effects of aerobic exercise and pharmacological interventions on cognitive function in older hypertensive adults (60–85) who are at a higher risk of developing Alzheimer’s Disease and related dementias (ADRD) (Szabo-Reed et al., 2019). As part of the neuroimaging protocol, resting-state fMRI (rs-fMRI) was obtained at baseline and again after 2 years of intervention. Compared to the adolescent cohort analyzed by Pruim et al. (2015b) and the adult cohorts in Parkes et al. (2018), rrAD subjects are elderly, hypertensive, and have a high risk of developing ADRD. Subjects from the rrAD cohort are also likely to have comorbidities (Vidoni et al., 2020). Thus, the patterns of neuronal activity, motion, physiological noise, and vascular contributions to fMRI signals are expected to be significantly different from the prior studies (Mowinckel et al., 2012; Ferreira and Busatto, 2013; Ferreira et al., 2016; Pardoe et al., 2016; Biehl et al., 2017; Geerligs et al., 2017; Saccà et al., 2021).

**FIGURE 1:**
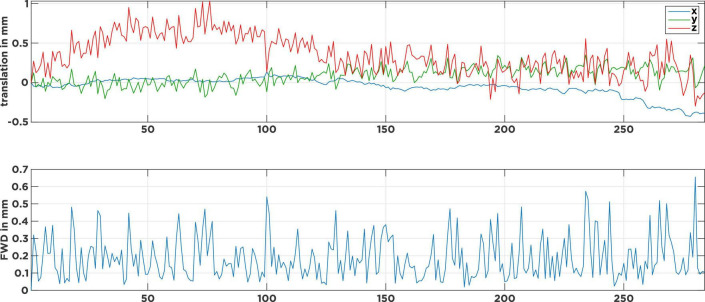
Motion parameters of a typical subject with continuous tremor-like high-frequency motion. The top panel describes the head movement induced translations in 3-dimensional space over time. The bottom panel shows the corresponding frame-wise displacements (FWD) as an estimate of motion magnitude from one volume to the next. Visible are the many FWD spikes that surpass the 0.2 mm threshold and only a few at the 0.5 mm threshold.

Figure 1 shows the head-motion plot for a typical rrAD subject. The top panel shows the translational motion in the three spatial directions over time, while the lower panel shows the framewise displacement (FWD) as an estimate of displacement magnitude from one volume to the next. Visible are continuous high-frequency (HF) motion patterns that fluctuate in each direction, with the FWDs constantly surpassing the typical 0.2-mm threshold (Power et al., 2012; Pruim et al., 2015a). Caused by heavy breathing or tremors, this type of motion pattern is typical for rrAD subjects and differs significantly from healthy young and middle-age adult subjects, whose motion plots are often much smoother and do not exhibit HF motion (Gratton et al., 2020). If the subject motion is not properly addressed in the noise regression stage of preprocessing, spurious motion artifact signals will dominate and drive temporal correlations of the fMRI signal, rather than the underlying neuronal activity. In this work we employed the baseline scans (before intervention) of 434 rrAD subjects, scanned on five different 3T MRI scanners from three vendors (Siemens, GE, and Philips), to compare the performance of different rs-fMRI noise regression techniques across different scanners. These techniques are: censoring of excessive motion, censoring combined with global signal regression, aggressive and non-aggressive AROMA, as well as SOCK. As censoring is a more traditional technique that is standardly implemented in AFNI’s proc.py script and has proven good performance in the prior assessments, we included it as a reference technique. With the current debate on global signal regression (Murphy et al., 2009, 2013), we decided to add a pipeline that combines censoring with global signal regression for comparison. The general applicability and benefits of ICA-AROMA have been shown in prior publications (Pruim et al., 2015a; Parkes et al., 2018), but the difference in efficacy and performance between non-aggressive and aggressive AROMA has not been evaluated systematically before, thus we included both versions in this comparison. SOCK was chosen as a third data-driven denoising approach as it provides an unbiased classifier that does not have to be re-trained. Furthermore, the classifier used in SOCK was created based on a human rater decision tree and thus represents a different approach. To assess the performance of these techniques, we compared their reproducibility, identifiability, amount of edge activity, spatial smoothness, and loss of tDOF. To reliably investigate longitudinal changes, we emphasized network reproducibility and identifiability as the most important metrics for the rrAD data analysis. An overview of our data processing can be found in Supplementary Figure 1.

## Materials and methods

### Dataset

A detailed description of the rrAD protocol, including inclusion and exclusion criteria, has been provided by Szabo-Reed et al. (2019). Overall, individuals with a history of severe neurological, psychological, cardiovascular, and other severe diseases were excluded from this study. An overview of the demographics for the rrAD subjects used in these analyses is provided in Table 1. As part of the rrAD neuroimaging protocol, 434 subjects underwent baseline resting-state fMRI acquisition (eyes focused on a cross) for 12 min on five different 3 Tesla MRI systems at four clinical sites: 49 subjects on a Philips Achieva, 61 subjects on a GE MR750W, both located at the University of Texas Southwestern Medical Center; 128 subjects on a GE MR750W at the Pennington Biomedical Research Center; 106 subjects on a Siemens Skyra at the University of Kansas; and 90 subjects on a Siemens Prisma at Washington University. All scanners were equipped with a 32-channel head coil, except the GE system at the University of Texas Southwestern Medical Center, where a 48-channel head/neck coil was used. The fMRI data were acquired with 2.5 s TR (time of repetition), 28 ms TE (time of echo), and a 64 × 64 matrix size with 3.4 mm × 3.4 mm pixels. A 3 mm slice thickness was used on all but the GE system at the University of Texas Southwestern Medical Center, on which thicker slices of 3.4 mm were used without parallel imaging to compensate for the reduced signal-to-noise ratio on the 48-channel head-neck coil.

**TABLE 1:** Participant characteristics.

	25%	Median	75%	Mean ± SD	Minimum	Maximum
Male/Female (n)			160/274			
Race (n)			360 Caucasian, 61 African American, 13 Hispanic			
Age (years)	64	68	72	69 ± 6	60	84
Body mass index (kg/m^2^)	26	30	34	30 ± 5	19	47
Education (years)	15	16	18	16 ± 2	12	20
MMSE total	28	29	30	29 ± 1	24	30
Heart rate (bpm)	59	68	75	68 ± 11	39	106
Systolic blood pressure (mmHg)	125	136	149	138 ± 16	103	188
Diastolic blood pressure (mmHg)	74	79	84	79 ± 7	56	116
Cardiovascular risk score*	9	14	24	18 ± 13	3	81

*10-year Atherosclerotic Cardiovascular Disease (ASCVD) risk based on AHA Pooled Cohort (Goff et al., 2014).

In addition to the functional images, anatomical 3D 1-mm^3^ isotropic T_1_-weighted MPRAGE images with CSF suppressed were also collected for each of the subjects using the following parameters: 176 sagittal slices, TE = 3.8–4 ms, TR of acquisition ≈ 8.6 ms, time of inversion (TI) = 830 ms, TR of inversion = 2,330 ms, flip angle = 8°, FOV (field of view) = 25.6 cm × 25.6 cm, matrix size = 256 × 256, slice thickness = 1 mm, and parallel imaging acceleration factor = 2.

### Functional MRI pre-processing and noise regression

The data of each subject was first processed with the AFNI (Cox, 1996) proc.py script (AFNI’s tool to create a complete and standardized fMRI preprocessing pipeline). This script includes the steps of outlier detection, de-spiking, correction for slice timing differences, functional image co-registration to anatomical recordings, alignment between functional volumes to correct for rigid motions, and smoothing using a 4 mm kernel. Building on these initial steps, we implemented five different regression techniques to compare their performance on motion correction and noise reduction:

1) **“censoring”:** As a reference for the comparison, we used the standard procedure implemented in AFNI’s proc.py: Noise regressors include 24 motion parameters (24 MP), sudden motion scrubbing/censoring regressors [Frame-wise Displacement (FWD) cutoff at < 0.5 mm], mean signal time courses from WM and CSF, a bandpass filter (0.009–0.08 Hz) and third order detrending. All regressors were combined for noise regression using AFNI’s “3dDeconvolve.”
2) **“censoring + GS”:** Adds the global mean signal (GS) time course of the whole brain to the regression matrix used in the “3dDeconvolve” step from procedure 1 above.
3) **“aggr. AROMA”:** Using the results from the initial steps, we transformed the motion parameters extracted by AFNI’s motion correction into an FSL-compatible format and performed *aggressive ICA-AROMA* (Pruim et al., 2015b) before applying the same bandpass filter as in techniques 1 and 2 above.
4) **“non-aggr. AROMA”:** Uses the same approach as technique 3 above but performs *non-aggressive ICA-AROMA*, in which partial regression instead of full regression was used in the internal regression step of ICA-AROMA (using “fsl-regfilt”) for the removal of noise components.
5) **“SOCK”:** As a comparable method to ICA-AROMA, *SOCK* implements a classifier to identify noise components from a resting-state fMRI ICA decomposition. Here we combined the noise components identified by SOCK with the 24 motion parameters, WM/CSF signals, bandpass, and detrend parameters used in technique 1 for the “3dDeconvolve” noise regression.

### Implementation of ICA-AROMA

The MATLAB implementation of ICA-AROMA version 0.4-beta was used to avoid implementation errors in Version 0.2-beta and earlier.^1^ To compare across techniques, spatial smoothing was handled in a two-step process. Pruim et al. (2015b) recommended at least 6 mm of spatial smoothing to achieve stable ICA results. However, for the rrAD study, a 4 mm smoothing kernel (slightly larger than the voxel size) is preferred, to resolve smaller structures such as parts of the hippocampus. To satisfy these two requirements, ICA-AROMA was first applied to extract noise components from the dataset which was spatially smoothed with a 6 mm kernel. The time courses of these noise components were then used as the regressors in the dataset that was spatially smoothed with a 4 mm kernel. Other optimizations we implemented for ICA-AROMA, were adjustments to cope with anatomical variations of older brains, as these often show significant ventricle enlargements and other major anatomical distinctions. ICA-AROMA uses an internal function “register2MNI” which, by default, linearly transforms IC maps from native space to MNI space for classification. This linear transformation works well for young healthy brains but often leads to alignment errors for brains that deviate greatly from the standard MNI152 brain, which was common in this study. To resolve this alignment issue, external linear and non-linear transformation matrices can be used in “register2mni.” Using the program “FLIRT” from FSL (Jenkinson et al., 2012), a transformation matrix was first created for the linear spatial alignment between the functional and 3D MPRAGE images. Then, using the program “FNIRT” from FSL (Jenkinson et al., 2012) a non-linear transformation matrix was created, warping the 3D MPRAGE image from native to MNI space. Using these transformation matrices as additional input was indispensable for ICA-AROMA to work reliably with non-normative brain anatomies.

### Data quality metrics

Like the approaches by Bhaganagarapu et al. (2013) and Pruim et al. (2015a), we assessed the performance of the techniques based on the following quality metrics: Resting state network (RSN) reproducibility, RSN identifiability, edge activity, spatial smoothness, loss of temporal degrees of freedom (tDOF), and true and false positive connectivities, as sub-scores of identifiability. All metrics were calculated for each subject and compared separately for each scanner and at the consortium level.

To avoid site or MRI system bias, we applied the following seed-based approach in RSN map creation: Each subject’s anatomical MPRAGE scan underwent Freesurfer (Fischl, 2012) segmentation to reliably extract seed regions for major resting-state networks. RSN maps were then calculated by using the respective seed region’s mean signal for whole-brain correlation. See Supplementary Table 1 for a list of seed regions and expected resting-state network connectivity. We used the 14 RSN atlas by Shirer et al. (2012) as a reference, to compare the seed-based correlation maps with the peak regions of established RSNs. All correlation indices were Fisher-*z* transformed. Metrics based on thresholded *z*-correlation maps used Gaussian mixture modeling for thresholding.

***RSN reproducibility*** estimates how stable RSN maps are across subjects. It is derived by using 500 random split-half permutations: for each permutation the dataset is randomly split into halves, representing 50% of all subjects. For each half of the dataset, the average Fisher-*z* transformed, unthresholded map of each seed correlation map is calculated. This procedure results in two average seed correlation maps (one per half) per seed (one of 22) and permutation (one of 500). The spatial Pearson correlation of the two corresponding averaged maps gives a distribution of 500 correlation values per seed. The correlation values of uncoressponding maps (off-diagonals in the 22 × 22 correlation matrix) serve as null distribution and are used to transform the correlation values (main diagonals) into pseudo-*z*-scores, for details see Pruim et al. (2015a).

***RSN identifiability*** estimates how reliable the 14 reference-RSNs from Shirer et al. (2012) can be identified using the 22 RSN maps generated from the aforementioned 22 seeds. Here we categorized these 22 RSN maps as either corresponding or not corresponding to the 14 reference-RSNs. The identifiability score of an RSN map compared to a reference map is defined by the ratio of the mean absolute *z*-score within the RSN map and the mean absolute *z*-score outside the map, for details see Pruim et al. (2015a). In a subsequent analysis, we further investigated sub-scores of identifiability: true positive connectivity, represented by the average connection strength within target network regions, and false positive connectivity, represented by the mean *r*-values outside the target networks but within the gray matter. Furthermore, strong correlations with regions of the WM and the CSF are also considered false positives.

***Edge activity*** is given by the percentage of absolute active voxels within an edge mask around the brain (after Gaussian mixture model thresholding). Activation along the edge of the brain is considered a marker of motion artifacts (Bhaganagarapu et al., 2013).

The***spatial smoothness*** measure identifies noise, that manifests as high spatial fragmentations which are not likely due to neuronal activity. When RSN maps are not spatially cohesive, but rather fragmented in many spots and patches, they are unlikely of neural origin. Spotty and patchy regions tend to contain relatively large amounts of signal with high spatial frequencies. Regions of RSN maps with neural origin on the contrary tend to have lower spatial frequencies and appear more cohesive and smooth (Bhaganagarapu et al., 2013). The spatial smoothness can be quantified with a discrete 3D Fourier transform. Specifically, it can be calculated based on the ratio of the mean amplitudes of the Fourier transformed signals between the low-frequency and HF regions defined by a sphere with the origin at the center of frequency space. Using the “smoothness_measure” function of SOCK (Bhaganagarapu et al., 2013), we calculated these smoothness fractions at six increasing spherical sizes. We used the mean of these six ratios as a measurement of the spatial smoothness of an RSN map.

Lastly, the loss of tDOF counts how many time-points of the fMRI time series are lost due to performing linear regressions. Each regressor for motion censoring or noise IC regressions represents a loss of one degree of temporal data freedom.

### Statistical analyses

Employing the data quality metrics, displayed as mean ± standard deviations in Tables 2, 3, of resting-state-network reproducibility, identifiability, edge activity, spatial smoothness, and the loss of tDOF on the rrAD baseline data, we assessed the performance of the different noise regression techniques using a best to worst ranking. In complementary analyses, we ranked the sub-scores of resting-state-network identifiability as true and false positive connectivity, as well as connectivity with WM and CSF. For voxel-wise analyses that made use of thresholded maps, we employed Gaussian Mixture Modeling for thresholding of connectivity maps. Statistical significance was tested using non-parametric Kruskal-Wallis tests to investigate the effect of the regression techniques on each of the RSN quality metrics. Pairwise Mann-Whitney *U*-tests were used for pairwise comparisons. Multiple comparisons were corrected using the Bonferroni method.

**TABLE 2:** RSN data quality metrics based on data across all scanners collected from the rrAD project.

Techniques	Reproducibility *z*-score	Identifiability *z*-score	Edge activity %	Spatial smoothness
Censoring	5.8 ± 1.2	3.5 ± 1.1	11 ± 9	0.028 ± 0.004
Censoring + GS	5.3 ± 0.7	3.4 ± 1	8 ± 4	0.023 ± 0.004
Aggressive AROMA	**6.9 ± 1.2**	3.6 ± 1	10 ± 5	**0.031 ± 0.007**
Non-aggressive AROMA	*4.5* ± *1.4*	*1.6* ± *1.2*	*26* ± *19*	0.03 ± 0.007
SOCK	6.4 ± 1.3	**4.1 ± 1**	**6 ± 2**	*0.023* ± *0.003*

Resting state network (RSN), results are presented as mean ± std, bold entries mark the best result, and italic entries mark the worst result.

**TABLE 3:** Identifiability sub-scores of connectivity.

Techniques	True positive connectivity *r*	False positive connectivity *r*	WM connectivity *r*	CSF connectivity *r*
Censoring	0.27 ± 0.15	0.1 ± 0.06	0.02 ± 0.02	0.04 ± 0.04
Censoring + GS	*0.18* ± *0.14*	**0.01 ± 0.03**	0.01 ± 0.02	**−0.01 ± 0.02**
Aggressive AROMA	0.24 ± 0.13	0.11 ± 0.06	0.07 ± 0.05	0.06 ± 0.04
Non-aggressive AROMA	**0.4 ± 0.2**	*0.24* ± *0.13*	*0.18* ± *0.13*	*0.13* ± *0.08*
SOCK	0.21 ± 0.13	0.06 ± 0.04	**0.01 ± 0.01**	0.02 ± 0.02

Results are presented as mean ± std, bold entries mark the best result, and italic entries mark the worst result.

## Results

**FIGURE 2:**
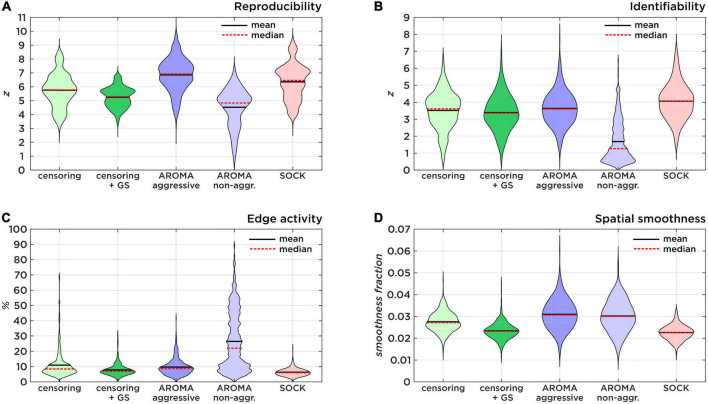
Comparison of data quality metrics. **(A)** Shows the Reproducibility as a result of 500 random split-half permutations. **(B)** Shows the Identifiability as a ratio of true-positive and false-positive connectivity compared to the target region maps. **(C)** Shows the amount of brain edge activity after Gaussian mixture modeling as an estimator of residual subject motion. **(D)** Shows smoothness fractions as a measure for spatial high-frequency noise.

**FIGURE 3:**
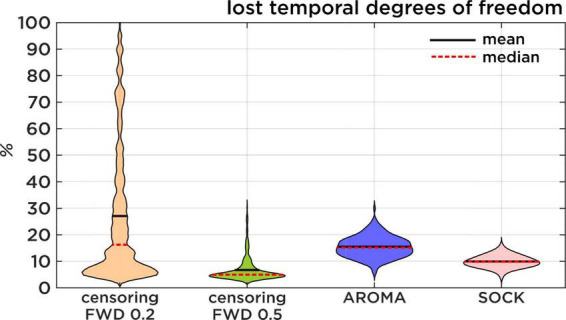
Lost temporal degrees of freedom. Comparison of the percentage of data that is lost resulting from each regression approach. Censoring results are displayed first with a theoretical FWD cut-off of 0.2 mm and then with the slightly more lenient 0.5 mm FWD cut-off. Censoring loss is induced by discarding timepoints of high motion, while AROMA and SOCK loss is induced by the number of ICA components classified as noise.

All data quality metric comparisons were carried out separately for data from each of the five MRI systems and at the consortium level, which combines the data across all systems. Tables 2, 3, as well as Figures 2, 3, summarize the findings of the performance of the noise regression techniques at the consortium level, including reproducibility, identifiability, edge activity, spatial smoothness, and loss of tDOF. Separate results for each of the MRI systems used in this study were largely comparable to the consortium results (see Supplementary material for separate listings).

The *z*-scores (mean ± std) for **RSN reproducibility** can be found in Table 2 and Figure 2A. Aggressive AROMA has the highest, while non-aggressive AROMA has the lowest reproducibility rates, with all pairwise Mann-Whitney *U*-tests and the general Kruskal-Wallis test reaching significant corrected *p*-values lower than 0.001.

The *z*-scores for **RSN identifiability** can be found in Table 2 and Figure 2B. Most techniques reached comparatively good identifiability scores, yet non-aggressive AROMA scored significantly lower while SOCK shows a slight trend of a higher score. The general Kruskal-Wallis test and most pairwise Mann-Whitney *U*-tests reached significant corrected *p*-values lower than 0.001, except for censoring compared to censoring with global signal regression, which reached a corrected *p* < 0.05 while censoring compared to aggressive AROMA did not show a significant difference in identifiability.

Percentages of **edge activity** are shown in Table 2 and Figure 2C. Activity around the edge of the brain was highest for non-aggressive AROMA, the other techniques each show quantitatively low edge activity measures. The general Kruskal-Wallis test and most pairwise Mann-Whitney *U*-tests reached significant corrected *p*-values lower than 0.001, except censoring compared to aggressive AROMA, which did not show a significant difference in edge activity.

**Spatial smoothness** fractions can be found in Table 2 and Figure 2D. Aggressive and non-aggressive AROMA produce maps that are spatially smoother than the other techniques. The general Kruskal-Wallis test and most pairwise Mann-Whitney *U*-tests reached significant corrected *p*-values lower than 0.001, except censoring with global signal regression compared to SOCK, which reached a corrected *p* < 0.01 while aggressive AROMA compared to non-aggressive AROMA did not show a significant difference in spatial smoothness.

**Loss of temporal degree of freedom (TDF)** compares AROMA, SOCK, and censoring techniques. For AROMA and SOCK, the loss of TDF depends on the number of components classified as noise, while the loss of TDF for censoring varies depending on the FWD cut-off used. At an FWD cut-off of 0.5 mm, as used in this study, the average loss of TDF was 7 ± 4%. At an FWD cut-off of 0.2 mm [used by Pruim et al. (2015a)], the average loss of TDF would be 27 ± 25%, leading to a substantial amount of data loss for some subjects. The AROMA techniques have lost 15 ± 4% TDF, while SOCK lost 10 ± 2% TDF (Figure 3). The general Kruskal-Wallis test and most pairwise Mann-Whitney *U*-tests reached significant corrected *p*-values lower than 0.001, except for the pairwise Mann-Whitney *U*-test between censoring with an FWD cut-off of 0.2 mm and the AROMA techniques, which did not show a significant difference in lost tDOF.

**FIGURE 4:**
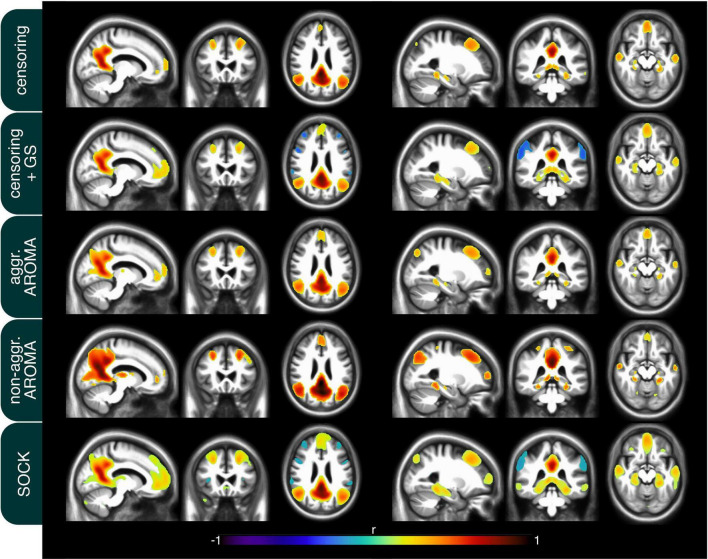
Mean default mode network (DMN) connectivity map of 434 rrAD subjects in MNI space with the results of each of the regression approaches overlayed on the subject’s mean MPRAGE images. On average, the seed-based DMN can be calculated reliably with each of the 5 regression approaches and the differences are subtle. As expected, the regression of the global signal introduces task-positive anticorrelations for the censoring + GS approach but also for SOCK. Non-aggressive AROMA displays false positive connectivity in superior motor regions, while aggressive AROMA shows the best balance of separating basal ganglia subregions of the DMN.

**FIGURE 5:**
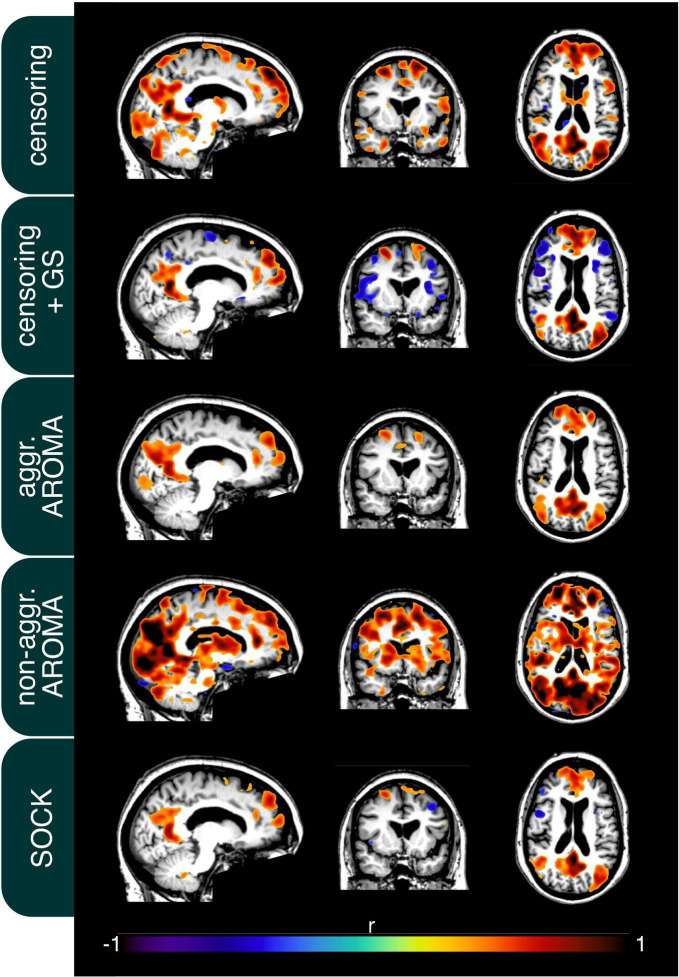
Connectivity of the default mode network (DMN) of an example subject after each regression procedure. Motion parameters for this subject can be found in Figure 1. As seen on average in Figure 4, the usage of the global signal regressor (GS) introduces task-positive anticorrelations, which can also be observed with the SOCK approach. The censoring approach without global signal regression exhibits minor motion-induced edge activity, which is more pronounced in the non-aggressive AROMA approach, where extensive amounts of false-positive connectivity can be observed across the whole brain. The aggressive AROMA approach produces the cleanest DMN map, while SOCK and censoring + GS regression approaches perform sufficiently.

In addition to the quality metrics presented above, we also computed preprocessing specific average resting-state network maps across all subjects and applied Gaussian mixture modeling for thresholding. As an example, the default mode network (DMN) is depicted in Figure 4. While all regression techniques on average produced qualitatively expected DMN maps, some differences were observed: Censoring with global signal regression and the SOCK technique revealed anticorrelated task-positive regions, which were not shown in the other techniques. Furthermore, while the non-aggressive AROMA results show the highest correlation values within the DMN, significant correlations were falsely shown in some motor regions. As rrAD subjects often exhibited HF motion patterns as seen in Figure 1, these motion patterns were manifested as variations in functional connectivity maps. The variations of the DMN maps due to the different preprocessing techniques are shown for an example subject with HF motion in Figure 5. Here, the censoring technique shows some residuals of edge activity, while censoring in combination with global signal regression, as well as SOCK, clearly show the anti-correlated task-positive regions. The non-aggressive AROMA technique shows hyper-connected regions across the whole brain, while the aggressive AROMA technique shows a quantitatively clean DMN map.

## Discussion

In this study, we qualitatively, and quantitively evaluated five commonly used preprocessing techniques for the rs-fMRI data of 434 rrAD subjects, scanned on five different scanners from three vendors. Comparing across the metrics, except for non-aggressive AROMA, the other four preprocessing techniques demonstrated to be comparable overall. Aggressive AROMA reached the highest reproducibility scores while non-aggressive AROMA scores lowest. Except for non-aggressive AROMA, the other four preprocessing techniques achieved reasonably good identifiability scores. Considering reproducibility as a crucial metric for longitudinal studies, we selected aggressive AROMA as the most suitable preprocessing technique for rrAD subjects.

**FIGURE 6:**
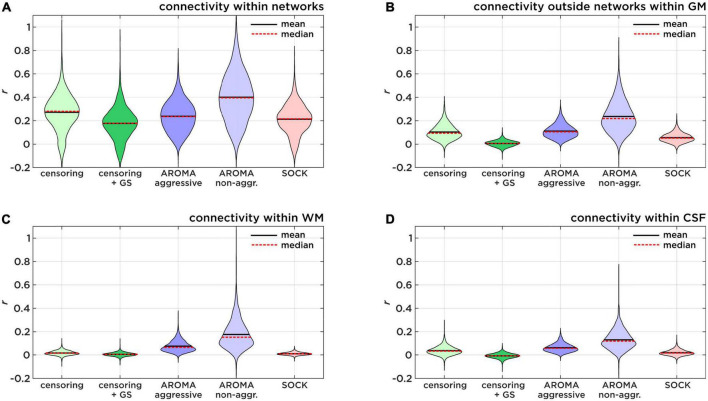
Identifiability sub-scores of connectivity. Comparison of true positive and false positive connectivity of each regression approach, investigating the low identifiability score of non-aggressive AROMA. **(A)** Shows the mean connectivity within target network regions (true-positive). **(B)** Shows the mean connectivity within the gray matter (GM) but outside of the target network regions (false-positive). **(C)** Shows the mean connectivity within the white matter (WM) and **(D)** shows the mean connectivity within regions of the cerebral spinal fluid (CSF) as measures of physiological noise-induced connectivity.

Our results aligned to a large extent with the findings of Pruim et al. (2015a) and Parkes et al. (2018), who concluded that AROMA is the recommended option for rs-fMRI noise regression. Yet, the approaches employed for data analysis leading to this conclusion may differ, as the comparisons by Pruim et al. (2015b) do not specifically state which regression model was used (i.e., non-aggressive vs. aggressive). As non-aggressive AROMA is the standard procedure (Pruim et al., 2015b), we were in fact concerned about the poor performance of non-aggressive AROMA regarding network identifiability, leading us to further investigate this issue. Figure 6 and Table 3 give an overview of the mean connectivity (mean Fisher-*z* transformed *r*-values) of different target regions of the un-thresholded connectivity maps. Figure 6A shows the connectivity within target network regions, in other words, true positive correlations. Non-aggressive AROMA significantly scores the highest true positive correlations. False-positive connectivity is shown in Figure 6B, where mean *r*-values outside the target networks but within gray matter are shown for each regression technique. For false positive connectivity, non-aggressive AROMA also scores highest. Furthermore, high connection strengths with regions of the WM and the CSF are also considered false positives, depicted in Figures 6C,D. As identifiability is defined by the ratio between true and false positive connectivity, Figure 6 and Table 3 clearly show that non-aggressive AROMA scores the highest on all categories of false positives, and consequently achieves low identifiability scores. As discussed earlier, high false-positive rates and correlations across all parts of the brain suggest a poor model fit for subject motion and other noise regressors. In other words, the residual HF motion artifact signal dominates the underlying neuronal activity. Yet, interestingly, the aggressive AROMA approach uses the same model as the non-aggressive approach. The main difference between aggressive and non-aggressive AROMA is the regression method e.g., full vs. partial regression. Our results suggest that the model of the AROMA regressors fits the data very well but only the full regression can successfully diminish noise signals stemming from HF motion and physiological variability of the rrAD cohort. This can also be observed in the results from the edge activity analysis, where scoring a low percentage is a sign of a good regression model for subject motion. Non-aggressive AROMA performed significantly worse while all other approaches seem to adequately model subject motion.

The analysis of spatial smoothness conveys how connectivity is spread across the cortex with the assumption that very fragmented maps resemble noise in comparison to spatially smooth maps, which are more likely of neural origin. Quantitatively the aggressive and non-aggressive AROMA techniques produce smoother maps than the other three techniques. Interestingly, both perform on a comparable level for this metric, speaking to better temporal cohesiveness as opposed to the censoring approaches. As expected, comparing the censoring methods, GS regression makes the resulting connectivity maps less smooth, likely due to the resulting negative correlations. The resulting negative correlations from the SOCK technique also led to less smooth connectivity maps.

Regarding the loss of tDOF, in previous studies (Pruim et al., 2015a; Parkes et al., 2018; Gratton et al., 2020) censoring motion regressors have been applied with FWD cut-off at 0.2 mm. This FWD criterion was too strict for our subject population, especially in the circumstance of HF motion, thus we opted for a slightly more lenient cutoff at 0.5 mm (Power et al., 2012). Statistical testing did not show a significant difference between AROMA and the 0.2 mm FWD censoring. Nonetheless, Figure 3 shows that the lower threshold of 0.2 mm would have led to a substantial amount of data loss for some subjects. Additionally, the slightly higher cut-off at 0.5 mm proved to be very efficient, as this approach leads to the lowest percentage of lost degrees of freedom while still reaching low edge activity ratings.

Figures 4, 5 display the findings according to the different metrics on a brain network level. Generally, all regression approaches produced reasonable RSN maps and, as expected, global signal regression gave rise to task-positive anti-correlations. Visible are the issues of false-positive correlations for the non-aggressive AROMA technique, especially on the single-subject level.

This multi-site study had several limitations. Not all scanners were set up to collect cardiac and respiratory physiological signals, thus external physiological signals were not used as regressors in our analyses (Shmueli et al., 2007; Birn, 2012; Chang et al., 2013; Scheel et al., 2014; Tsvetanov et al., 2020). Subjects from the rrAD study were recruited from clinics and the community-at-large, and represent a subset of older adults with hypertension and increased risk for AD. Although they had co-morbidities common in older adults, they were highly motivated to participate in a 2-year clinical trial of exercise and do not represent the general population of older adults or those with significant cognitive impairment or dementia. Additionally, the rrAD sample had an underrepresentation of minorities and an overrepresentation of female participants. With the specific demographic of the study population, the provided comparison is specific to older adults with hypertension and might not generalize to other pathophysiologies in different cohorts. Additionally, in this work, we did not conduct a comparison with ICA-FIX, as we were concerned about the appropriate sample sizes to train the classifier used in ICA-FIX for multi-site studies. Nevertheless, a future comparison using ICA-FIX with specific re-training suited for a multi-site configuration might be insightful. Despite these limitations, the rrAD study provides many important insights for imaging studies of older adults.

## Conclusion

Across all data quality metrics, non-aggressive AROMA was the least suitable preprocessing option for rrAD subjects. The other four techniques (censoring, censoring with global signal regression, aggressive AROMA, and SOCK) performed adequately with marginal differences, demonstrating the utility of these techniques. Aggressive AROMA achieved low false positivity rates and preserved a more cohesive temporal structure than censoring approaches, making it the most favorable technique for rs-fMRI noise regression. Considering reproducibility as the most important factor for multi-site longitudinal studies, currently, aggressive AROMA appears to be the most suitable noise regression technique for older subjects in the rrAD trial, and likely for longitudinal studies of older adults in general. Nonetheless, residuals and aliases of noise signals remain to varying degrees, posing risks for higher-level analysis, especially for highly sensitive machine learning approaches. More research needs to be done on the modeling of physiological signals when no concurrent recordings are available, as well as on the reproducibility and stability of longitudinal functional imaging results. Finally, methods for noise compensation and data preprocessing of fMRI data are constantly evolving and in development, making future comparisons imperative.

## Data availability statement

The raw data supporting the conclusions of this article will be made available by the authors, without undue reservation.

## Ethics statement

The studies involving human participants were reviewed and approved by the Michigan State University, University of Texas, University of Kansas, Washington University, Pennington Biomedical Research Center IRBs, all research sites received approval from the corresponding local IRB. The patients/participants provided their written informed consent to participate in this study.

## Author contributions

NS developed concepts, performed data analysis, and drafted and finalized the manuscript. JK, EB, EV, JB, BT, AS, DK, WV, and RZ participated in data collection and manuscript writing/editing. LH provided statistical guidance and descriptive cohort statistics. CC and HR provided clinical neuropsychological input and manuscript writing/editing. DZ developed concepts, participated in data analysis, and manuscript writing/editing. All authors contributed intellectually to conceive this work, revised, and approved the final version of the manuscript.

## Footnotes

1
https://github.com/maartenmennes/ICA-AROMA
