# Supplementary material for: Evaluation of noise regression techniques in resting-state fMRI studies using data of 434 older adults
Source: Front Neurosci. 2022 Oct 19;16:1006056. doi: 10.3389/fnins.2022.1006056 (PMC9626831; doi:10.3389/fnins.2022.1006056)

Supplementary Material

Supplementary Table 1.

Freesurfer seeds and corresponding RSNs

freesurfer seed region	RSN	Shirer RSN maps
lh-isthmuscingulate	DMN	09, 10
rh-isthmuscingulate	DMN	09, 10
Left-Hippocampus	DMN	09, 10
Right-Hippocampus	DMN	09, 10
lh-lateraloccipital	visual	05, 13, 14
rh-lateraloccipital	visual	05, 13, 14
lh-amygdala	salience/emotion	11, 12
rh-amygdala	salience/emotion	11, 12
lh-transversetemporal	auditory	02
rh-transversetemporal	auditory	02
lh-front-inf-opercular	salience	11, 12
rh-front-inf-opercular	salience	11, 12
lh-front-inf-triangular	language	06
rh-front-inf-triangular	language	06
lh-PCG	primary motor	01
rh-PCG	primary motor	01
lh-parietal_sup	visuospatial	05, 13, 14
rh-parietal_sup	visuospatial	05, 13, 14
lh-cuneus	visual	05, 13, 14
rh-cuneus	visual	05, 13, 14
lh-putamen	basal ganglia	03
rh-putamen	basal ganglia	03

Shirer RSNs (Shirer et al., 2012):
01 Sensorimotor, 02 Auditory, 03 Basal Ganglia, 04 Precuneus,
05 Visuospatial, 06 Language, 07 left executive control network (LECN),
08 right executive control network (RECN), 09 dorsal default mode network (dDMN),
10 ventral default mode network (vDMN), 11 anterior Salience, 12 posterior Salience,
13 higher Visual, 14 primary Visual

Supplementary Figure 1. Overview of data processing of subjects from 5 scanners.

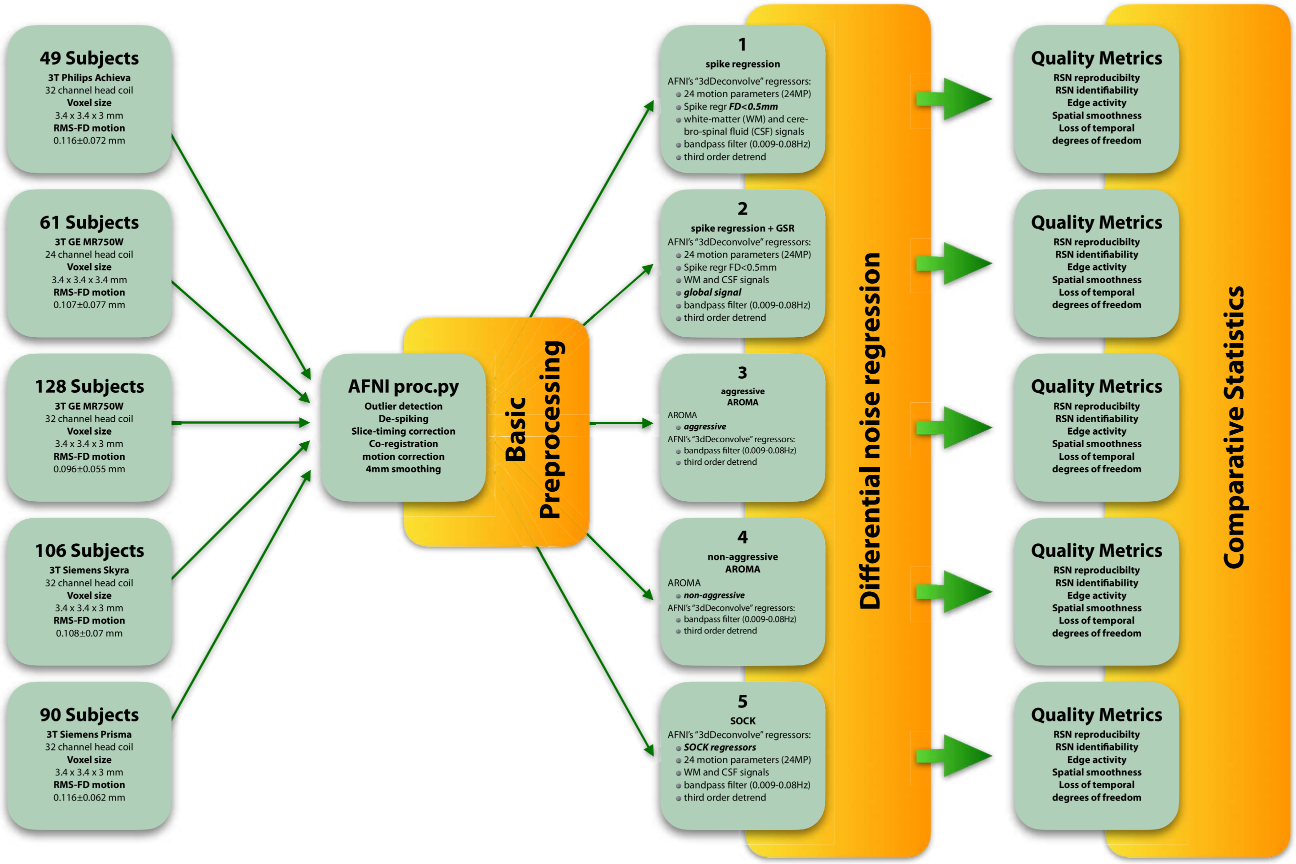

Supplementary Figure 2.

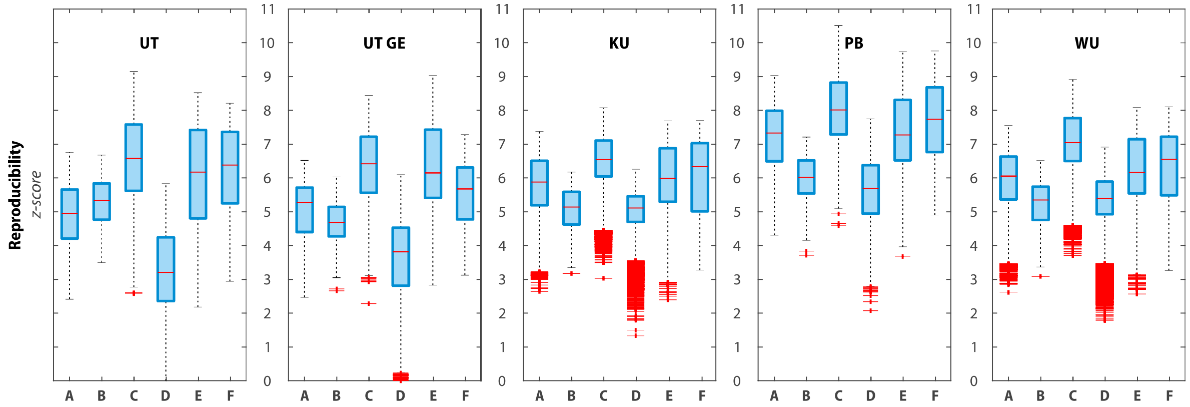

Resting-State Network Reproducibility for each scanner:
UT = University of Texas Philips, UT GE = University of Texas GE, KU = Kansas University Siemens, PB = Pennington Biomedical Research Center GE, WU = Washington University Siemens, with following preprocessing methods: A – censoring, B – censoring with global signal regression, C – aggressive AROMA, D – non-aggressive AROMA,
E – one-step AROMA (deprecated), F – SOCK

Supplementary Figure 3.

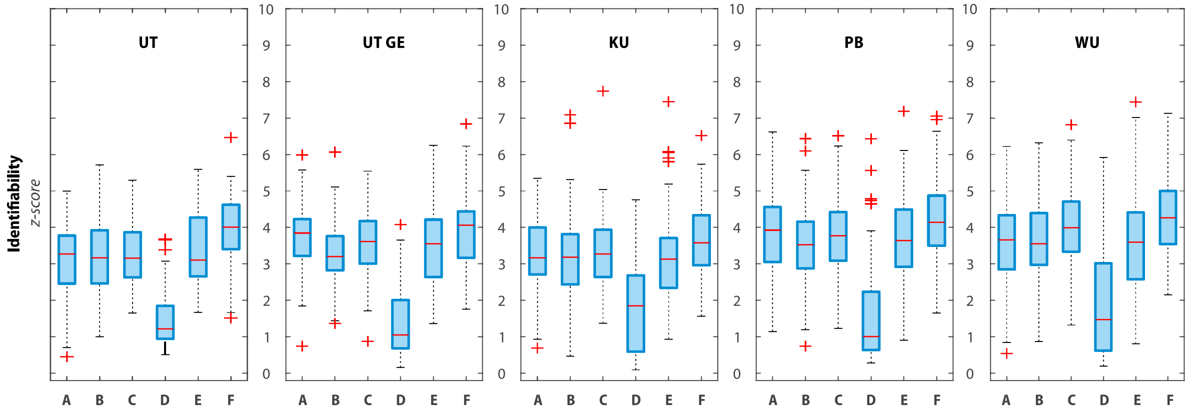

Resting-State Network Identifiability for each scanner:
UT = University of Texas Philips, UT GE = University of Texas GE, KU = Kansas University Siemens, PB = Pennington Biomedical Research Center GE, WU = Washington University Siemens, with following preprocessing methods: A – censoring, B – censoring with global signal regression, C – aggressive AROMA, D – non-aggressive AROMA,
E – one-step AROMA (deprecated), F – SOCK

Supplementary Figure 4.

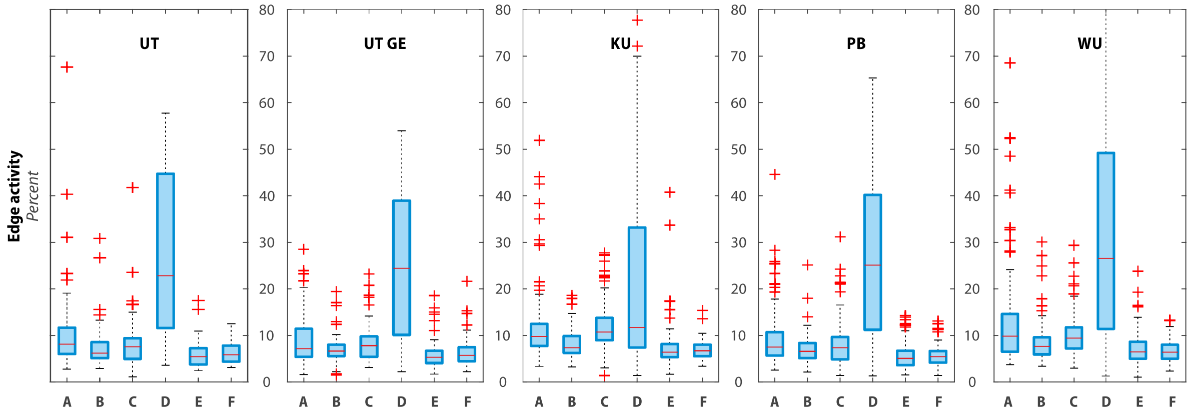

Edge activity for each scanner:
UT = University of Texas Philips, UT GE = University of Texas GE, KU = Kansas University Siemens, PB = Pennington Biomedical Research Center GE, WU = Washington University Siemens, with following preprocessing methods: A – censoring, B – censoring with global signal regression, C – aggressive AROMA, D – non-aggressive AROMA,
E – one-step AROMA (deprecated), F – SOCK

Supplementary Figure 5.

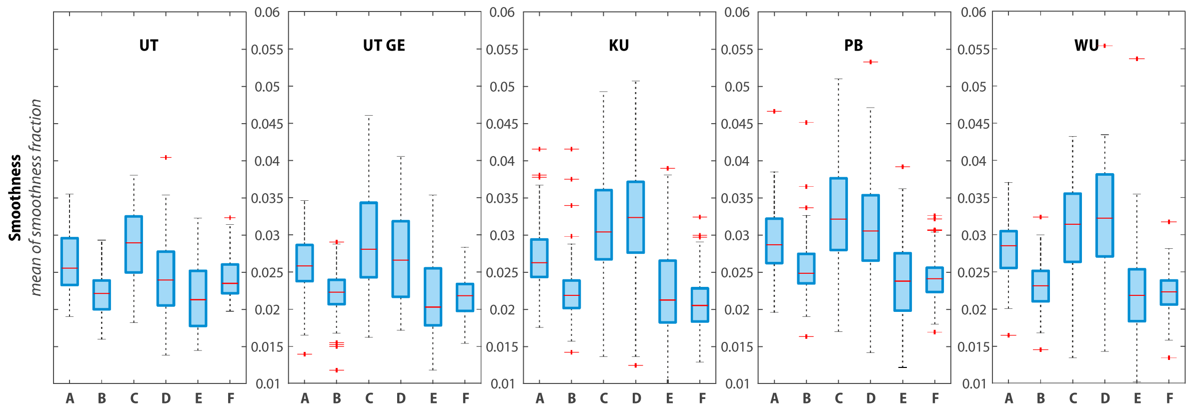

Spatial smoothness ratios for each scanner:
UT = University of Texas Philips, UT GE = University of Texas GE, KU = Kansas University Siemens, PB = Pennington Biomedical Research Center GE, WU = Washington University Siemens, with following preprocessing methods: A – censoring, B – censoring with global signal regression, C – aggressive AROMA, D – non-aggressive AROMA,
E – one-step AROMA (deprecated), F – SOCK

References

Shirer, W. R., Ryali, S., Rykhlevskaia, E., Menon, V., and Greicius, M. D. (2012). Decoding subject-driven cognitive states with whole-brain connectivity patterns. *Cereb Cortex* 22, 158–65. doi: 10.1093/cercor/bhr099.

ICA – AROMA classification output

In the following pages, you will find the 57 FSL-MELODIC IC components from the sample subject, corresponding to Figures (1) and (5) from the main manuscript.

The first 46 pages correspond to the spatial maps of the components the ICA-AROMA classifier identified as noise components for regression (partial regression = non-aggressive AROMA, full regression = aggressive AROMA). In the lower right corner, each component has the designation of what kind of noise the AROMA classifier associated with this component. HF designates high-frequency noise, Motion designates noise that is associated with subject motion and CSF designates noise that is associated with noise from cerebrospinal fluid regions.

The final 11 pages correspond to the spatial maps of the components the ICA-AROMA classifier identified as non-noise components.

AROMA – noise components for the example subject

(HF = high frequency noise, Motion = high correlation with subject motion, CSF = high correlation with CSF regions)

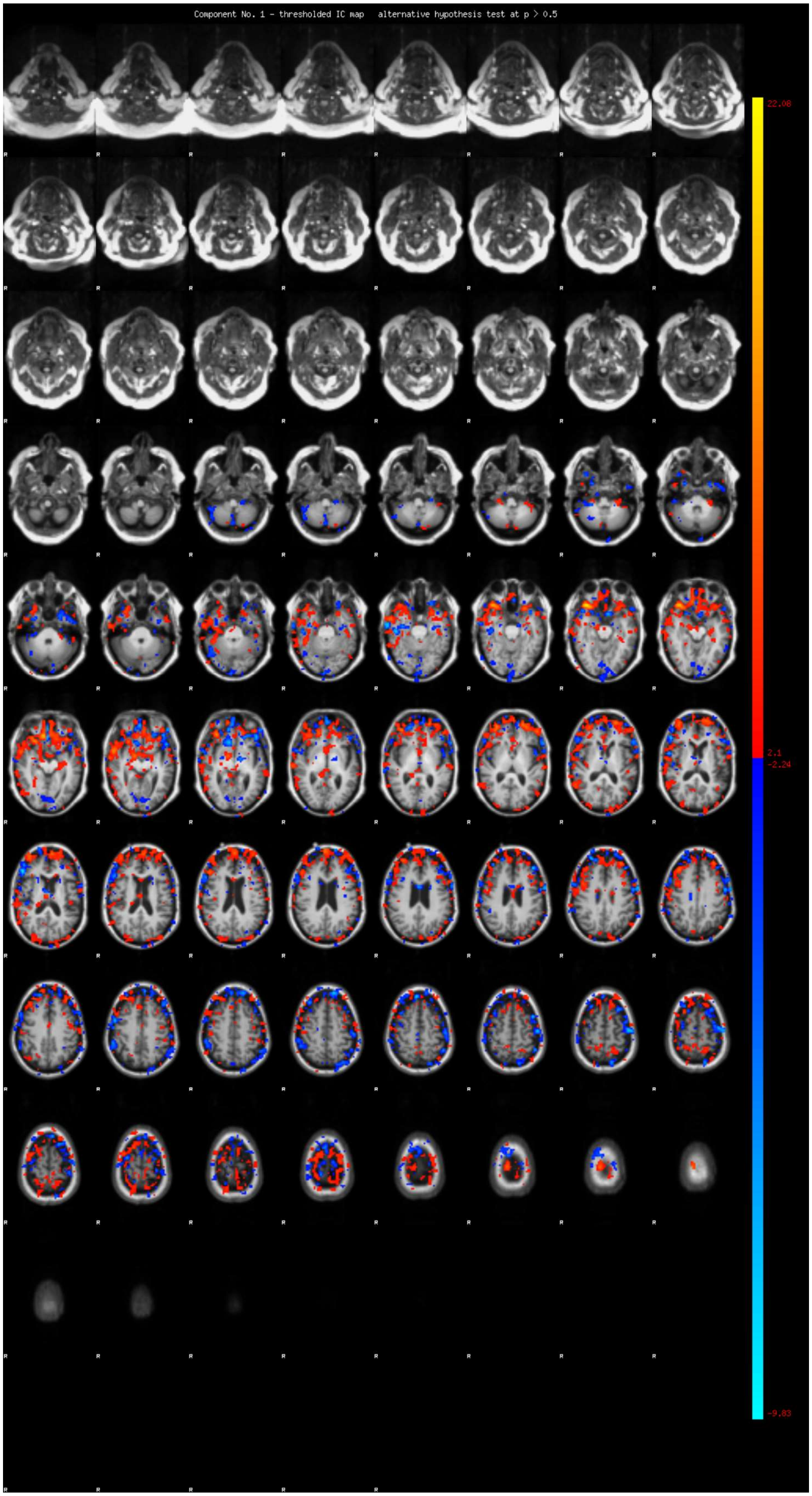

HF/Motion

AROMA – noise components for the example subject
(HF = high frequency noise, Motion = high correlation with subject motion, CSF = high correlation with CSF regions)

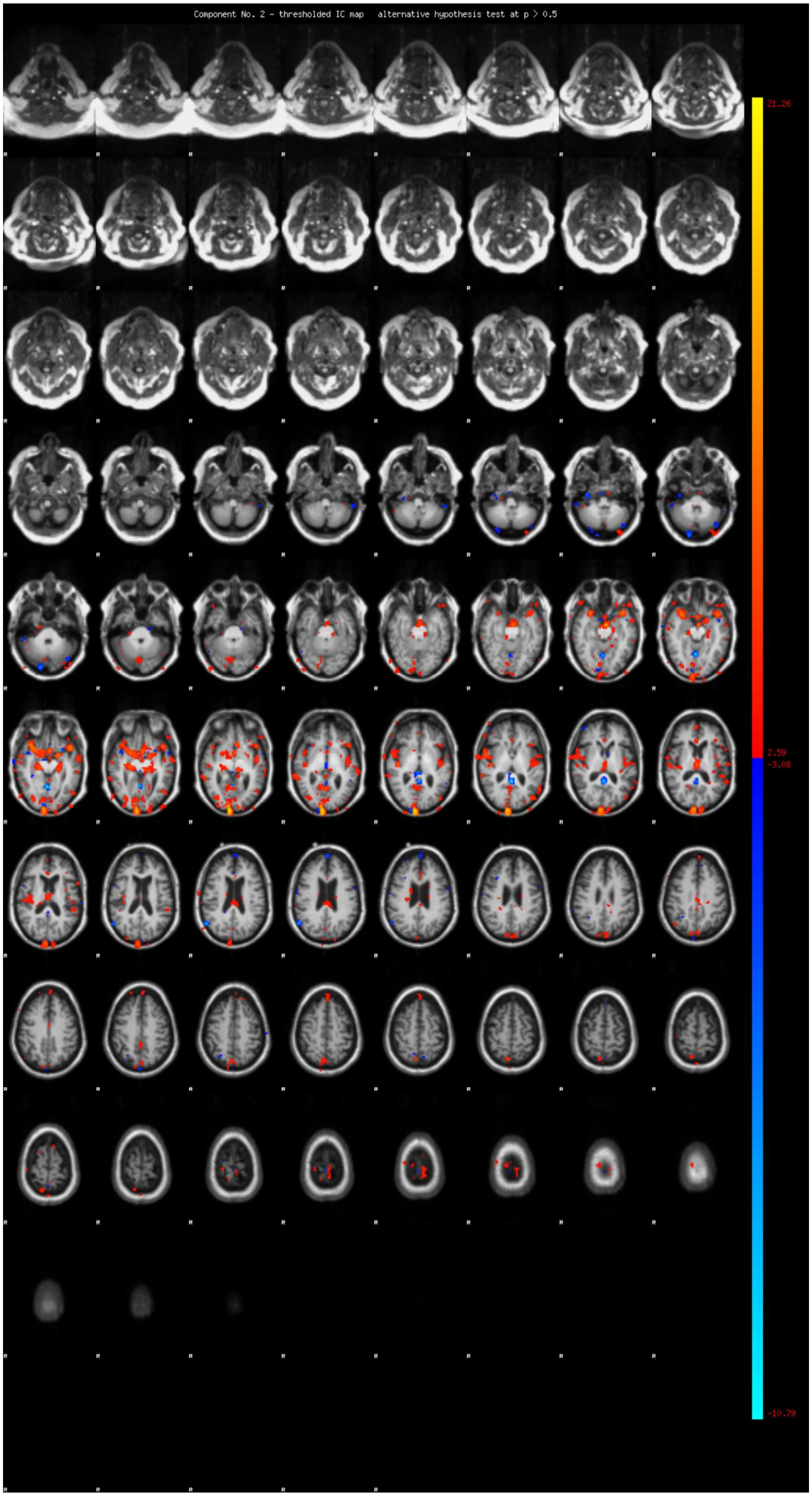

HF

AROMA – noise components for the example subject

(HF = high frequency noise, Motion = high correlation with subject motion, CSF = high correlation with CSF regions)

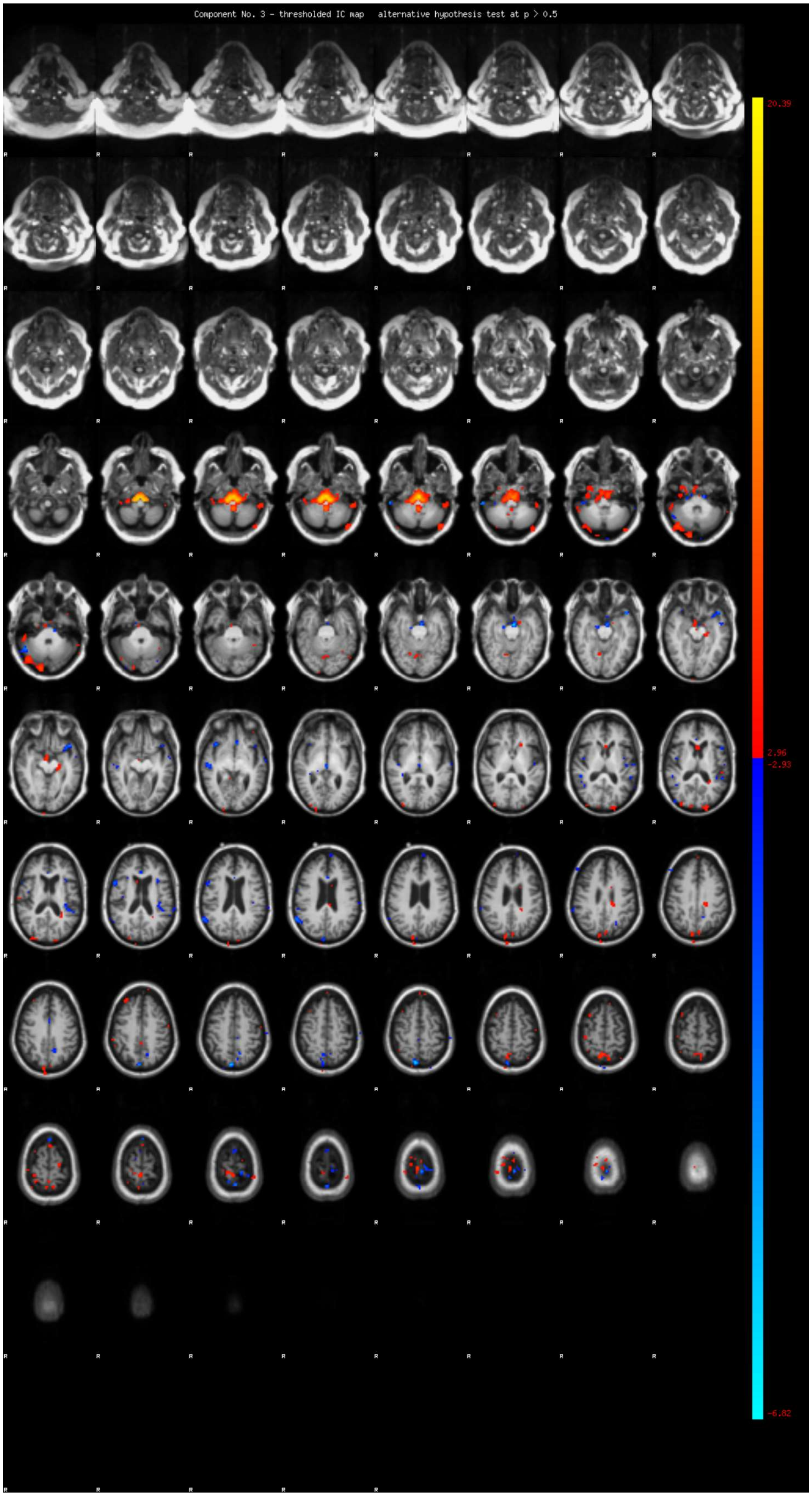

HF/Motion

AROMA – noise components for the example subject

(HF = high frequency noise, Motion = high correlation with subject motion, CSF = high correlation with CSF regions)

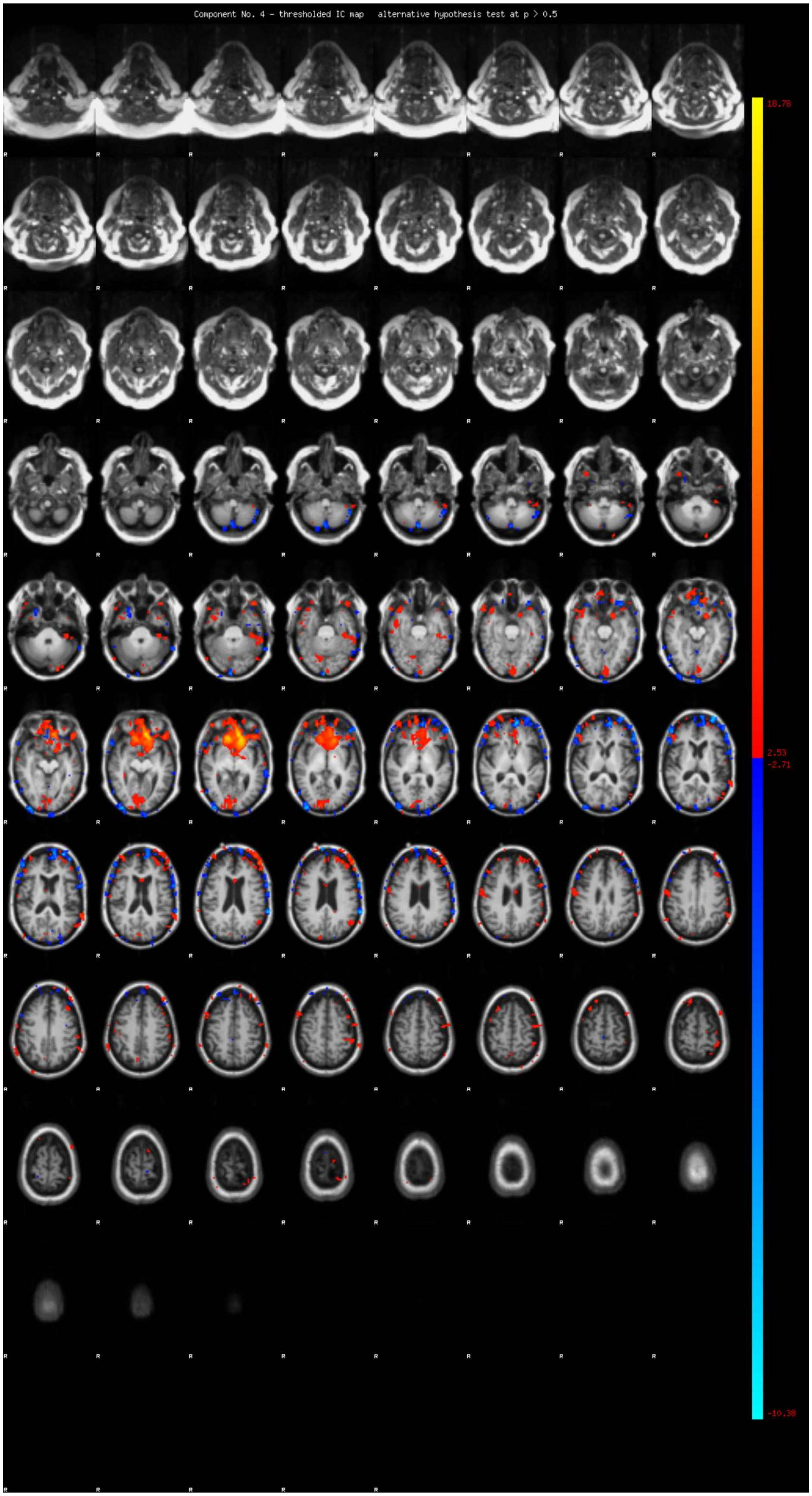

Motion

AROMA – noise components for the example subject

(HF = high frequency noise, Motion = high correlation with subject motion, CSF = high correlation with CSF regions)

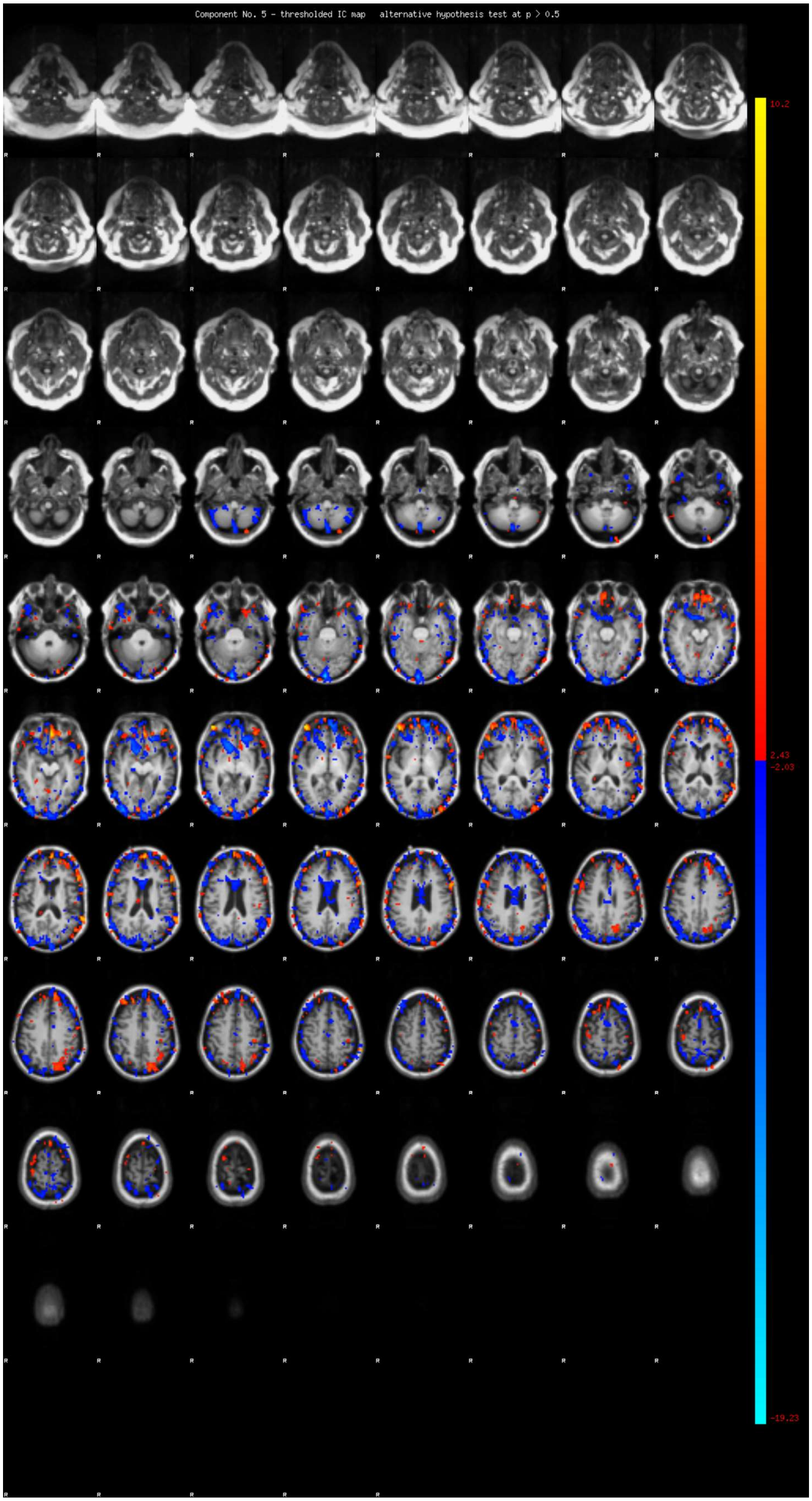

HF/Motion

AROMA – noise components for the example subject

(HF = high frequency noise, Motion = high correlation with subject motion, CSF = high correlation with CSF regions)

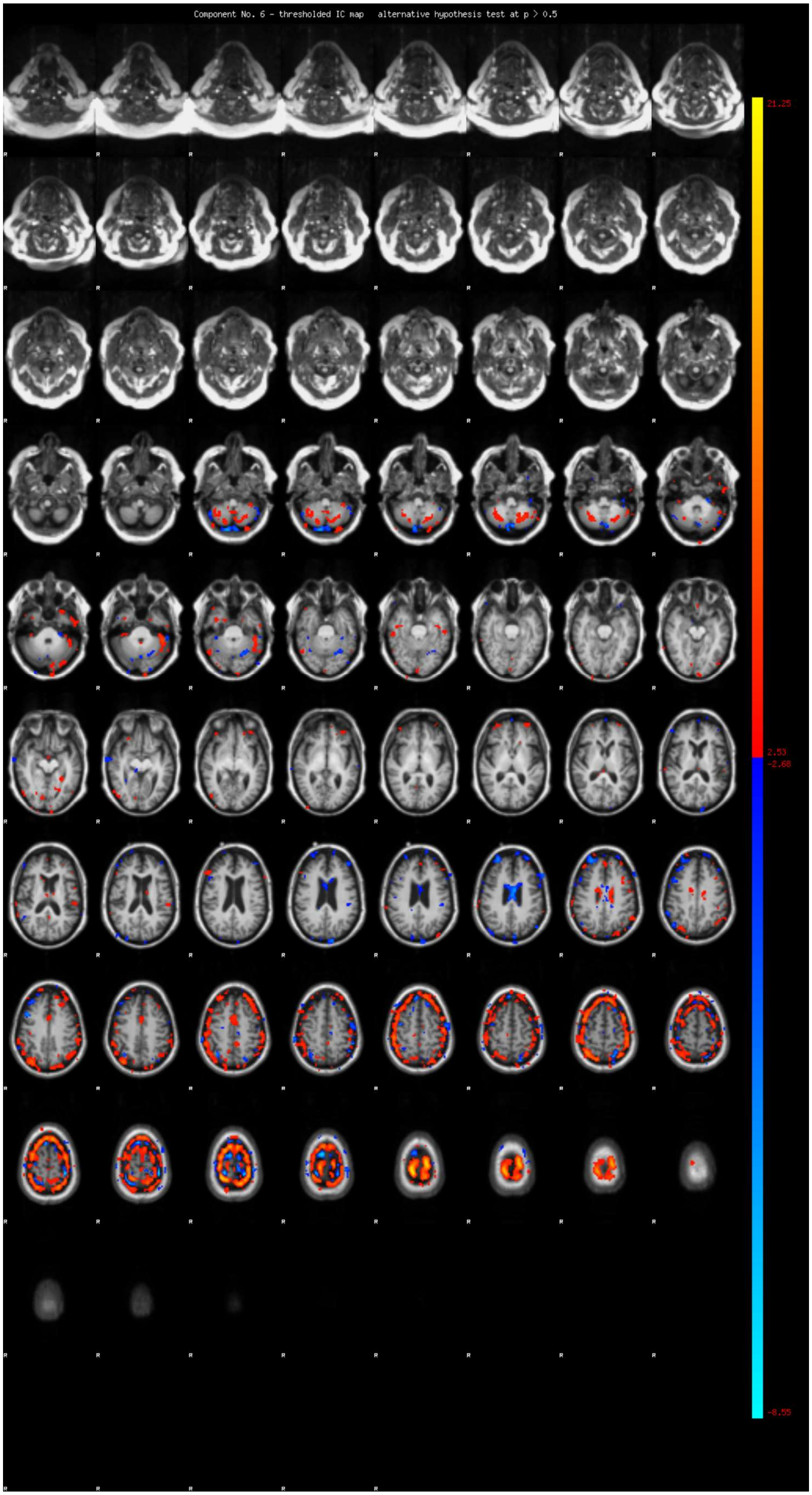

HF/Motion

AROMA – noise components for the example subject

(HF = high frequency noise, Motion = high correlation with subject motion, CSF = high correlation with CSF regions)

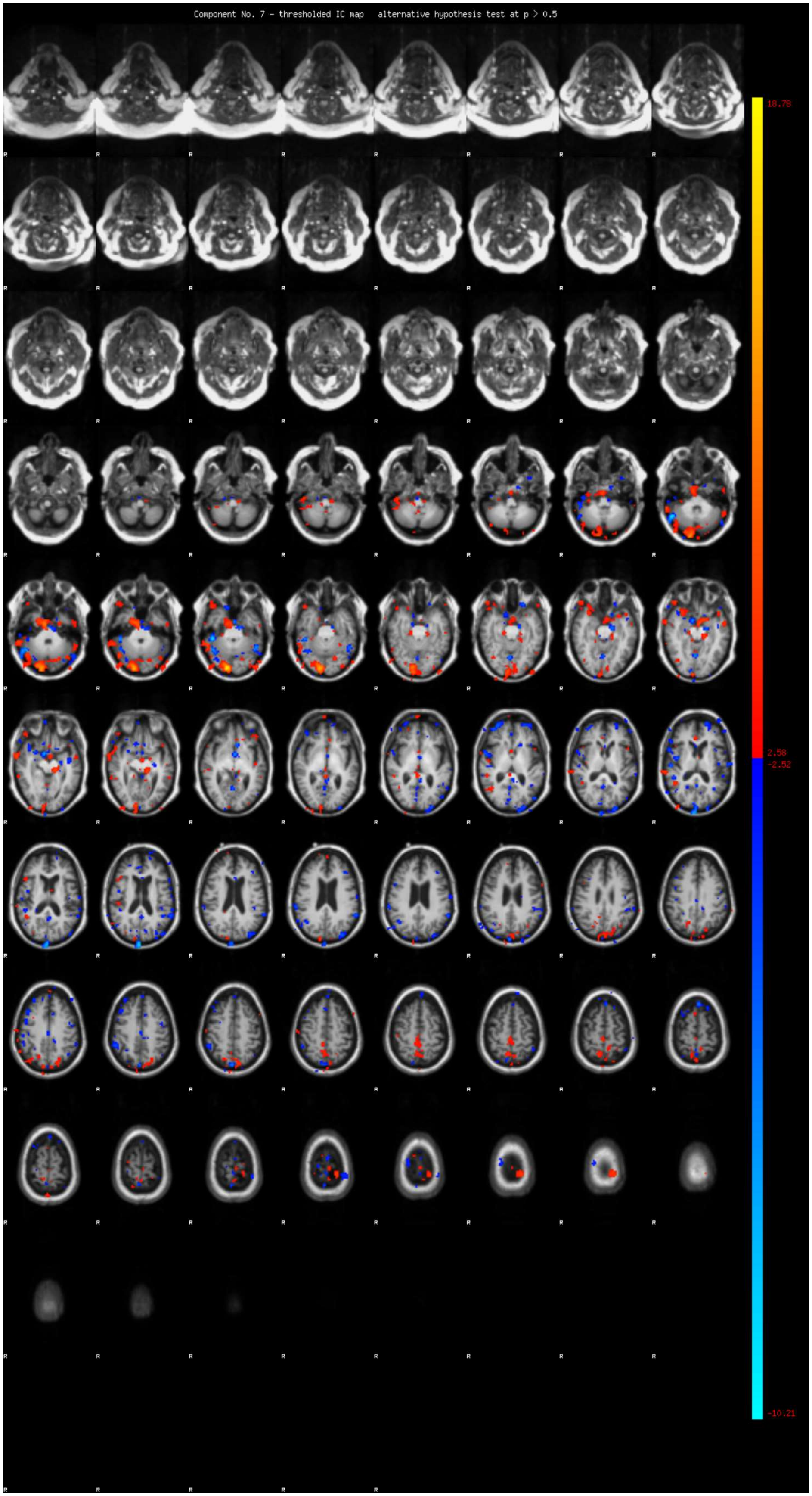

HF

AROMA – noise components for the example subject

(HF = high frequency noise, Motion = high correlation with subject motion, CSF = high correlation with CSF regions)

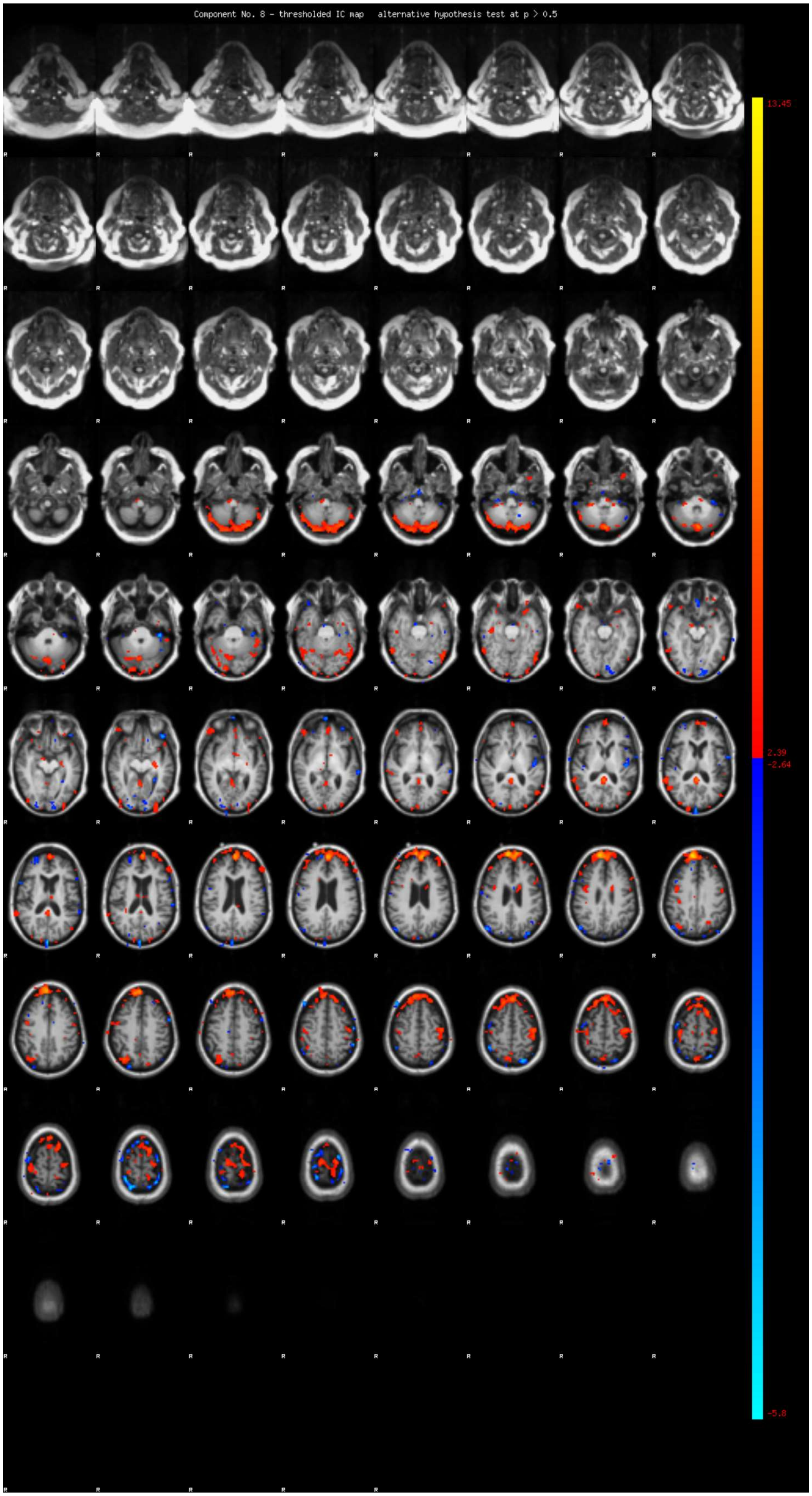

Motion

AROMA – noise components for the example subject

(HF = high frequency noise, Motion = high correlation with subject motion, CSF = high correlation with CSF regions)

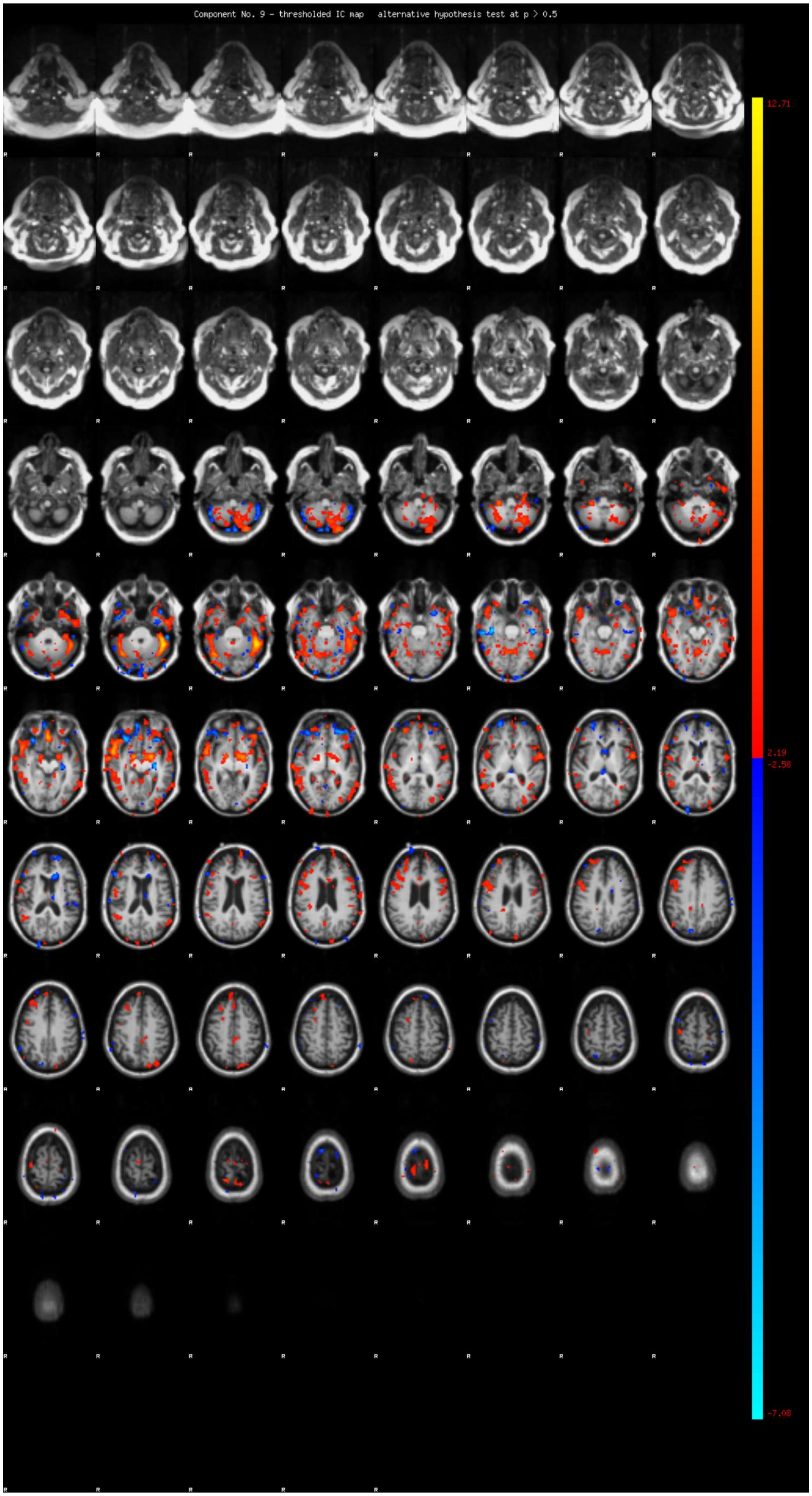

HF

AROMA – noise components for the example subject

(HF = high frequency noise, Motion = high correlation with subject motion, CSF = high correlation with CSF regions)

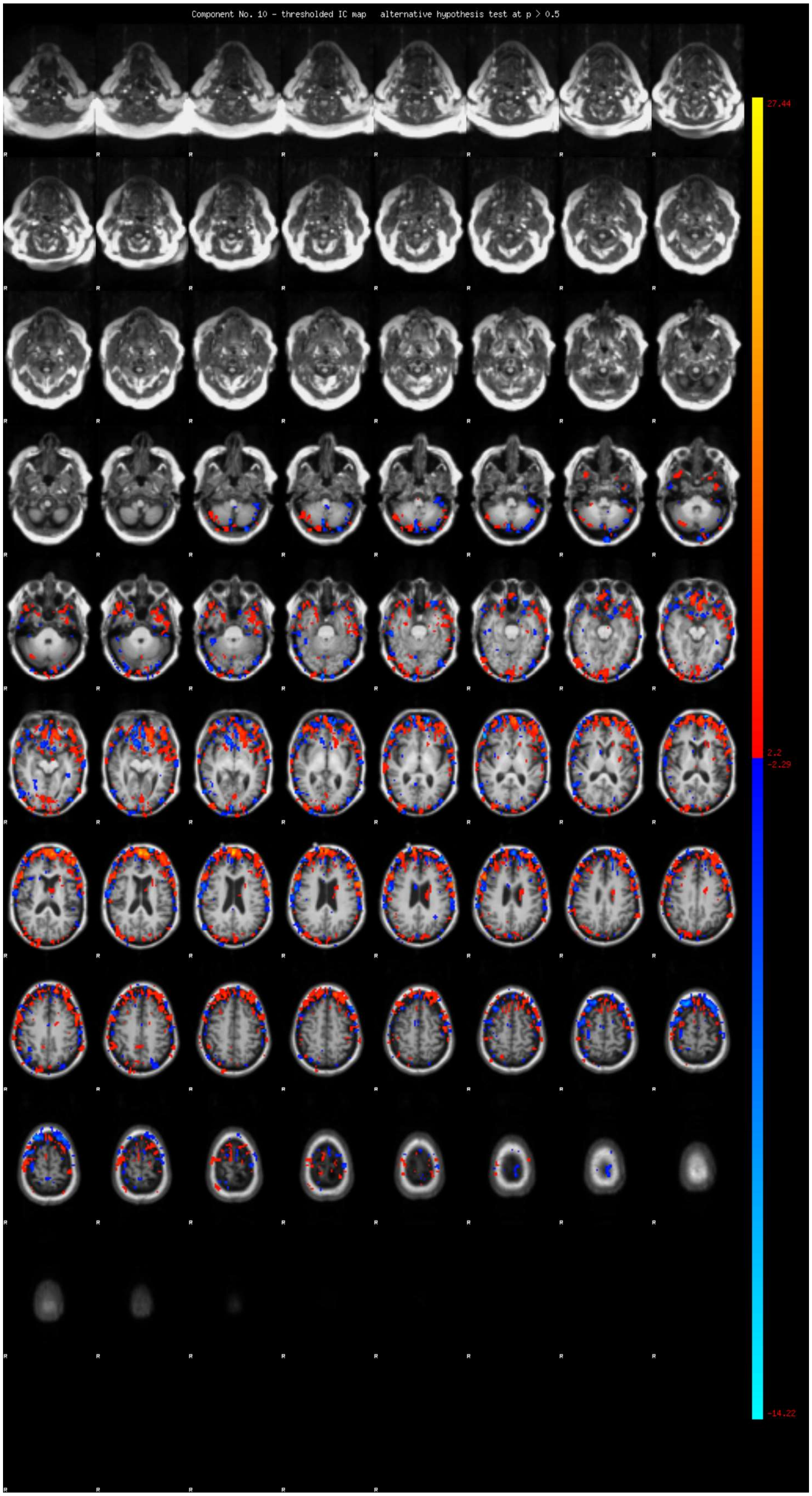

Motion

AROMA – noise components for the example subject

(HF = high frequency noise, Motion = high correlation with subject motion, CSF = high correlation with CSF regions)

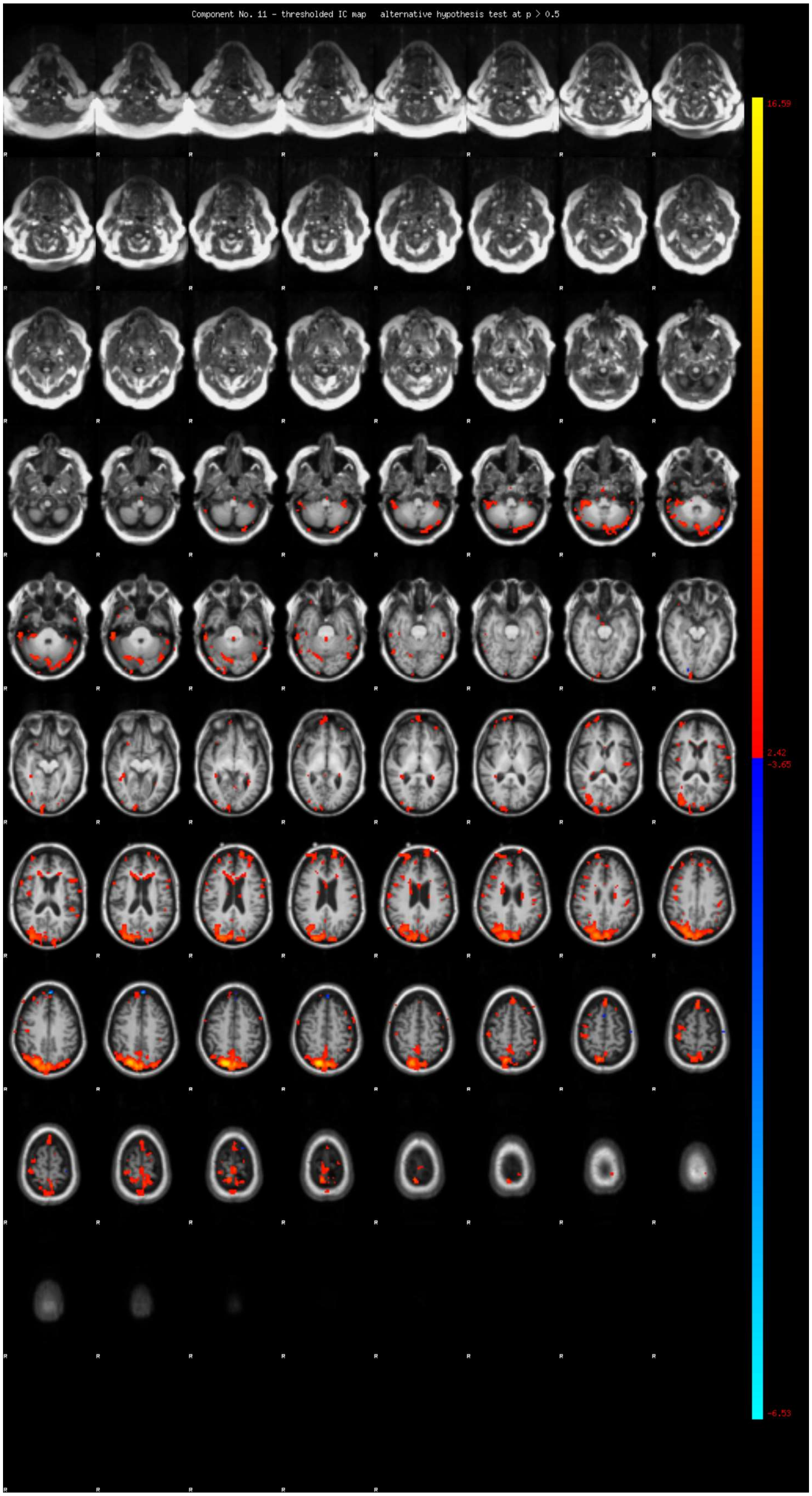

HF

AROMA – noise components for the example subject

(HF = high frequency noise, Motion = high correlation with subject motion, CSF = high correlation with CSF regions)

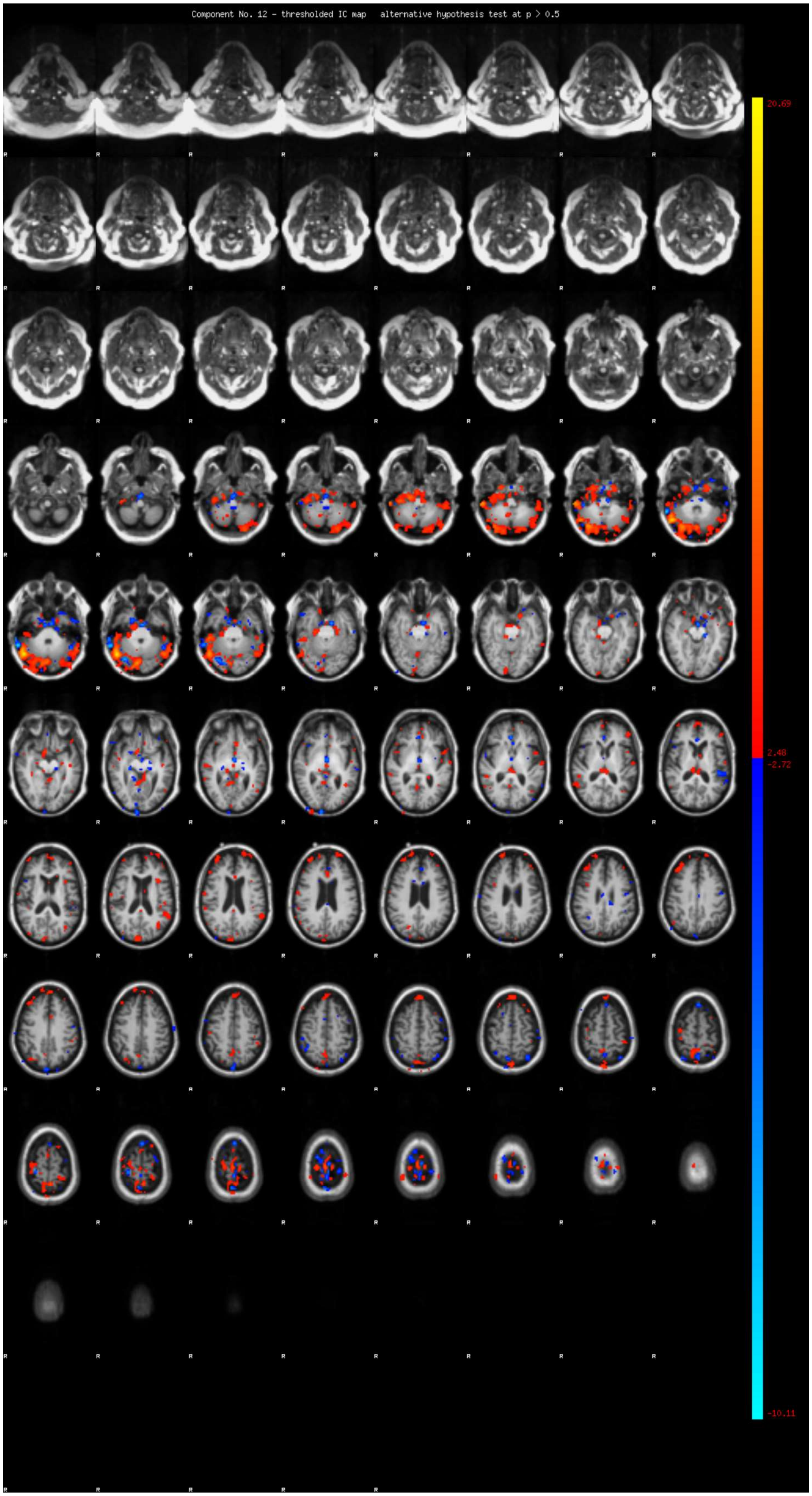

HF

AROMA – noise components for the example subject

(HF = high frequency noise, Motion = high correlation with subject motion, CSF = high correlation with CSF regions)

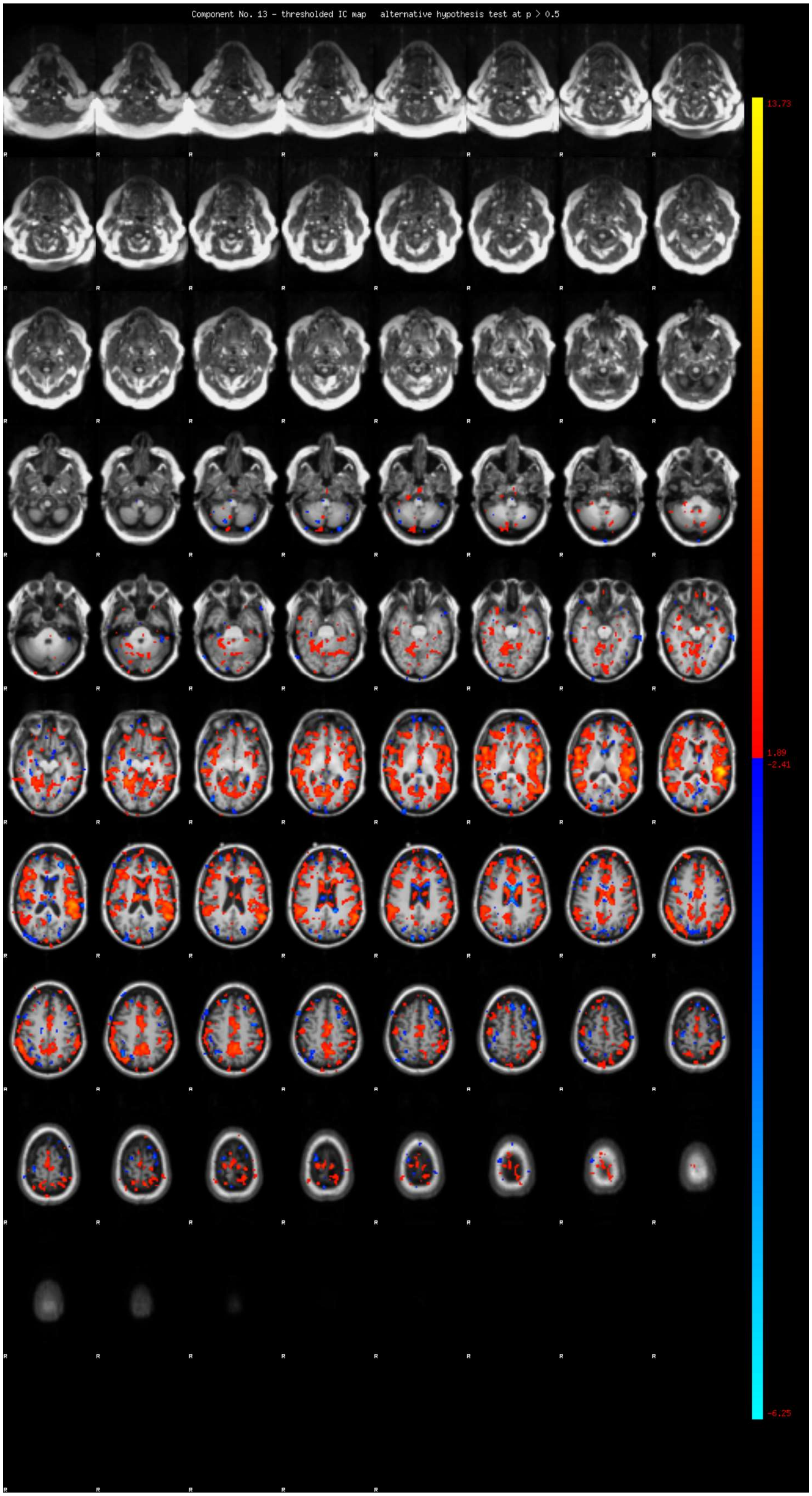

HF

AROMA – noise components for the example subject

(HF = high frequency noise, Motion = high correlation with subject motion, CSF = high correlation with CSF regions)

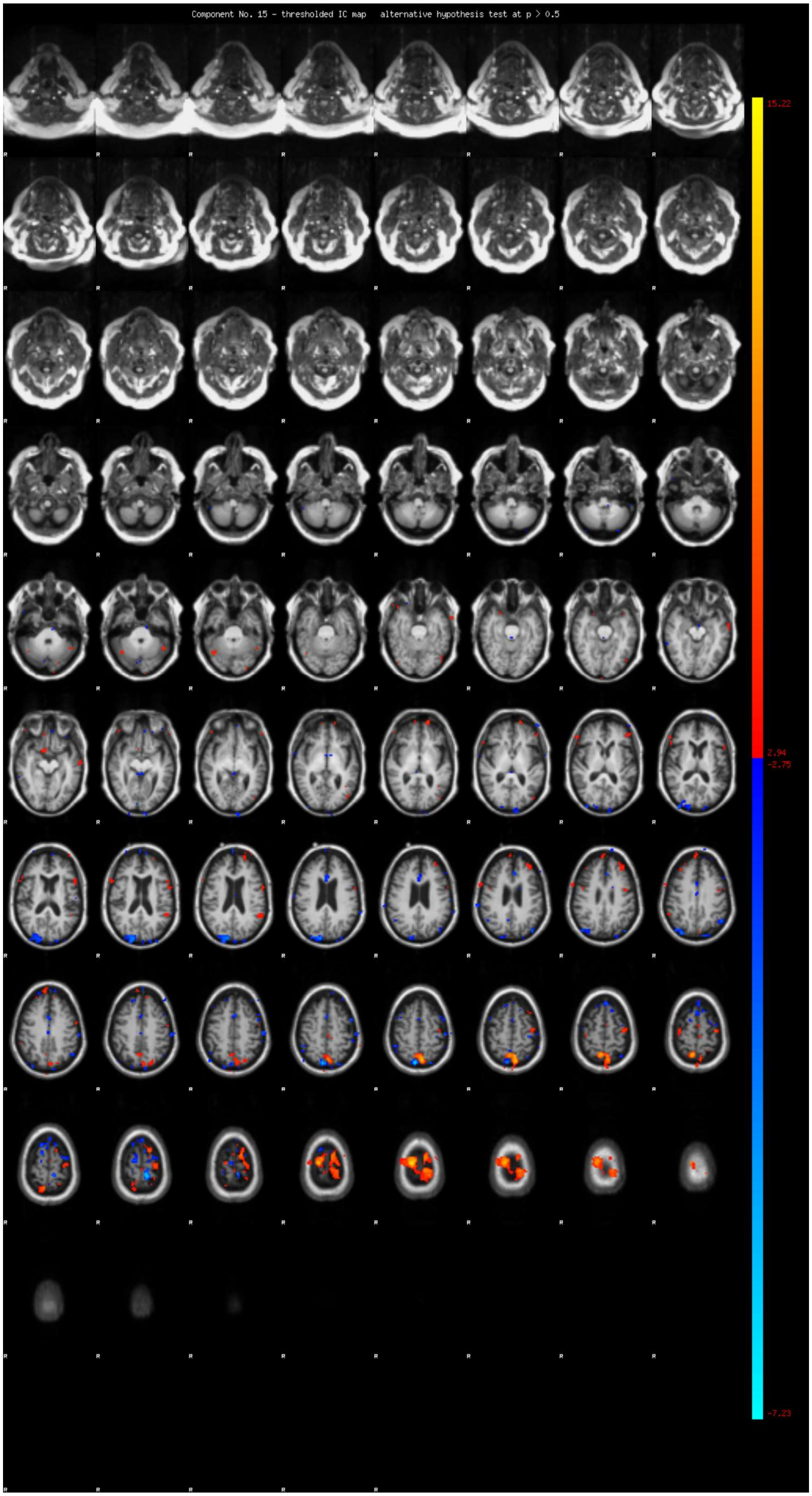

Motion

AROMA – noise components for the example subject

(HF = high frequency noise, Motion = high correlation with subject motion, CSF = high correlation with CSF regions)

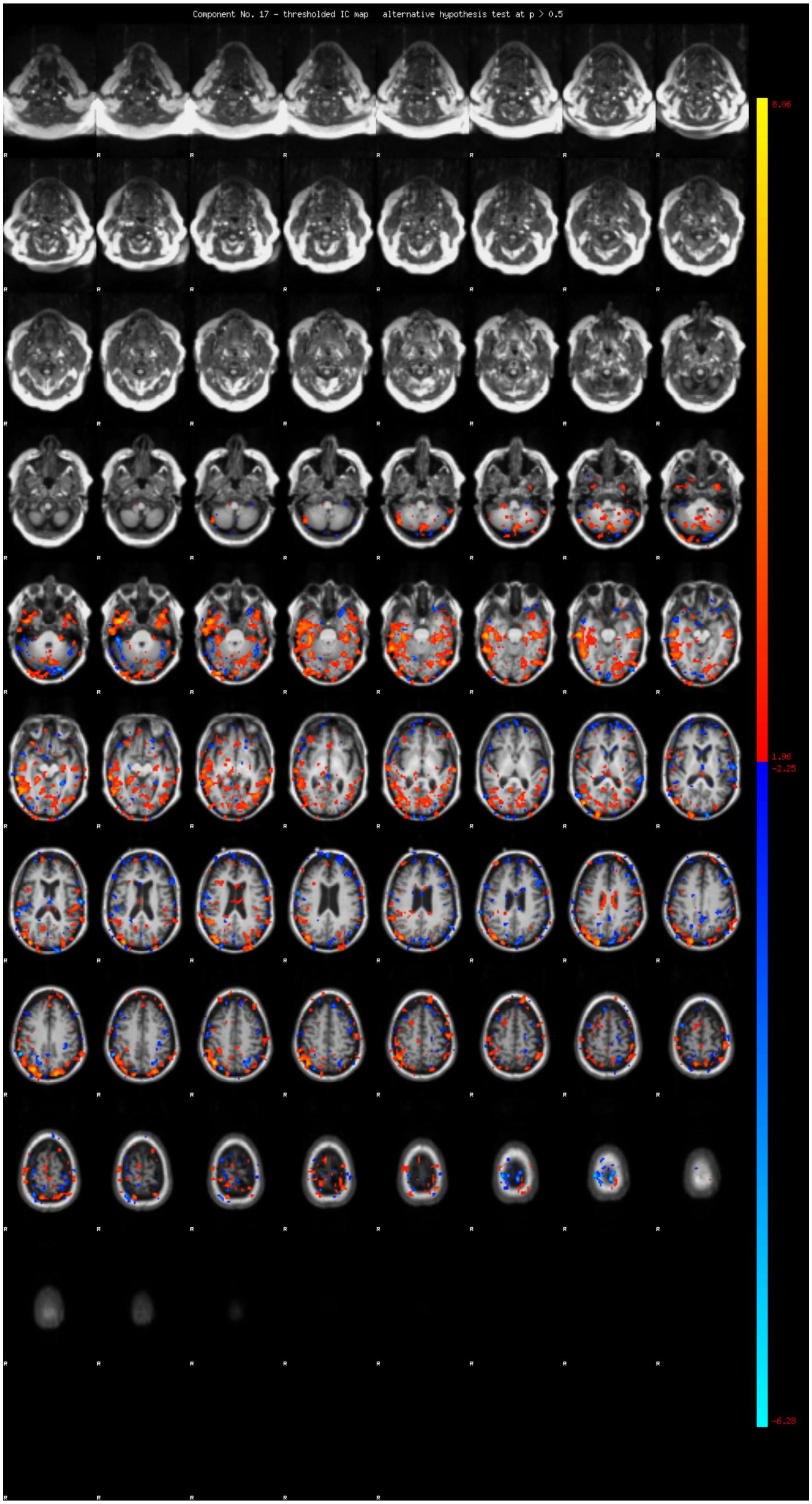

HF

AROMA – noise components for the example subject

(HF = high frequency noise, Motion = high correlation with subject motion, CSF = high correlation with CSF regions)

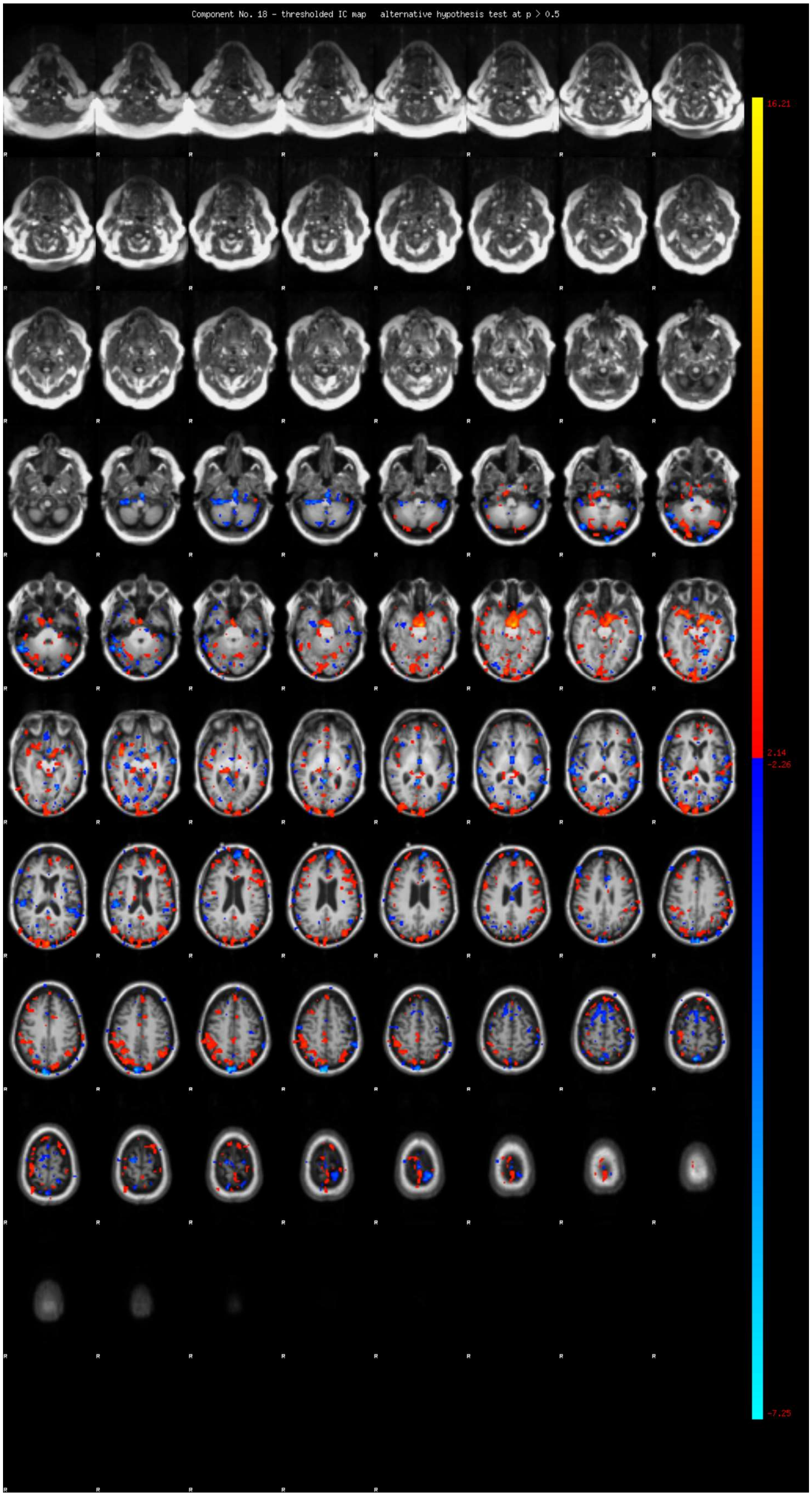

HF

AROMA – noise components for the example subject

(HF = high frequency noise, Motion = high correlation with subject motion, CSF = high correlation with CSF regions)

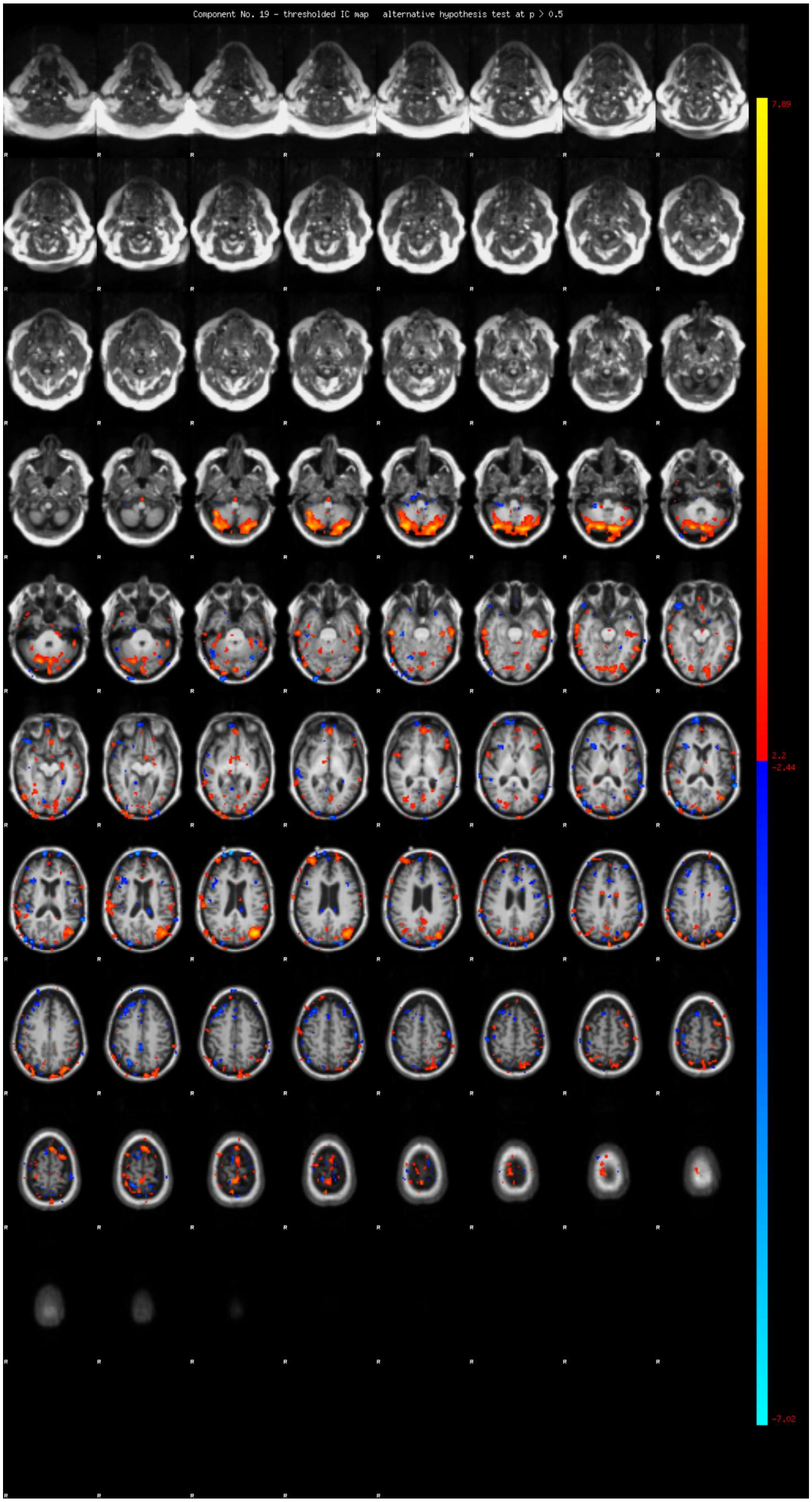

Motion

AROMA – noise components for the example subject

(HF = high frequency noise, Motion = high correlation with subject motion, CSF = high correlation with CSF regions)

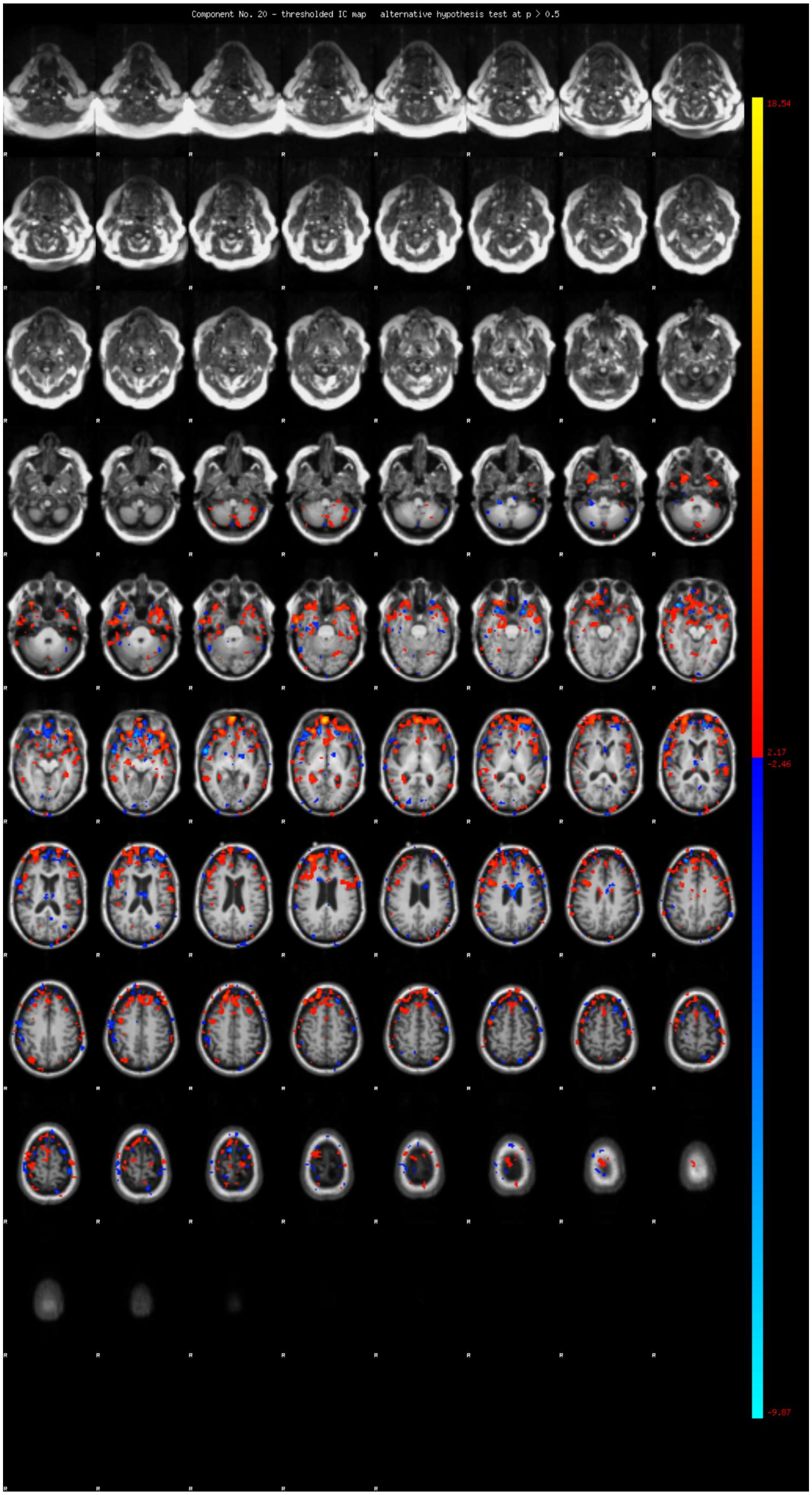

HF/Motion

AROMA – noise components for the example subject

(HF = high frequency noise, Motion = high correlation with subject motion, CSF = high correlation with CSF regions)

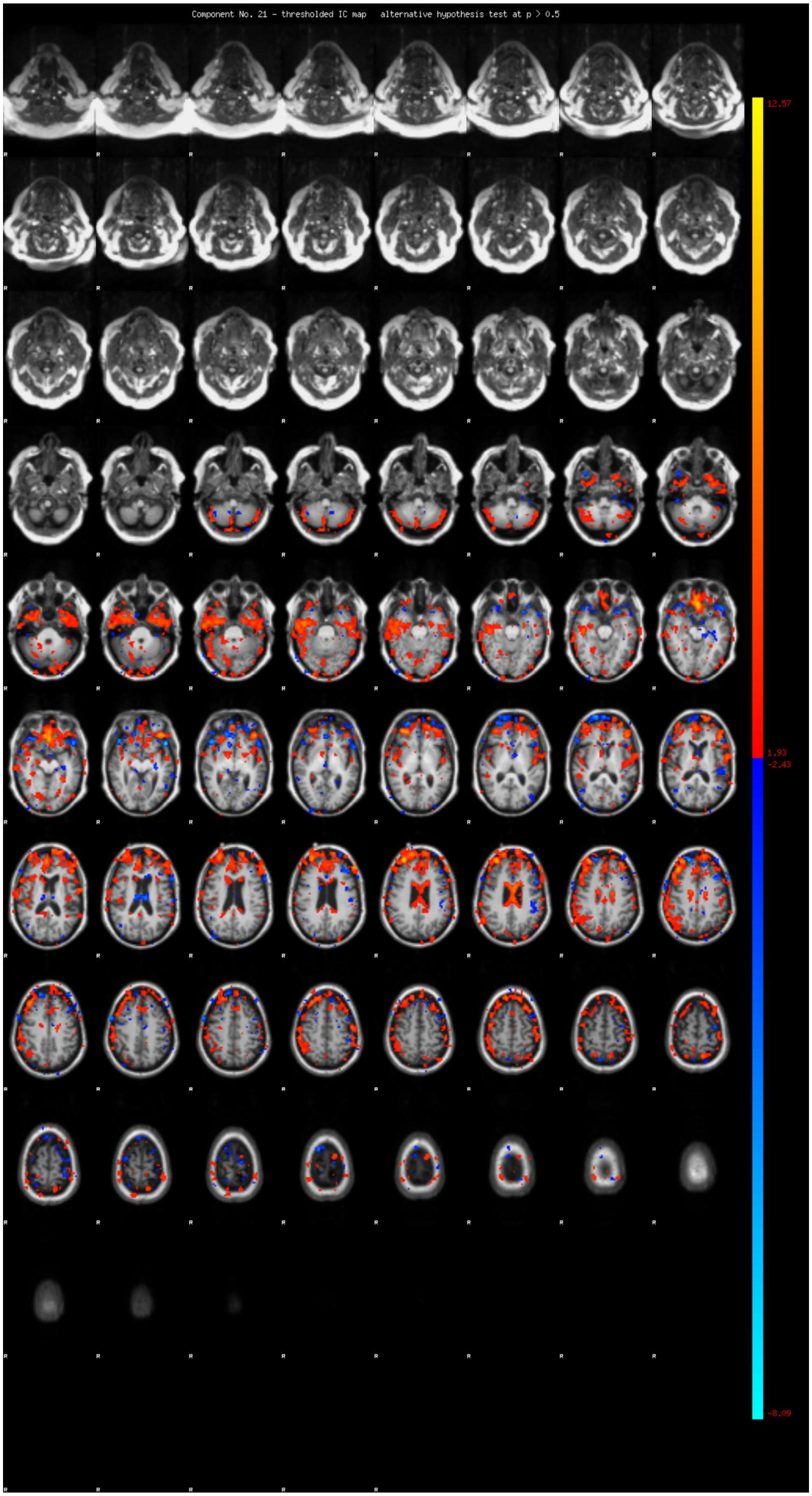

HF/Motion

AROMA – noise components for the example subject

(HF = high frequency noise, Motion = high correlation with subject motion, CSF = high correlation with CSF regions)

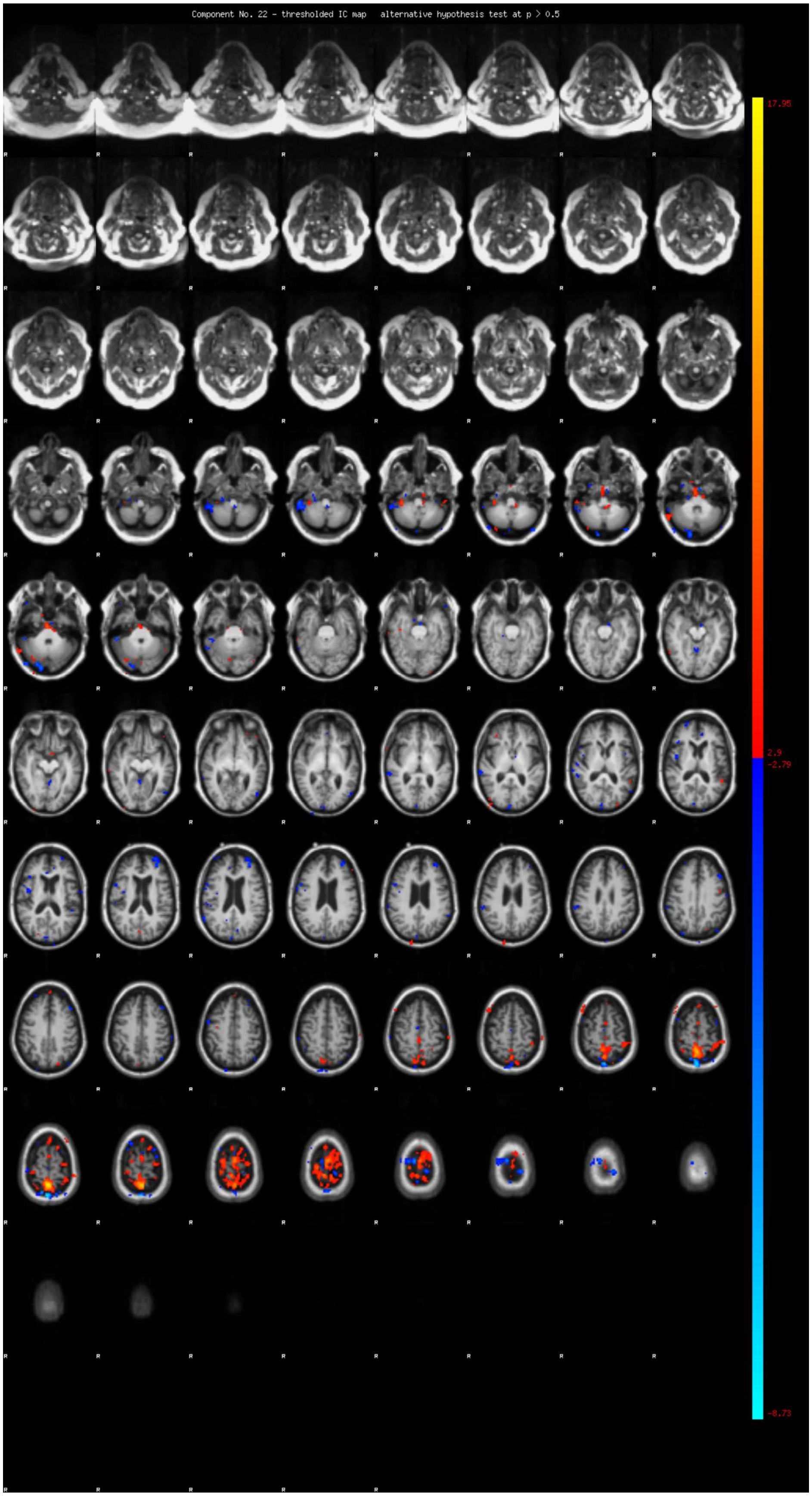

HF/Motion

AROMA – noise components for the example subject

(HF = high frequency noise, Motion = high correlation with subject motion, CSF = high correlation with CSF regions)

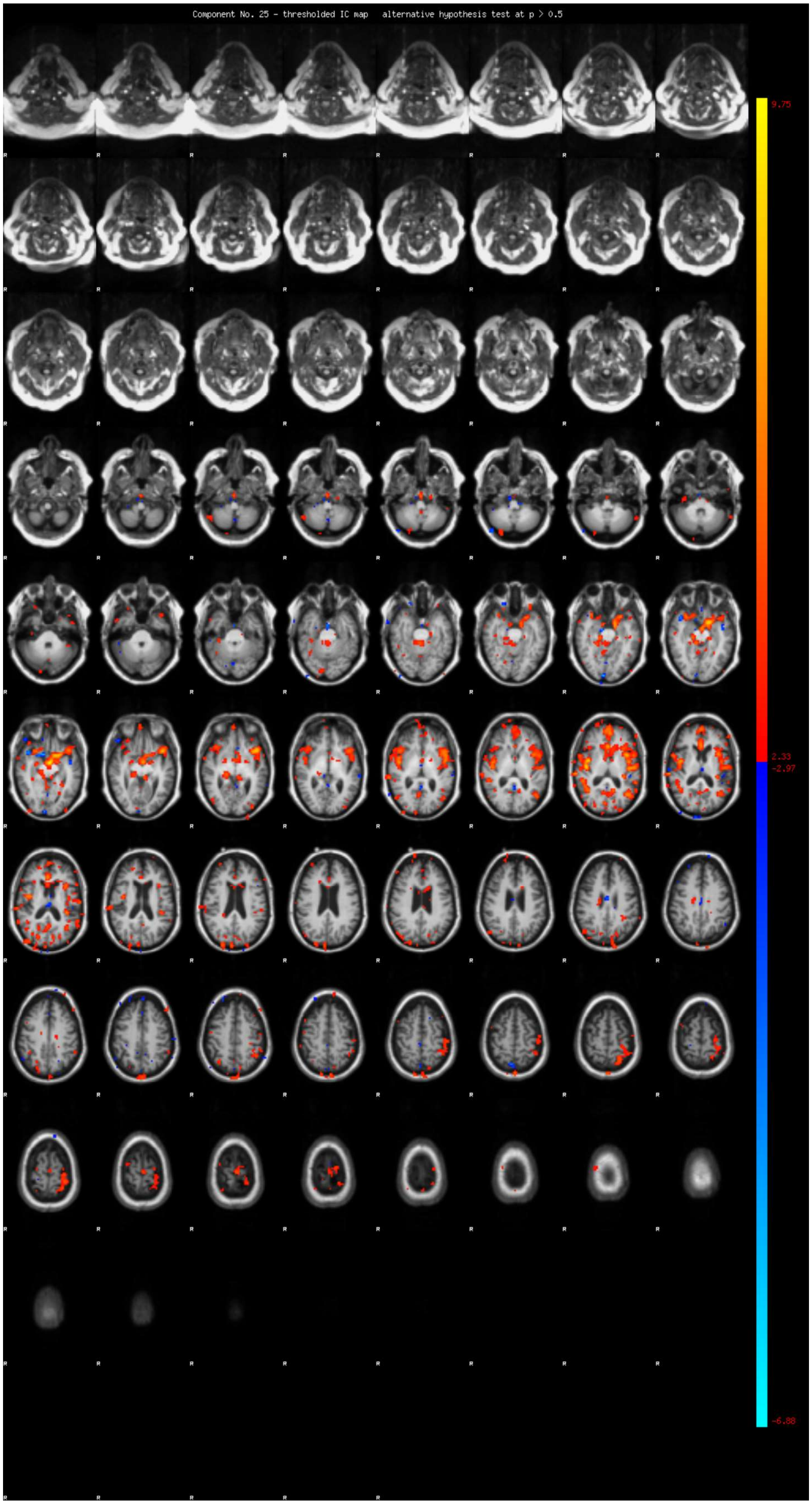

HF/Motion

AROMA – noise components for the example subject

(HF = high frequency noise, Motion = high correlation with subject motion, CSF = high correlation with CSF regions)

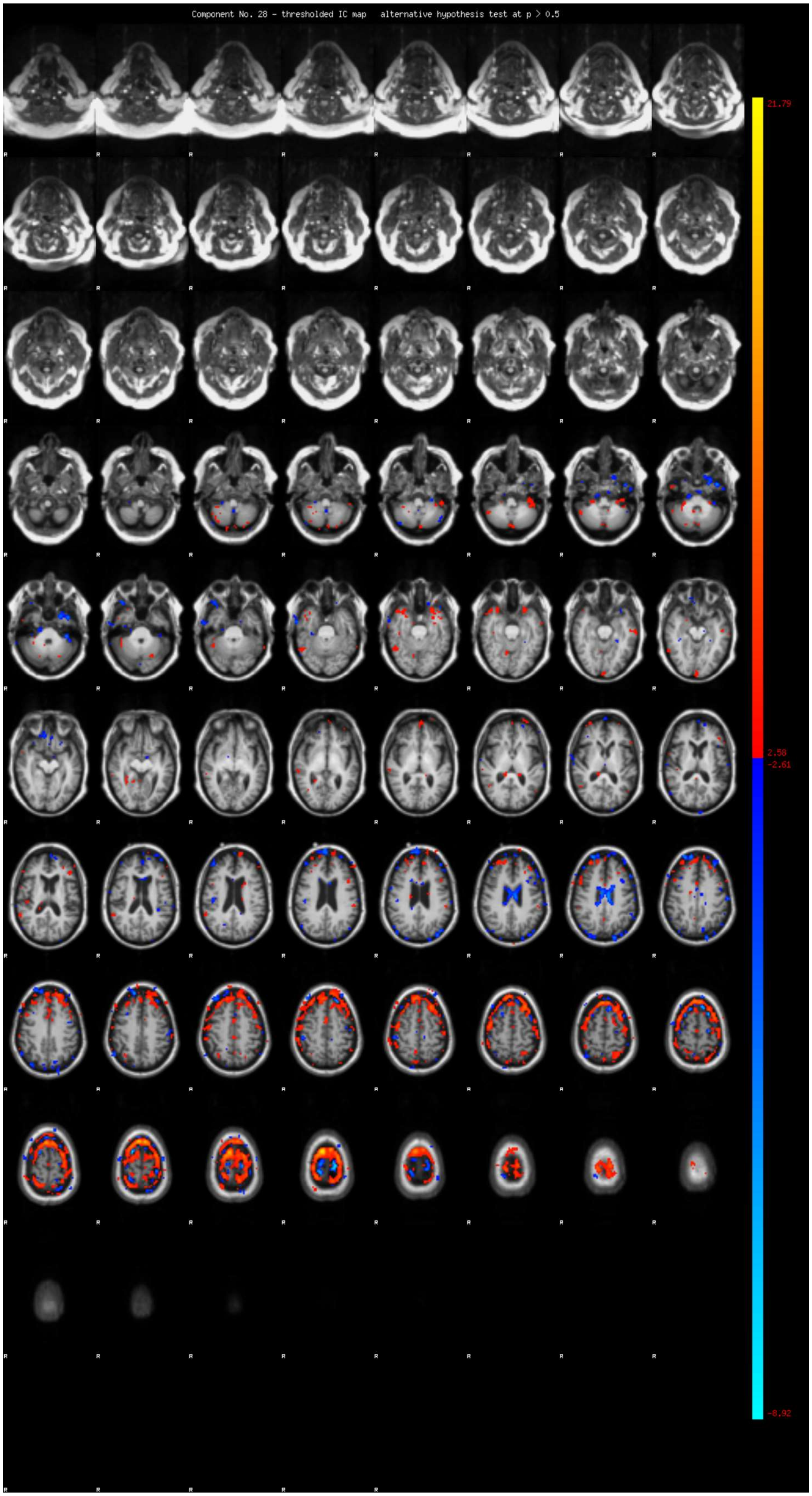

HF/Motion

AROMA – noise components for the example subject

(HF = high frequency noise, Motion = high correlation with subject motion, CSF = high correlation with CSF regions)

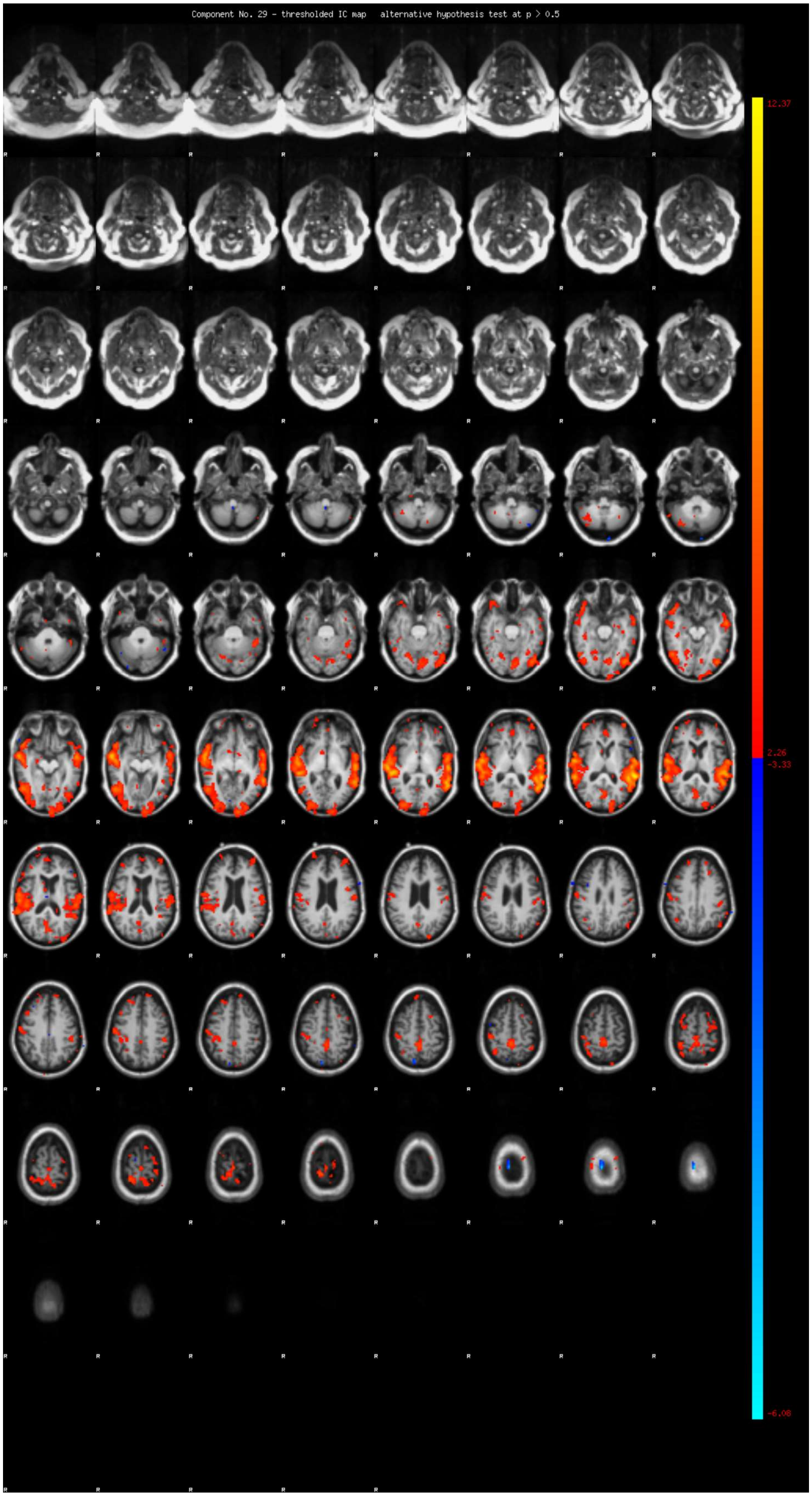

Motion

AROMA – noise components for the example subject

(HF = high frequency noise, Motion = high correlation with subject motion, CSF = high correlation with CSF regions)

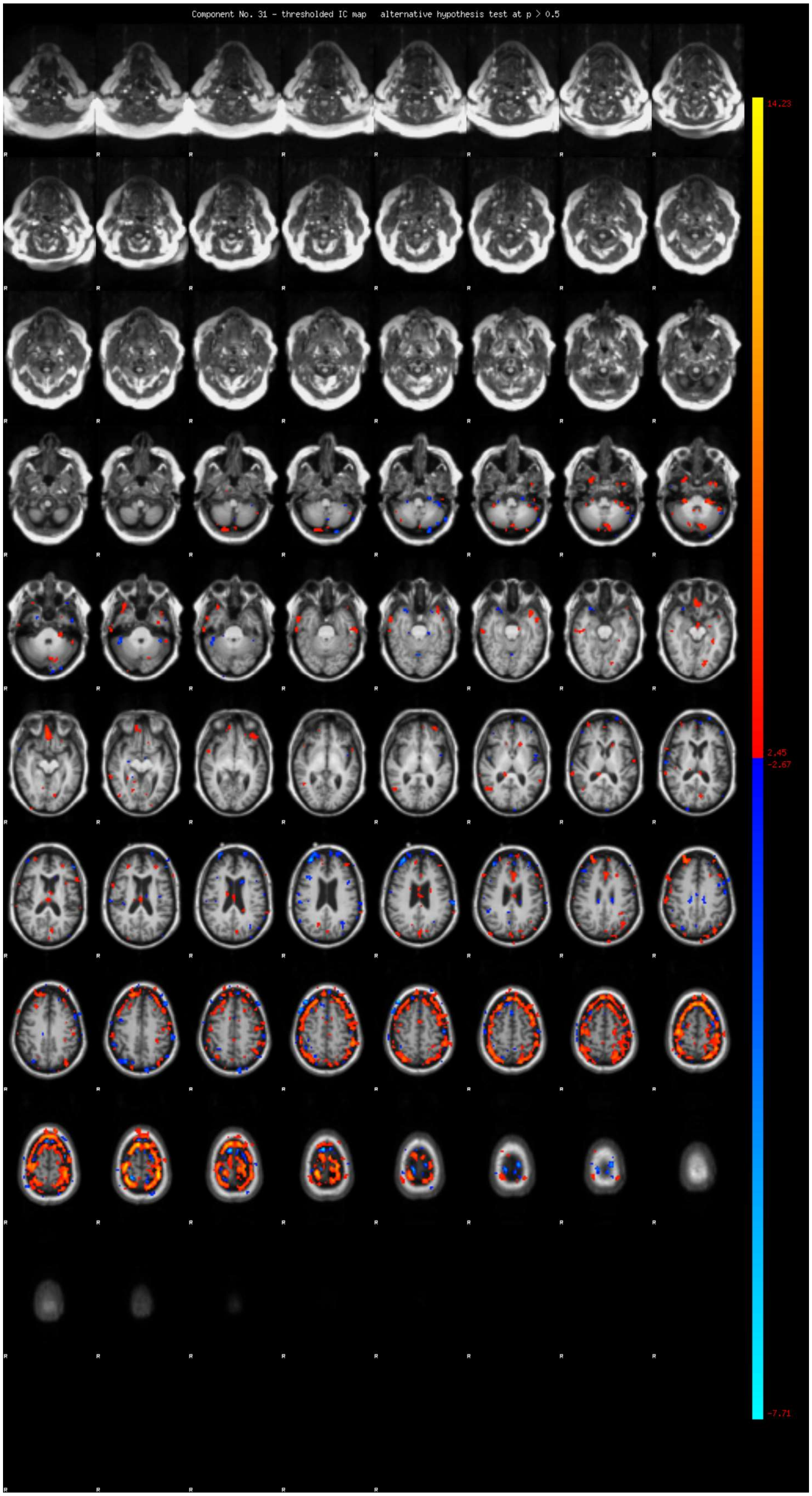

HF/Motion

AROMA – noise components for the example subject

(HF = high frequency noise, Motion = high correlation with subject motion, CSF = high correlation with CSF regions)

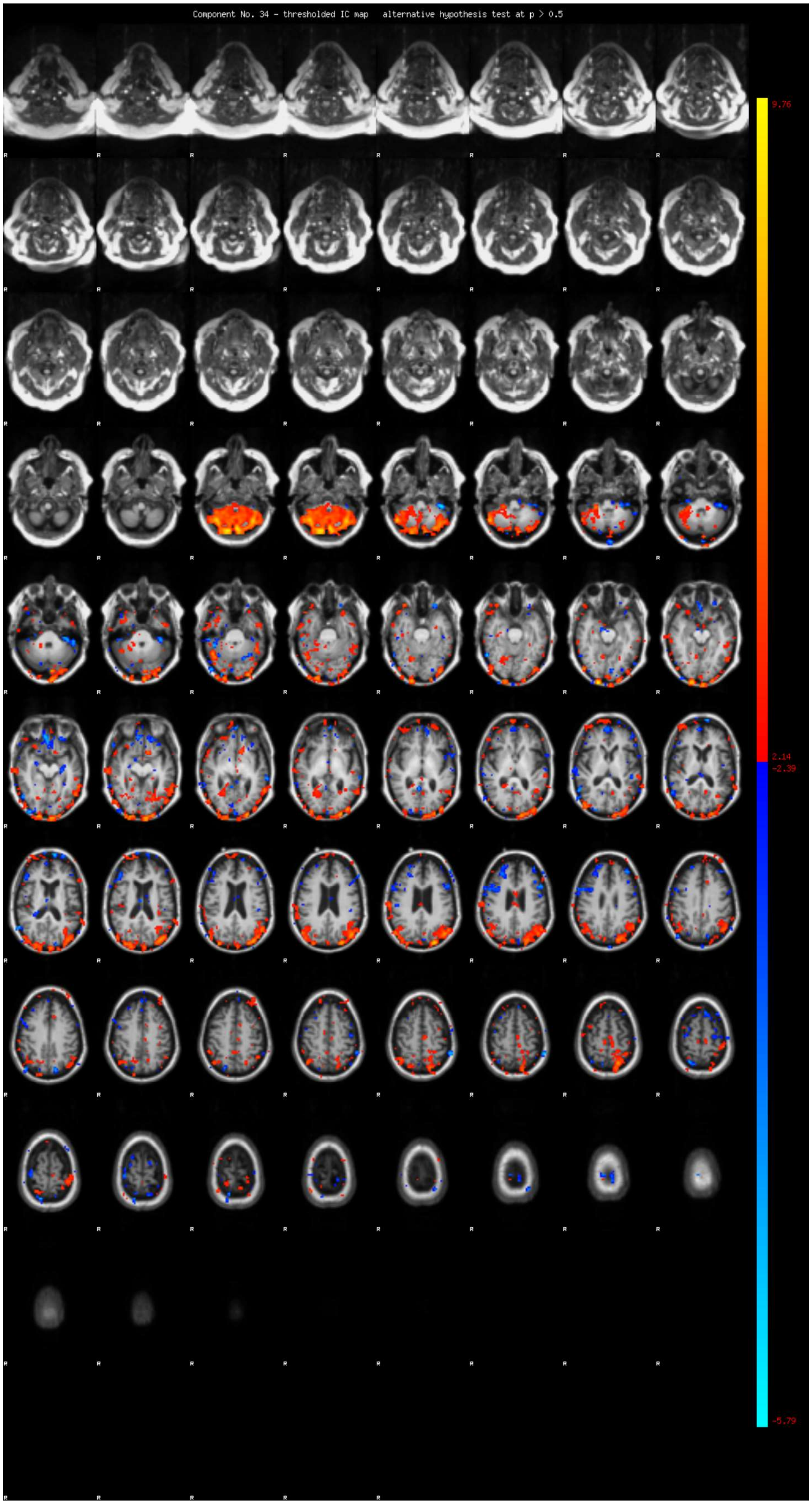

Motion

AROMA – noise components for the example subject

(HF = high frequency noise, Motion = high correlation with subject motion, CSF = high correlation with CSF regions)

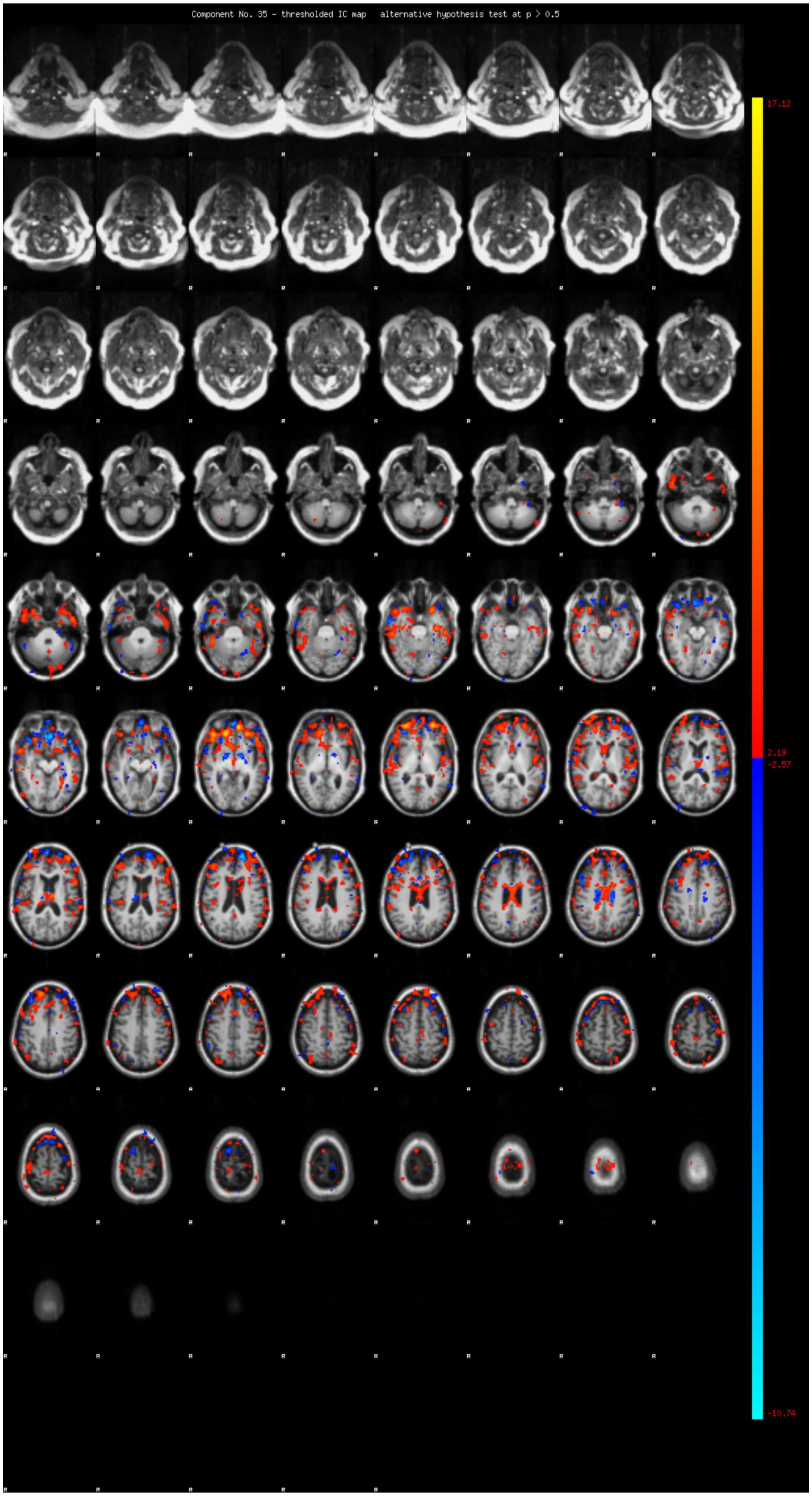

HF

AROMA – noise components for the example subject

(HF = high frequency noise, Motion = high correlation with subject motion, CSF = high correlation with CSF regions)

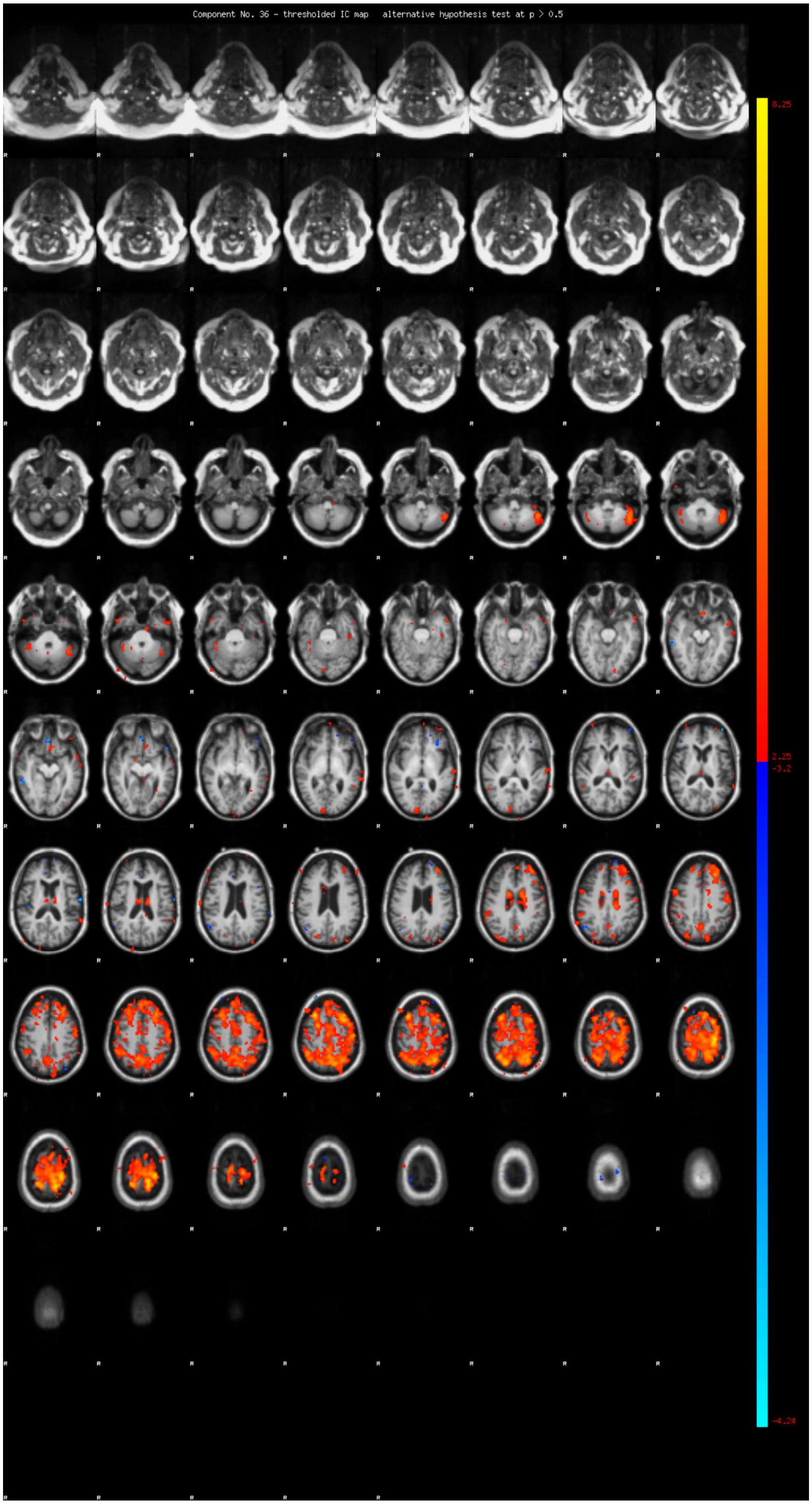

HF

AROMA – noise components for the example subject

(HF = high frequency noise, Motion = high correlation with subject motion, CSF = high correlation with CSF regions)

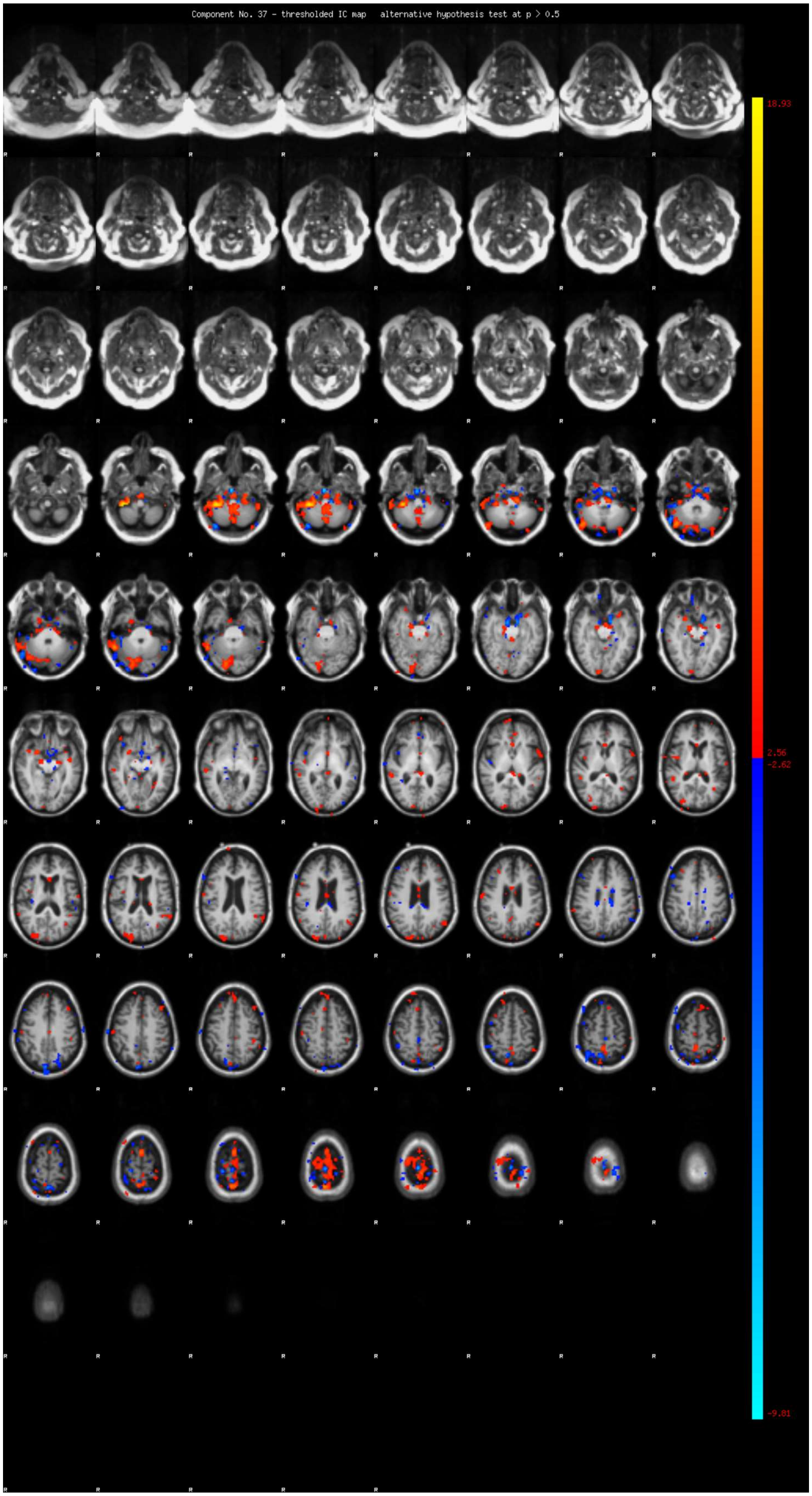

HF

AROMA – noise components for the example subject

(HF = high frequency noise, Motion = high correlation with subject motion, CSF = high correlation with CSF regions)

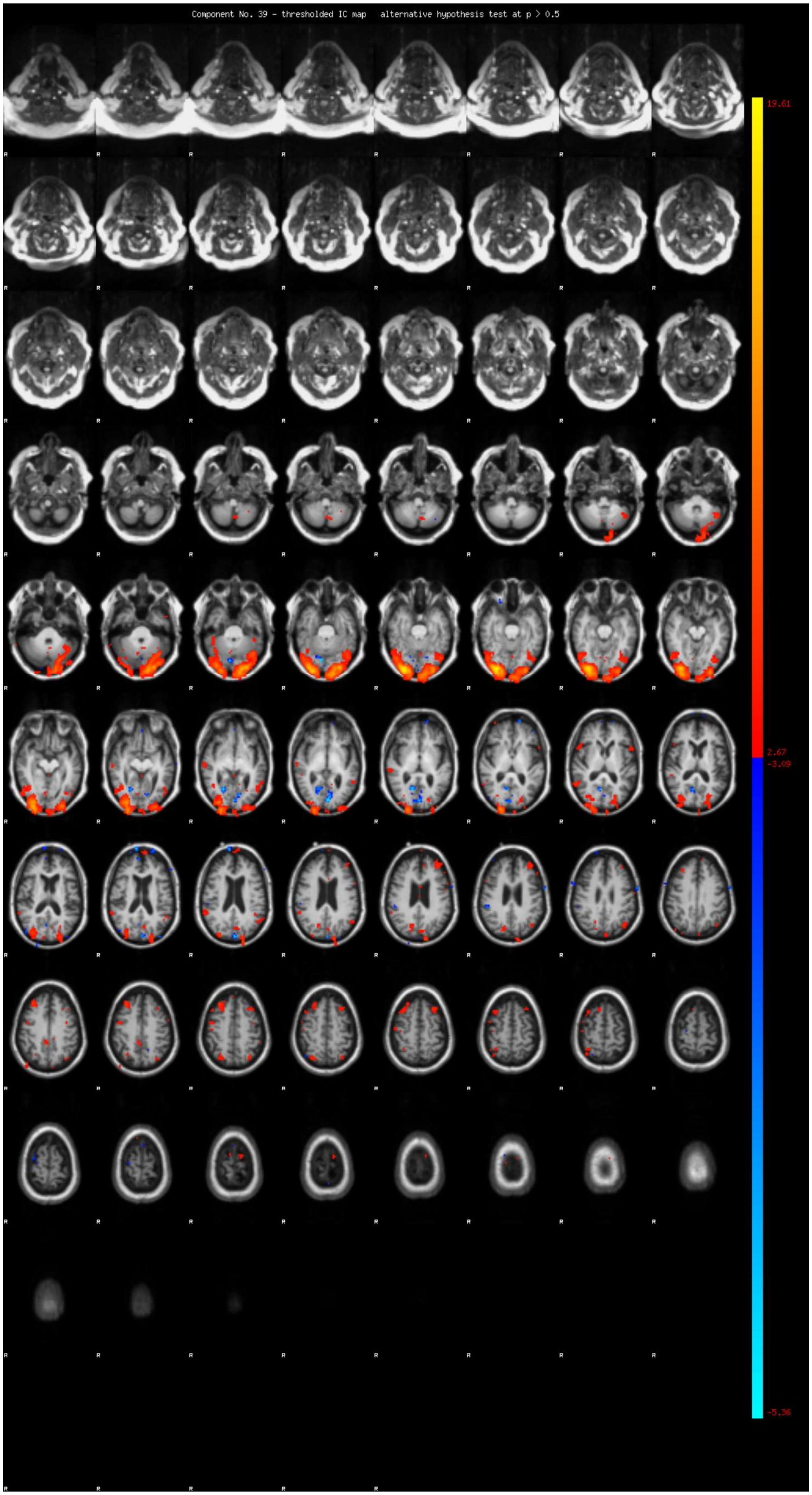

Motion

AROMA – noise components for the example subject

(HF = high frequency noise, Motion = high correlation with subject motion, CSF = high correlation with CSF regions)

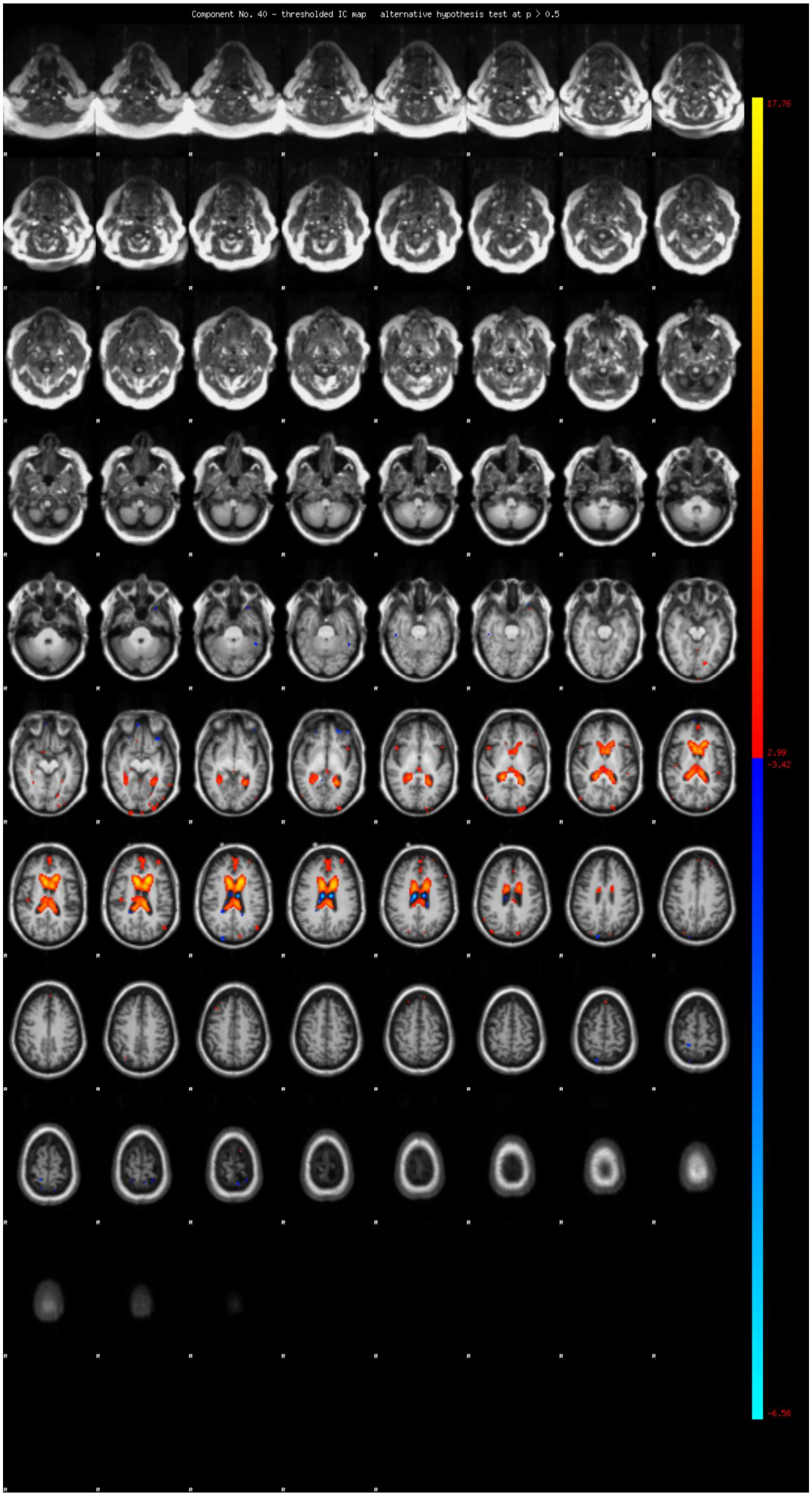

HF

AROMA – noise components for the example subject

(HF = high frequency noise, Motion = high correlation with subject motion, CSF = high correlation with CSF regions)

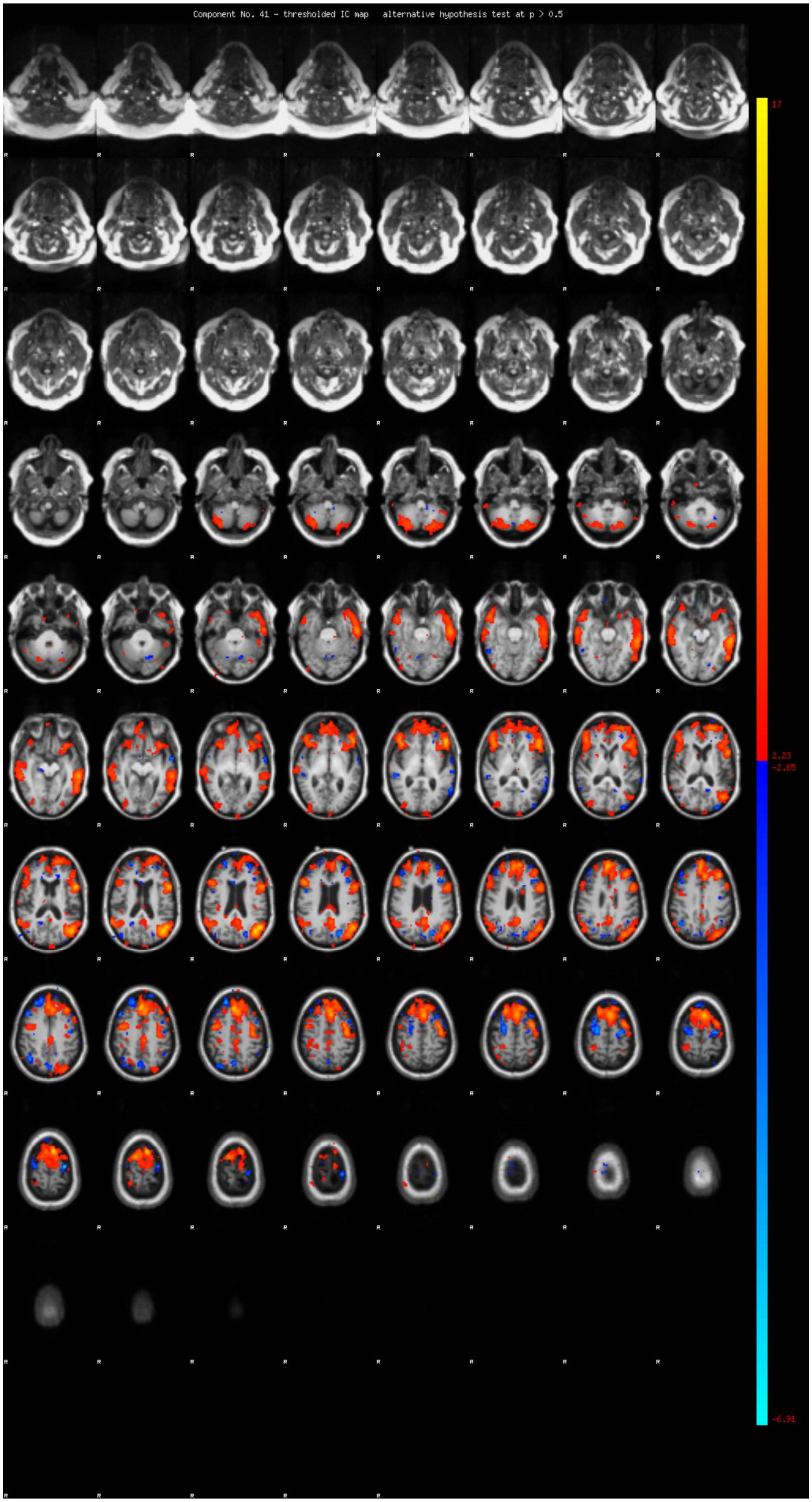

Motion

AROMA – noise components for the example subject

(HF = high frequency noise, Motion = high correlation with subject motion, CSF = high correlation with CSF regions)

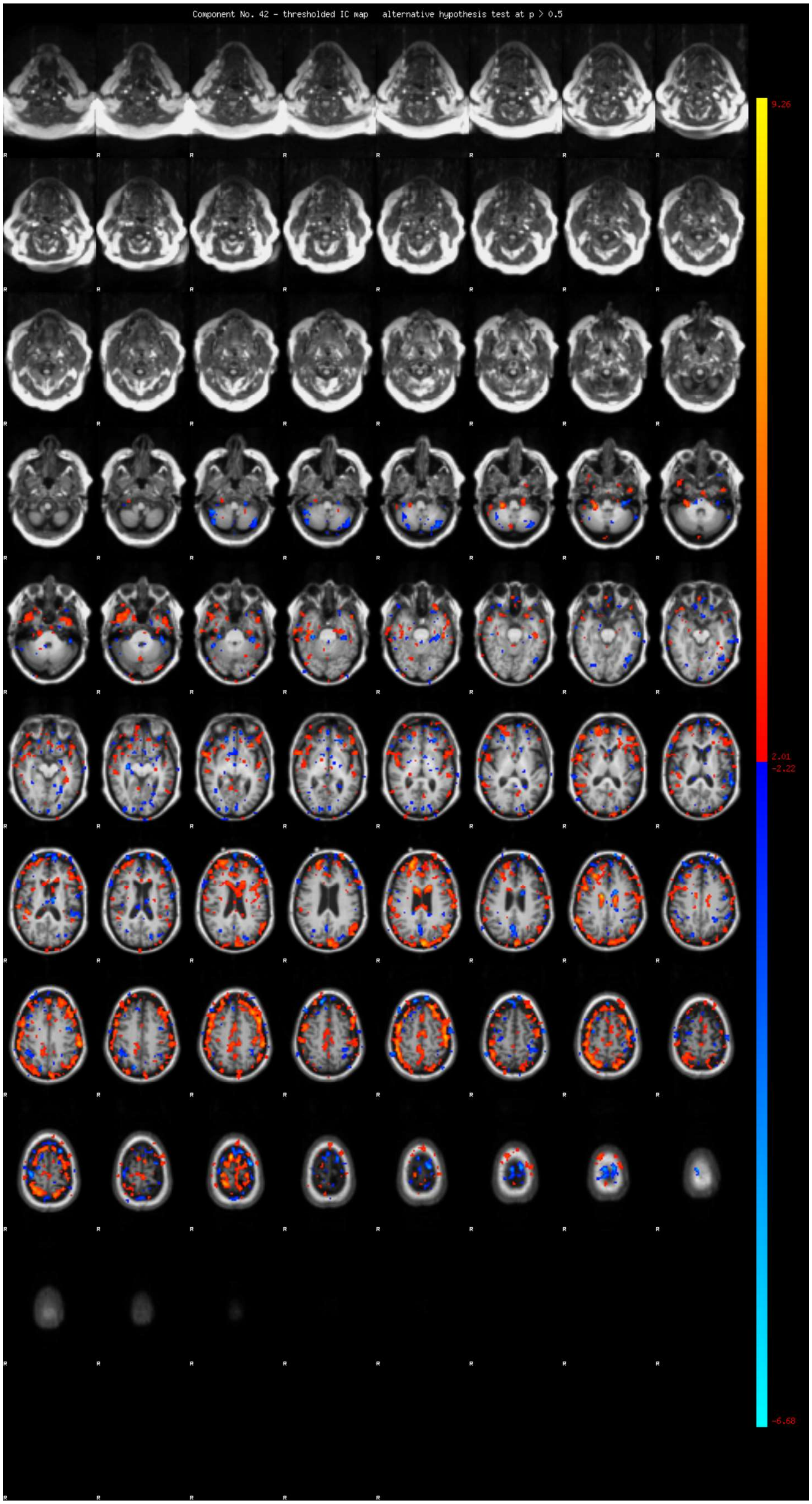

HF/Motion

AROMA – noise components for the example subject

(HF = high frequency noise, Motion = high correlation with subject motion, CSF = high correlation with CSF regions)

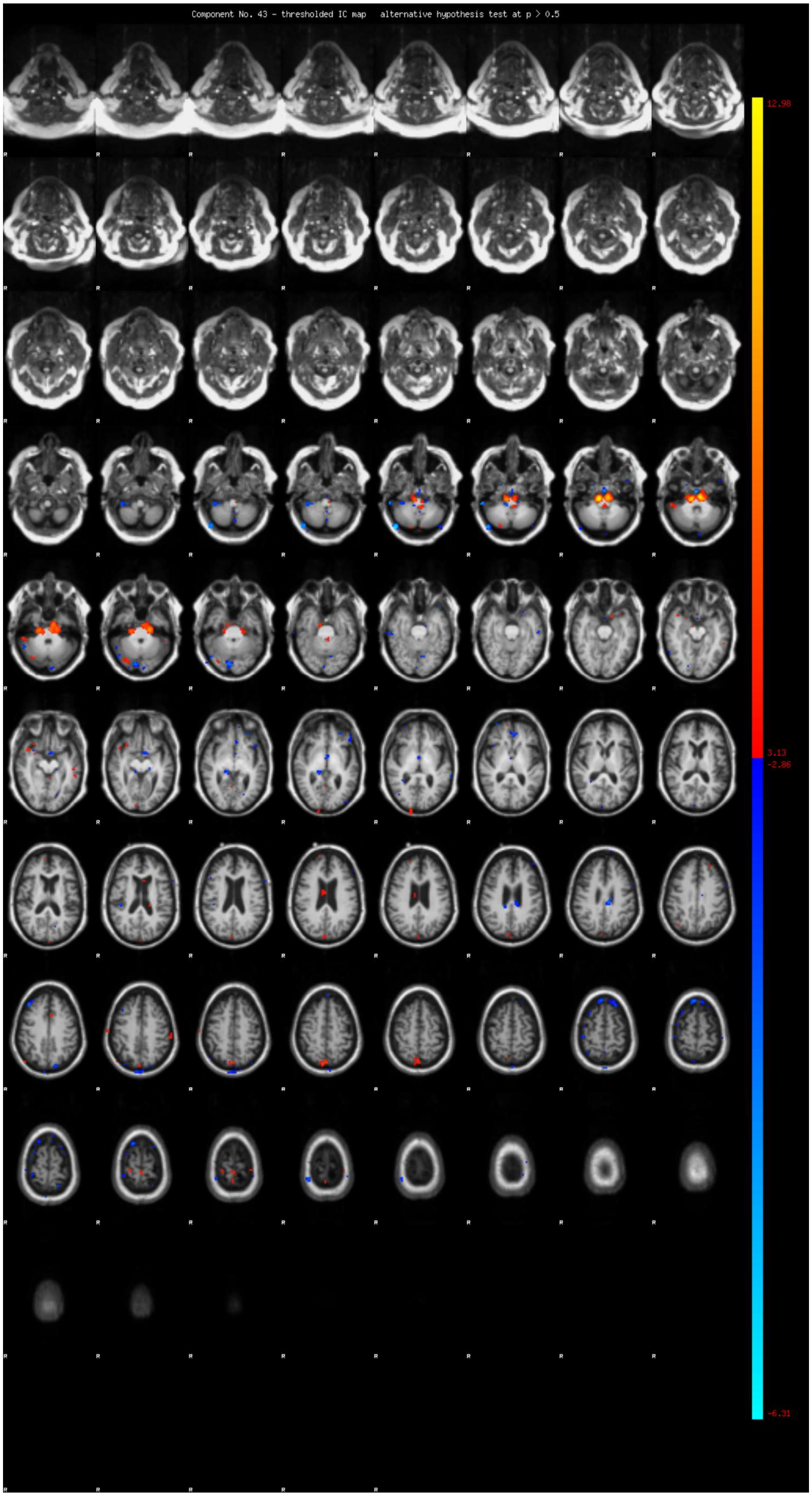

HF

AROMA – noise components for the example subject

(HF = high frequency noise, Motion = high correlation with subject motion, CSF = high correlation with CSF regions)

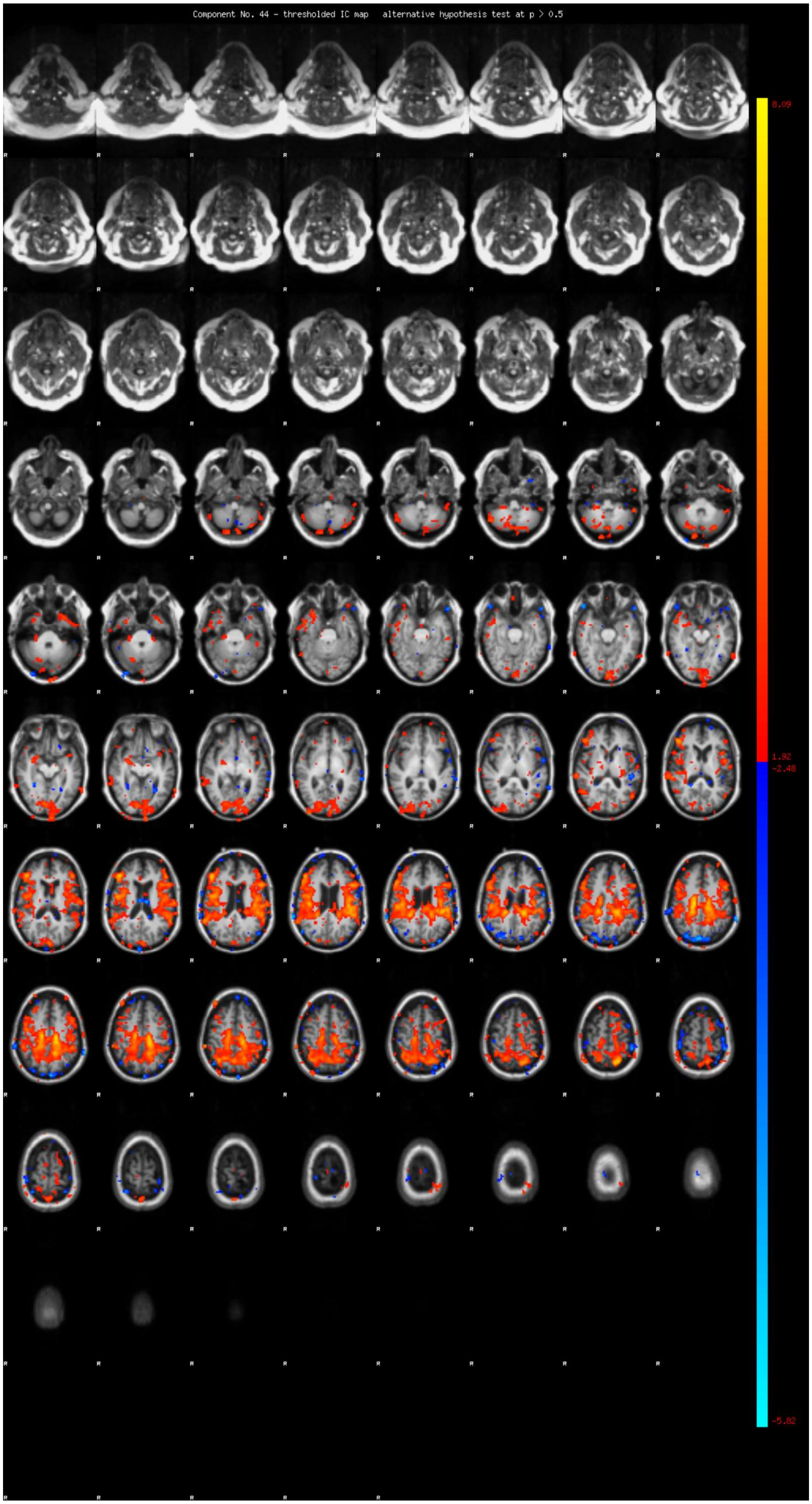

HF

AROMA – noise components for the example subject

(HF = high frequency noise, Motion = high correlation with subject motion, CSF = high correlation with CSF regions)

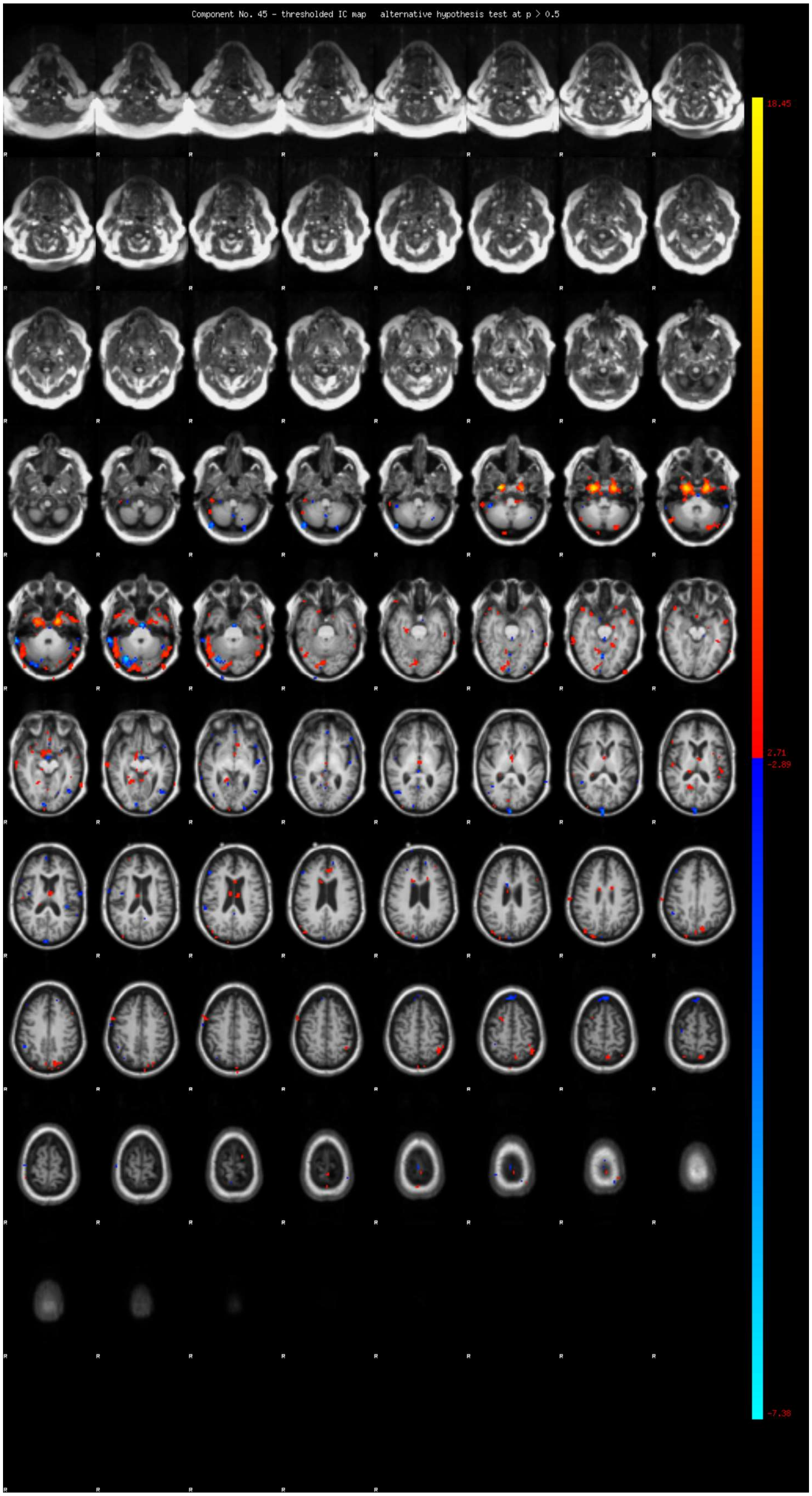

HF

AROMA – noise components for the example subject

(HF = high frequency noise, Motion = high correlation with subject motion, CSF = high correlation with CSF regions)

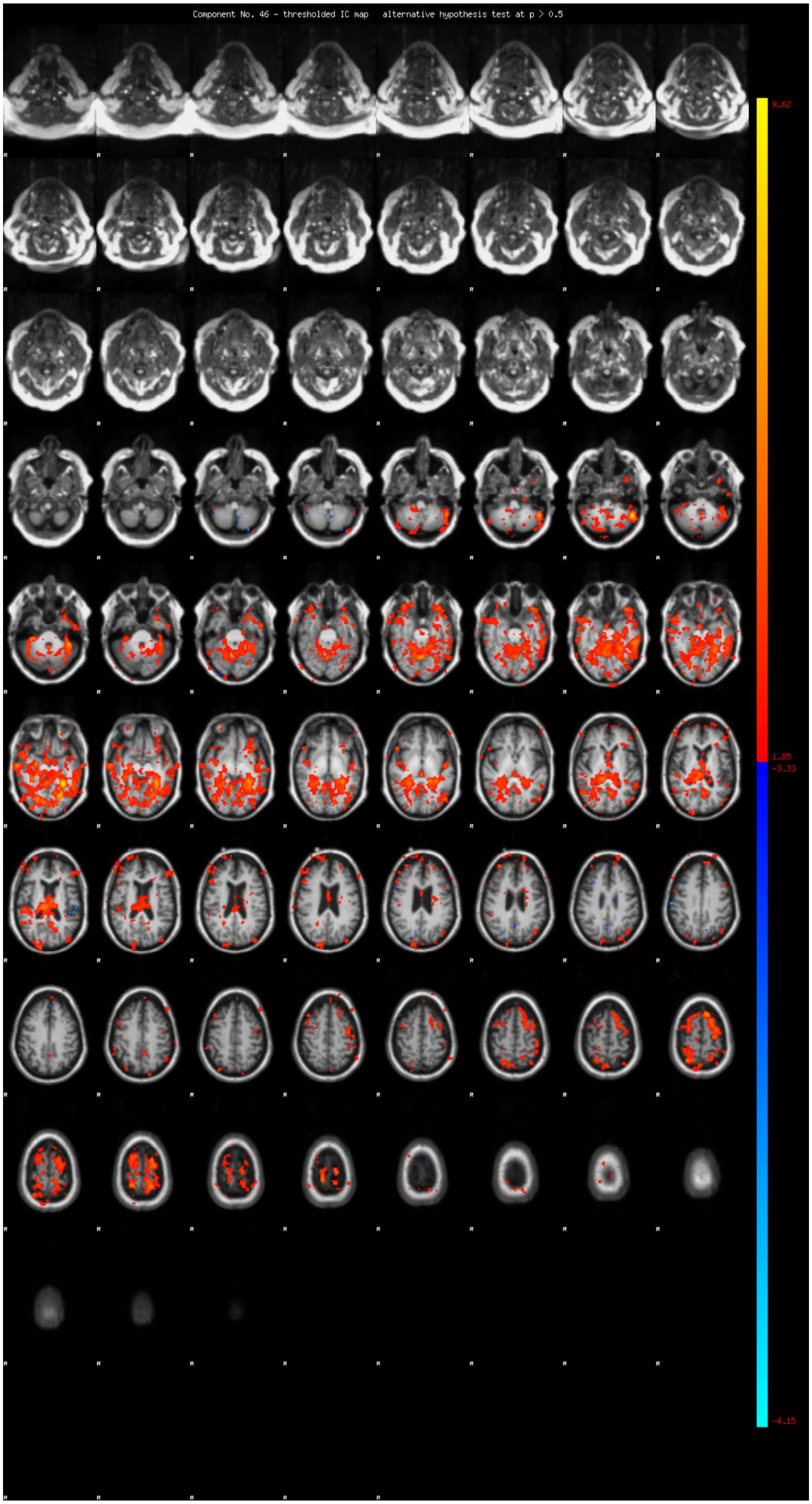

HF

AROMA – noise components for the example subject

(HF = high frequency noise, Motion = high correlation with subject motion, CSF = high correlation with CSF regions)

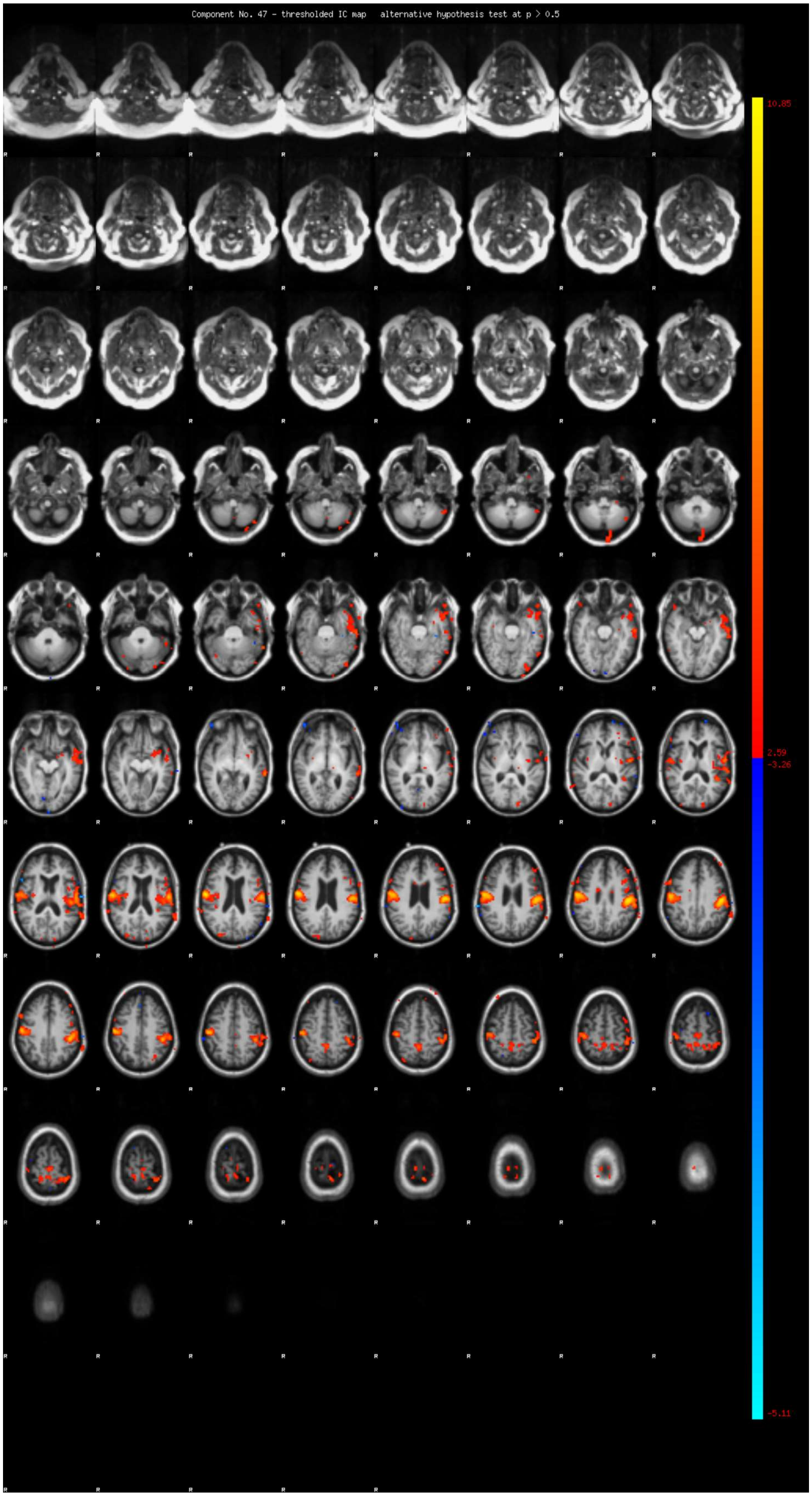

Motion

AROMA – noise components for the example subject

(HF = high frequency noise, Motion = high correlation with subject motion, CSF = high correlation with CSF regions)

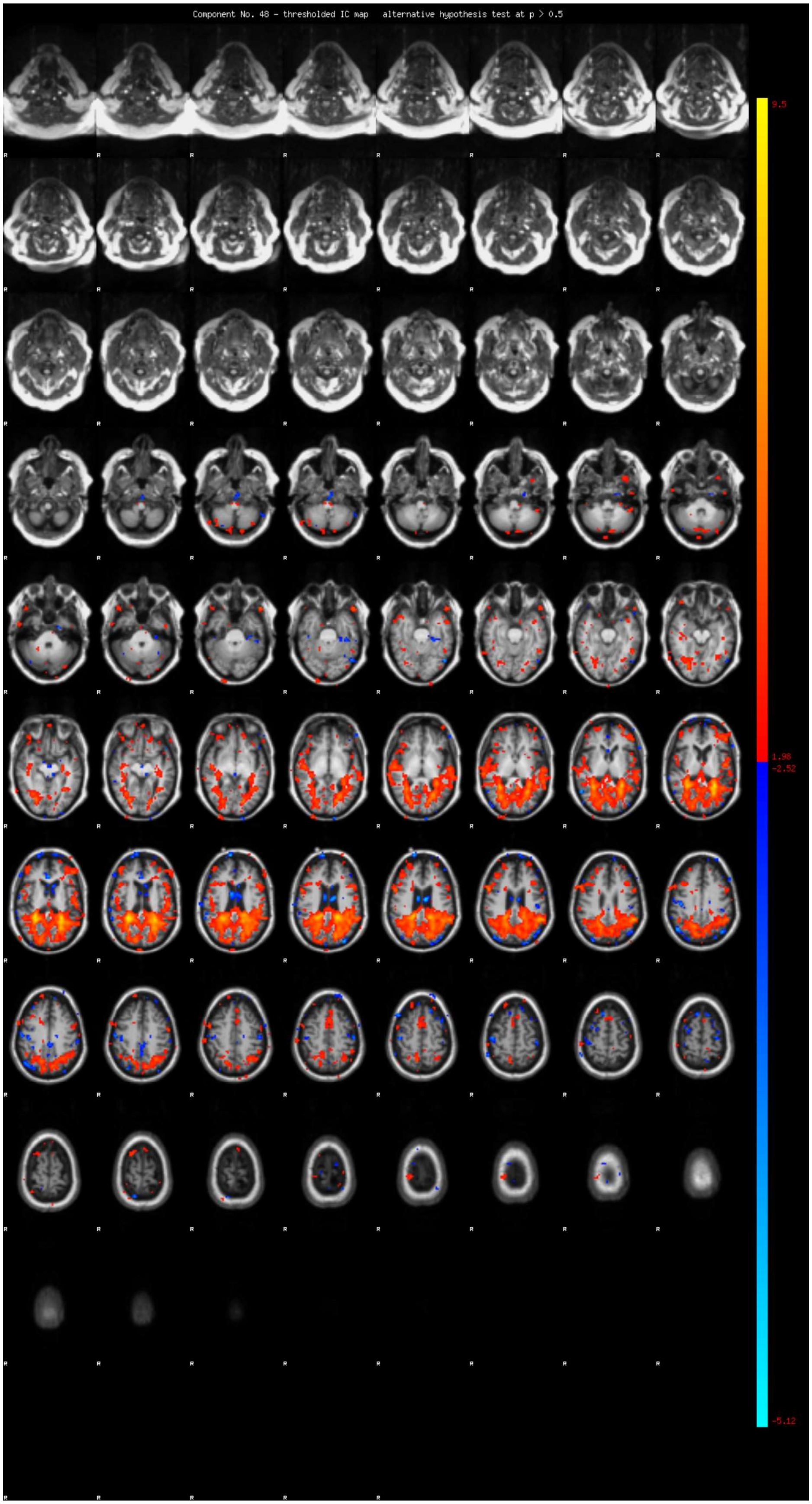

HF

AROMA – noise components for the example subject

(HF = high frequency noise, Motion = high correlation with subject motion, CSF = high correlation with CSF regions)

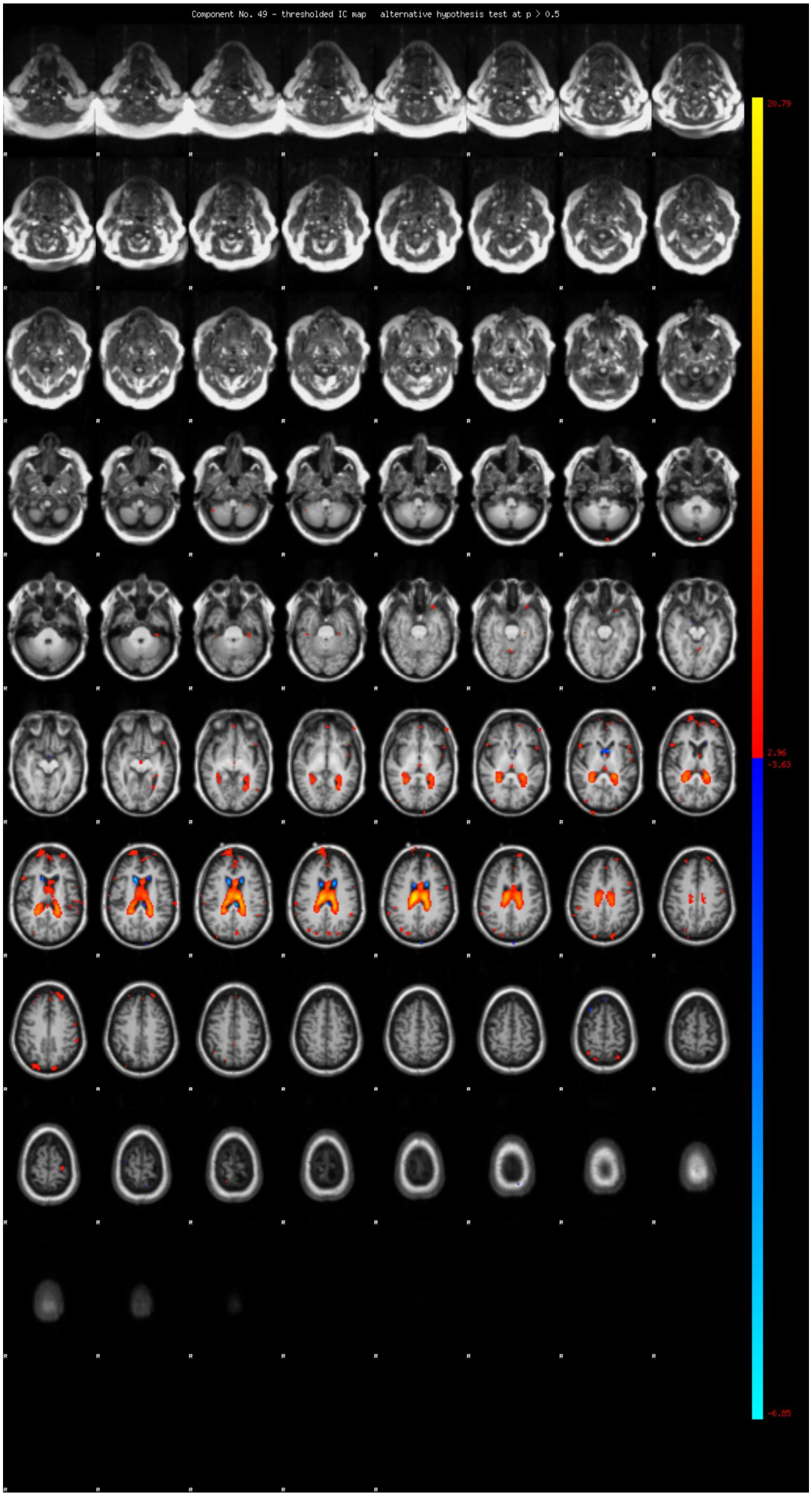

HF

AROMA – noise components for the example subject

(HF = high frequency noise, Motion = high correlation with subject motion, CSF = high correlation with CSF regions)

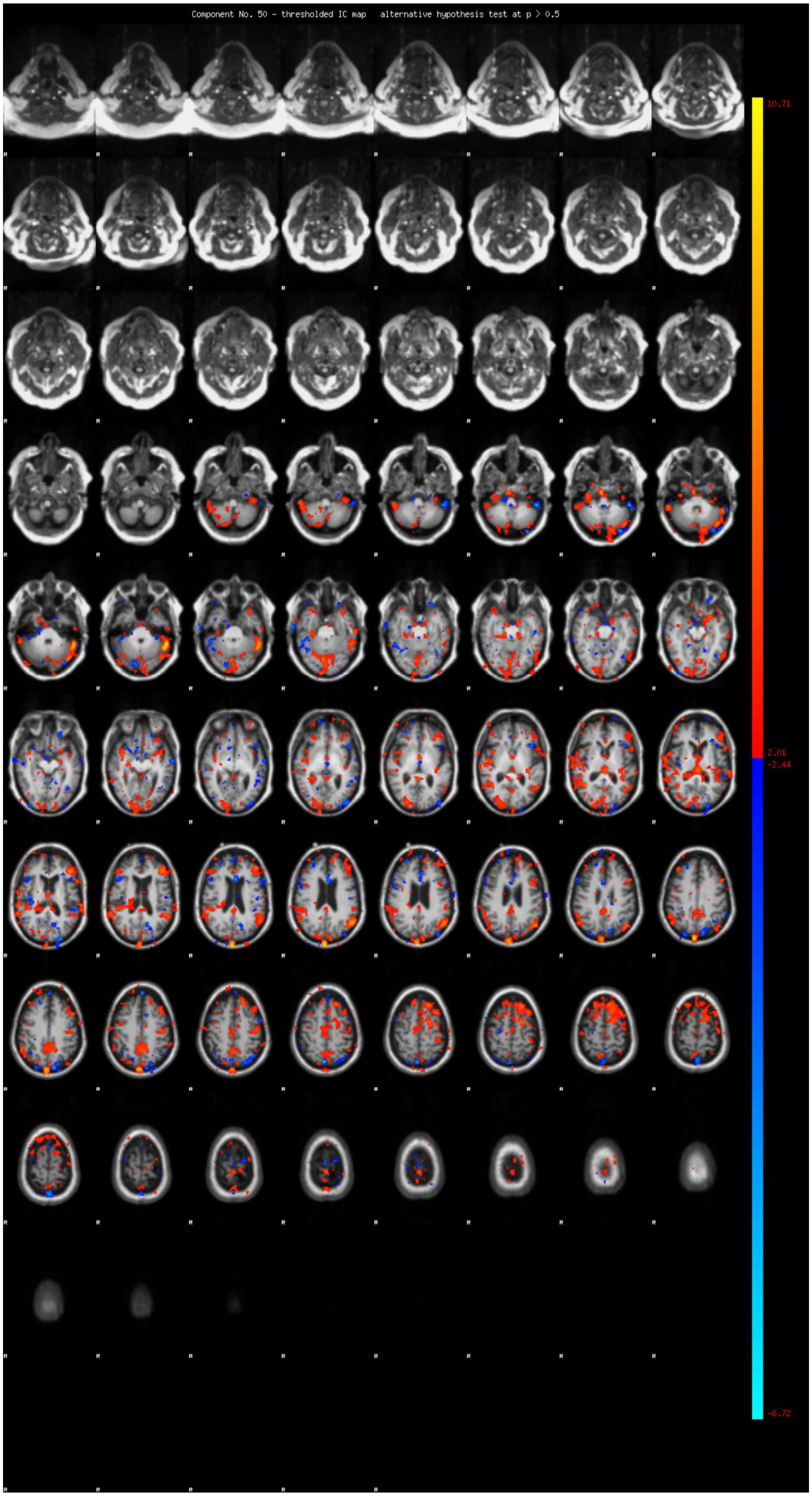

Motion

AROMA – noise components for the example subject

(HF = high frequency noise, Motion = high correlation with subject motion, CSF = high correlation with CSF regions)

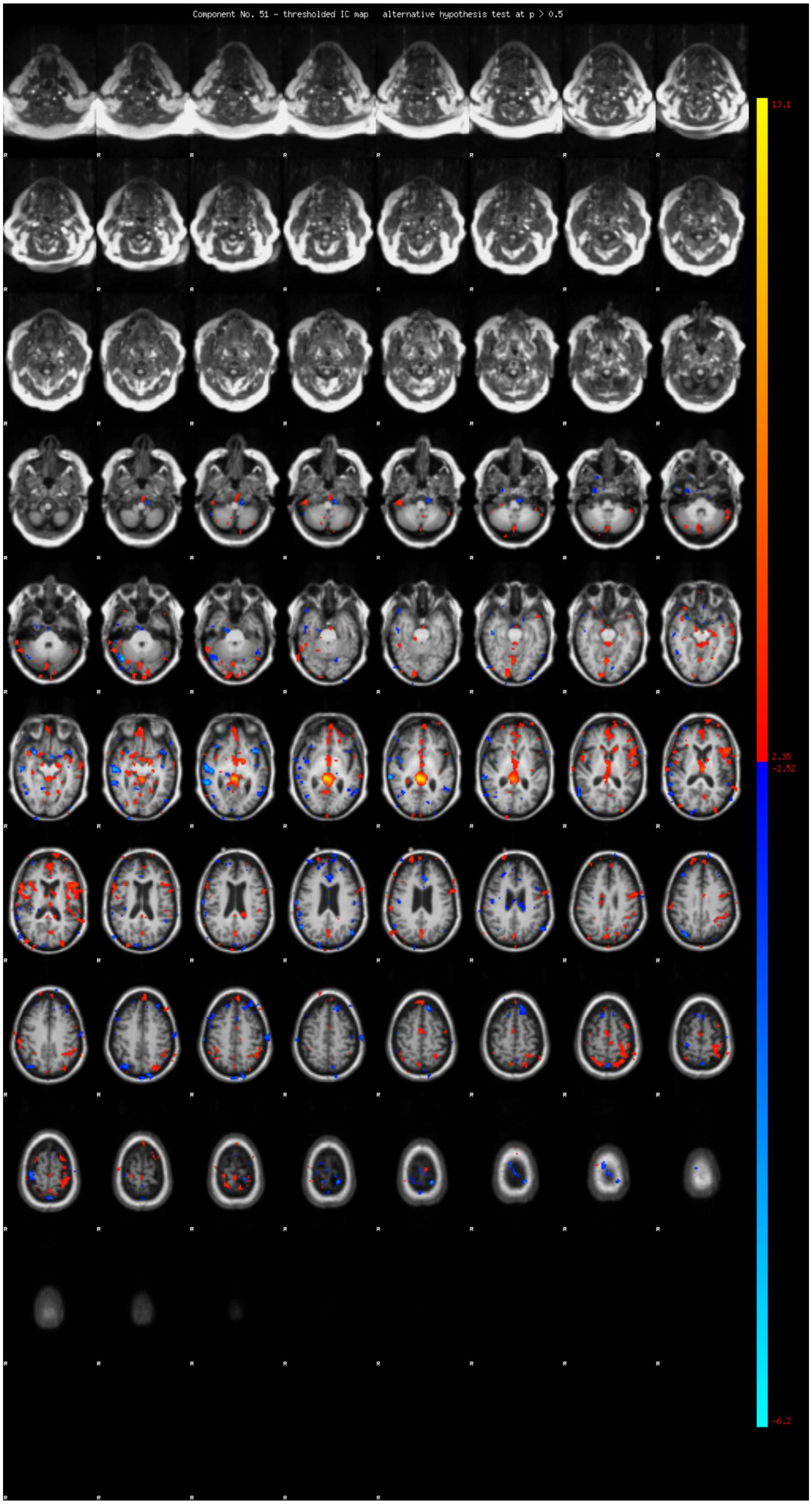

HF

AROMA – noise components for the example subject

(HF = high frequency noise, Motion = high correlation with subject motion, CSF = high correlation with CSF regions)

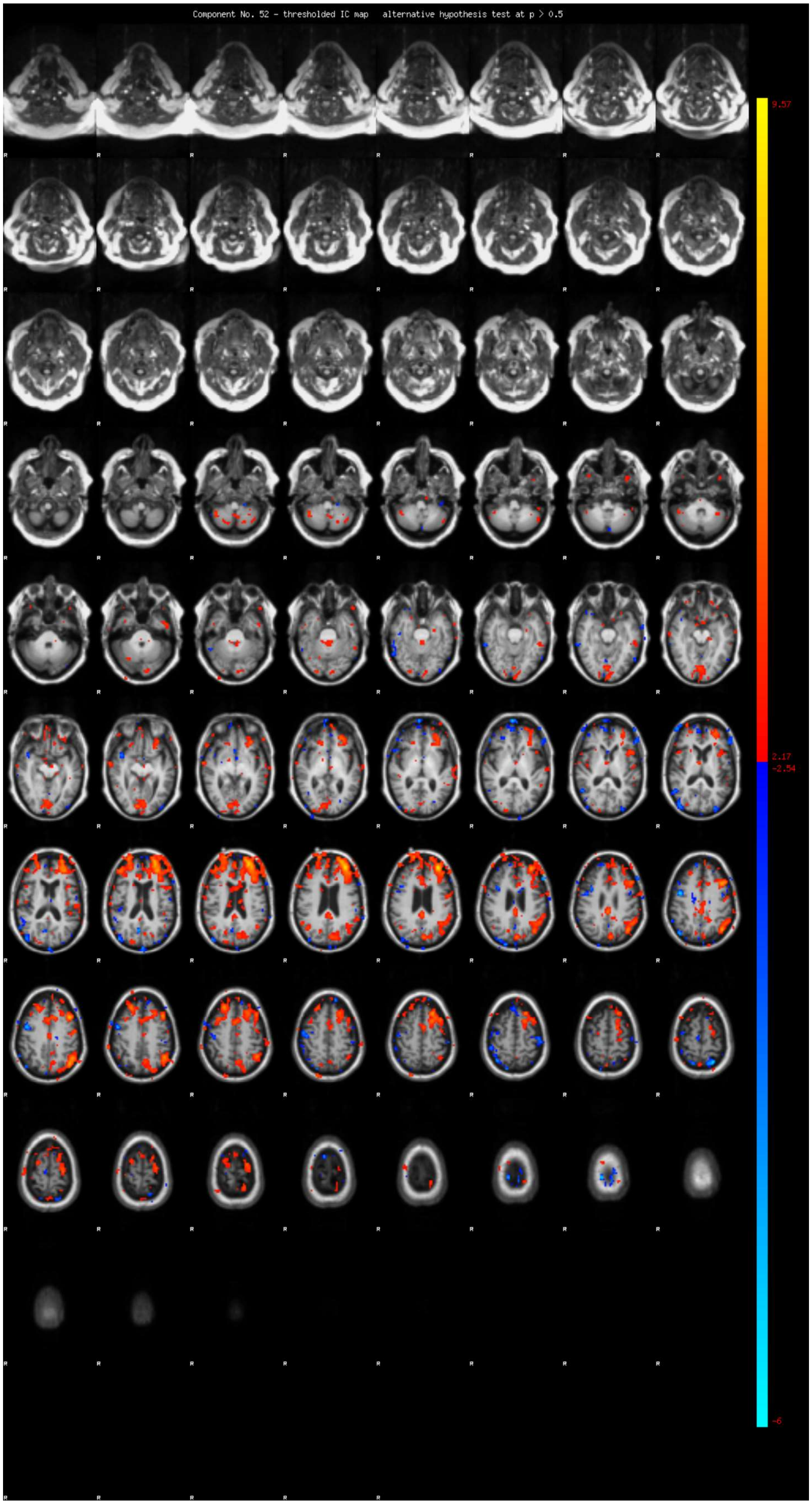

HF

AROMA – noise components for the example subject

(HF = high frequency noise, Motion = high correlation with subject motion, CSF = high correlation with CSF regions)

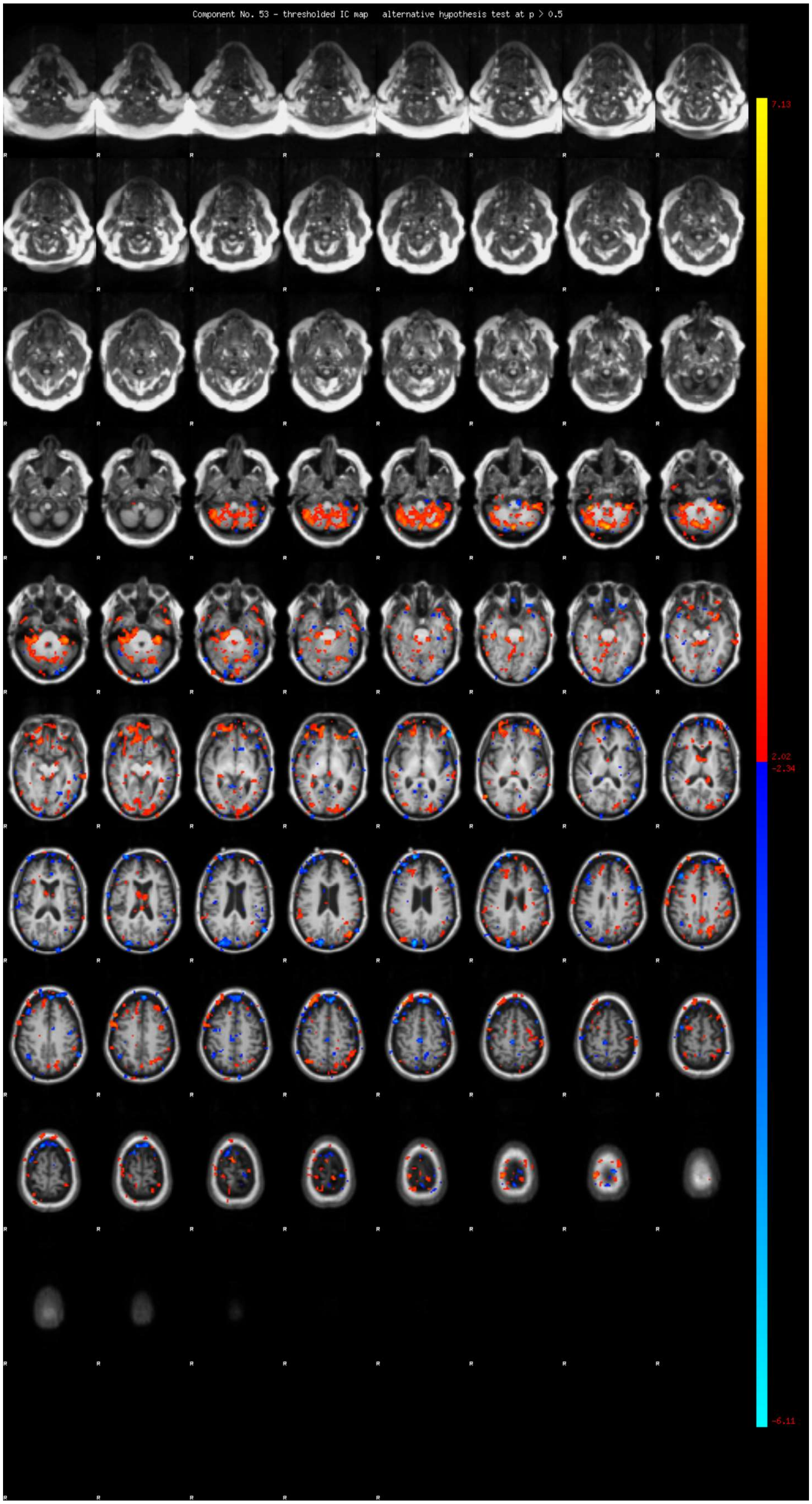

HF/Motion

AROMA – noise components for the example subject

(HF = high frequency noise, Motion = high correlation with subject motion, CSF = high correlation with CSF regions)

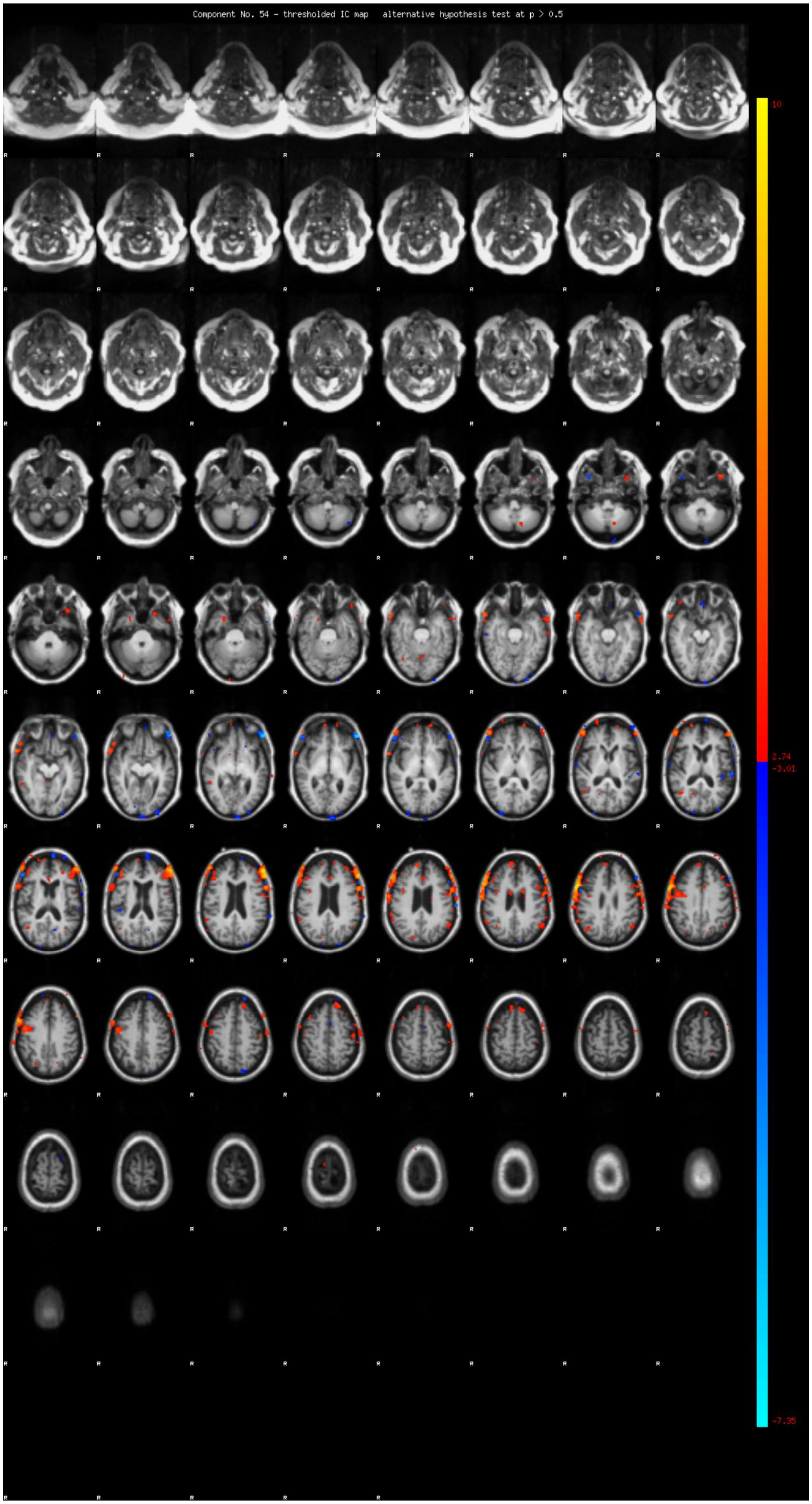

Motion

AROMA – noise components for the example subject

(HF = high frequency noise, Motion = high correlation with subject motion, CSF = high correlation with CSF regions)

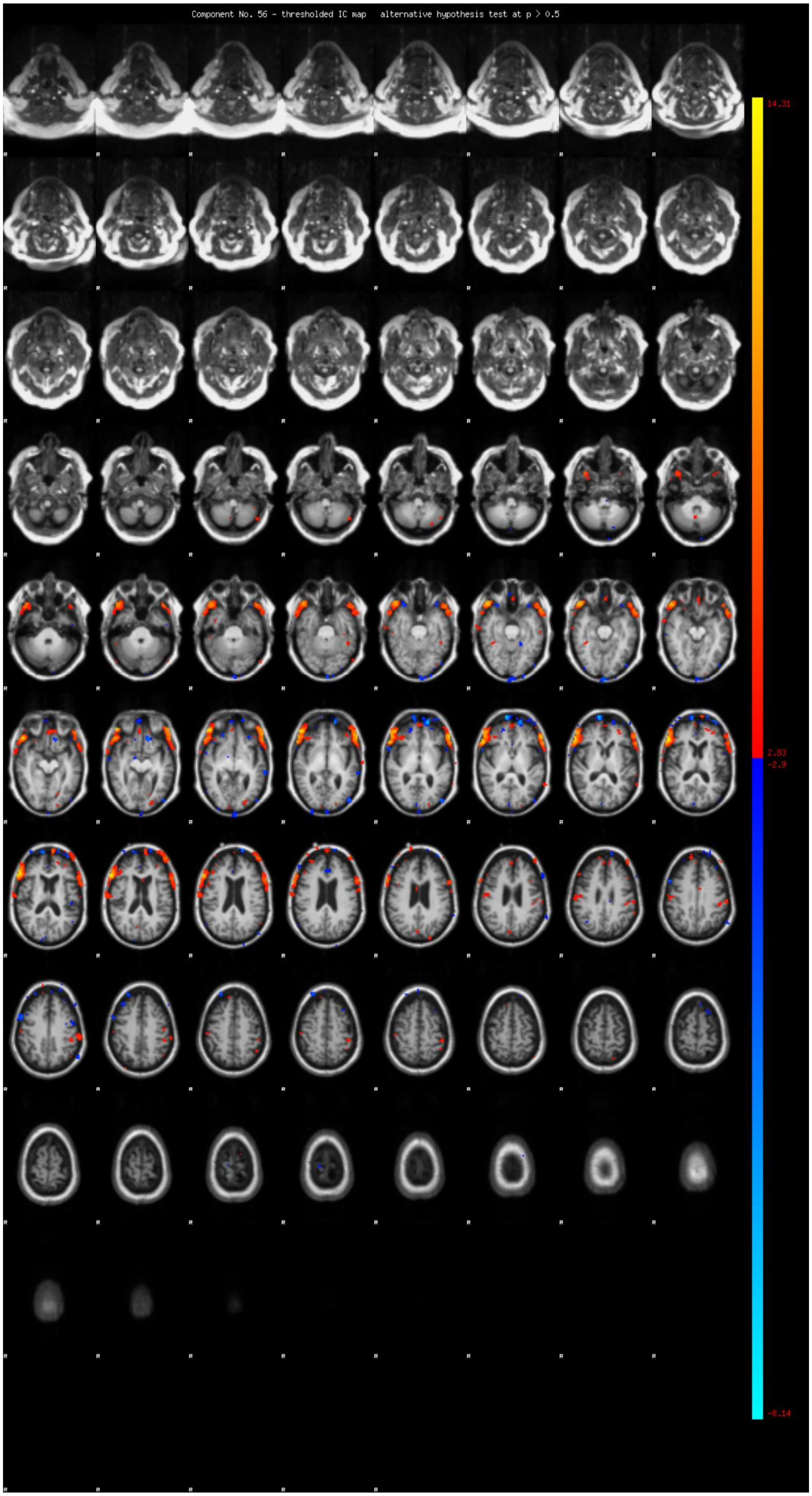

Motion

AROMA – noise components for the example subject

(HF = high frequency noise, Motion = high correlation with subject motion, CSF = high correlation with CSF regions)

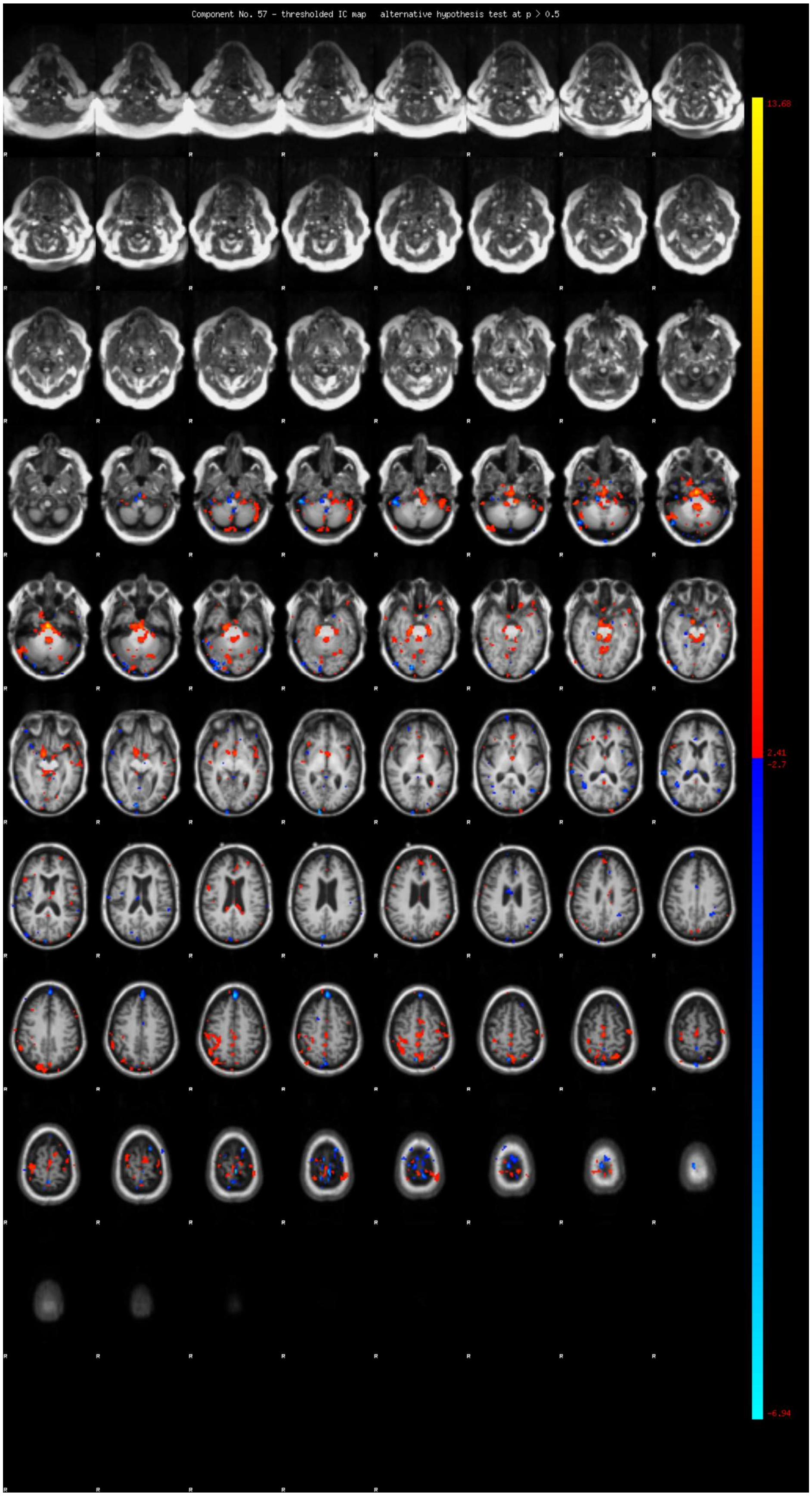

HF

AROMA – non-noise components for the example subject

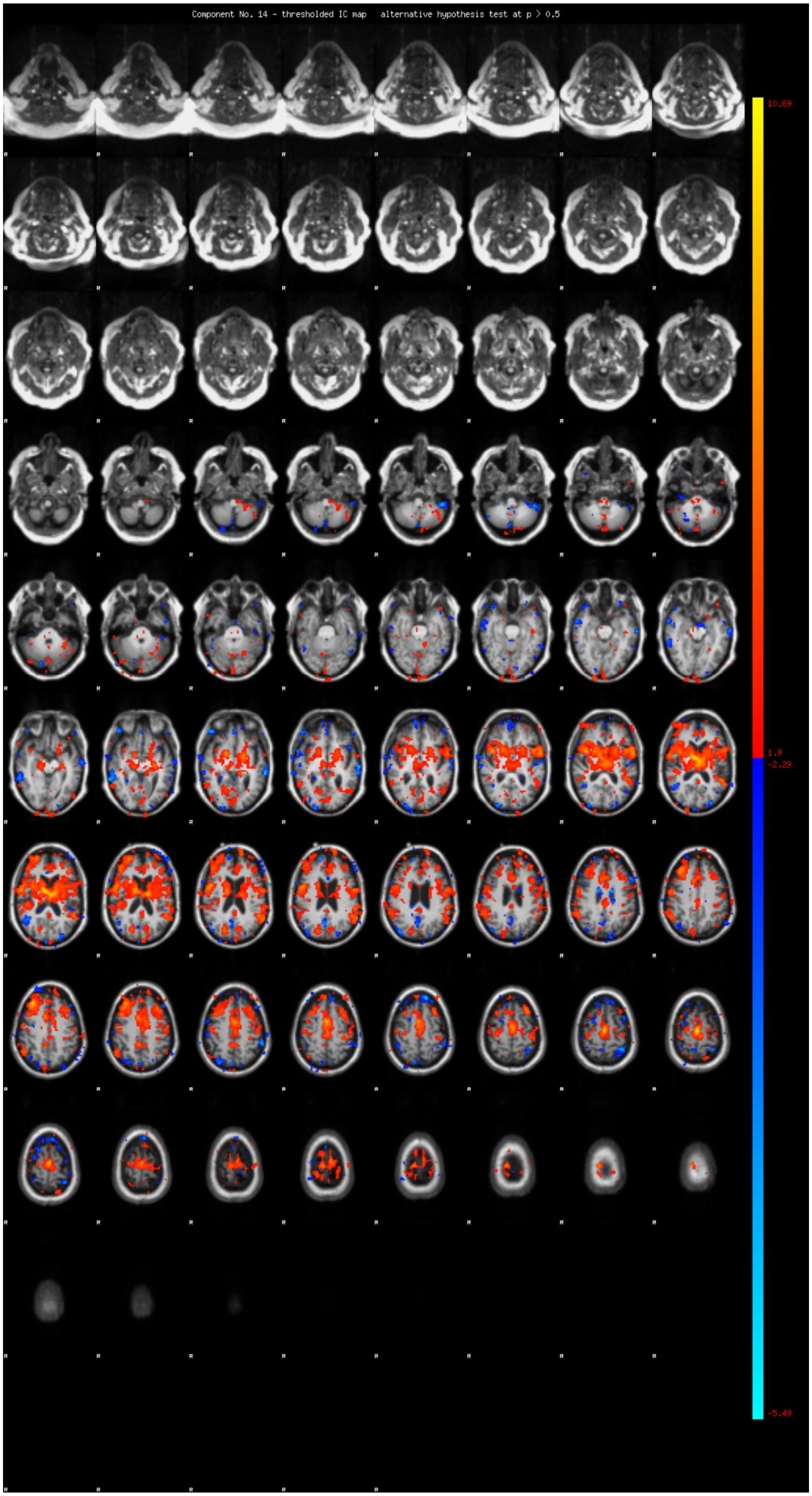

AROMA – non-noise components for the example subject

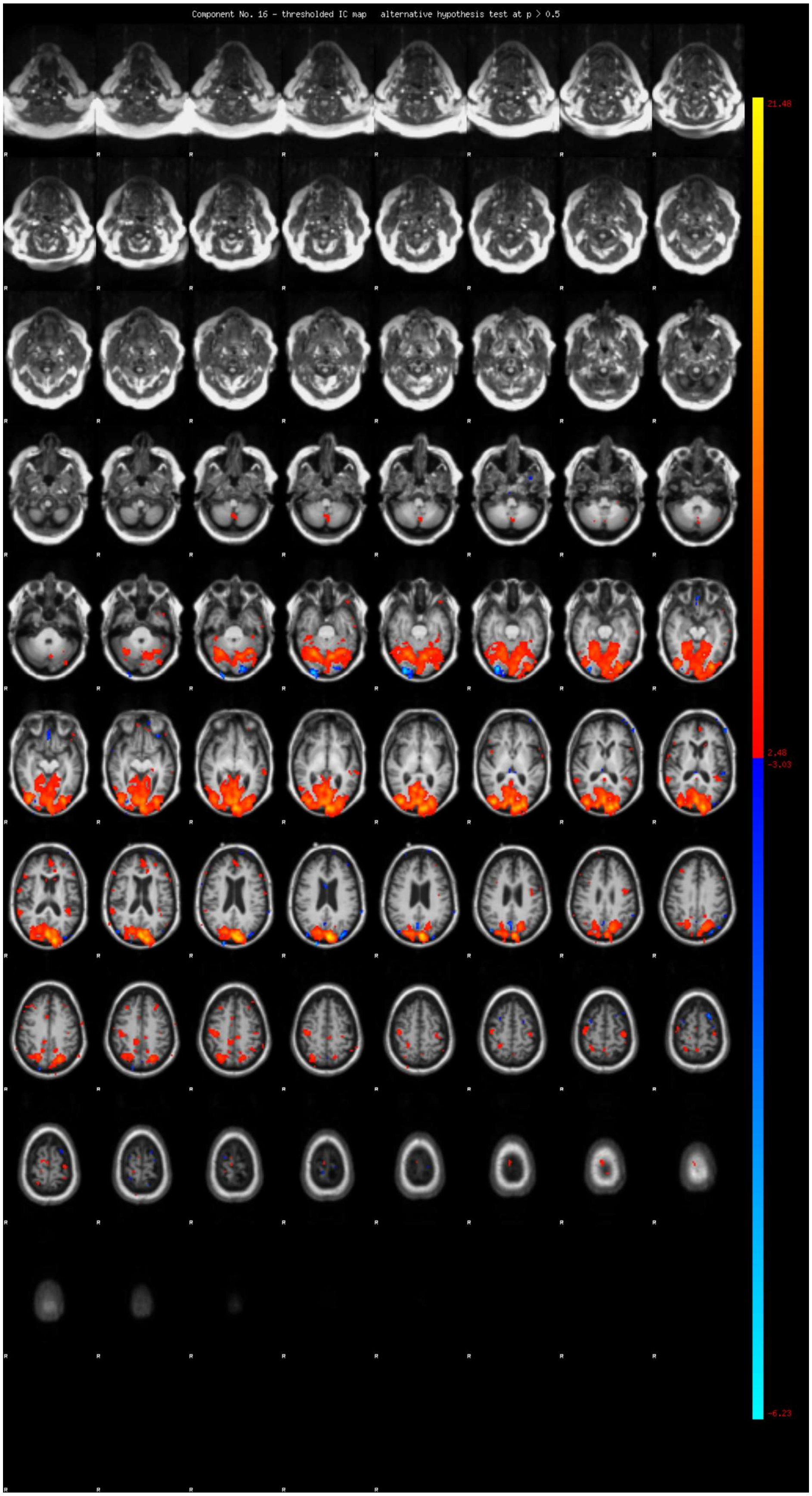

AROMA – non-noise components for the example subject

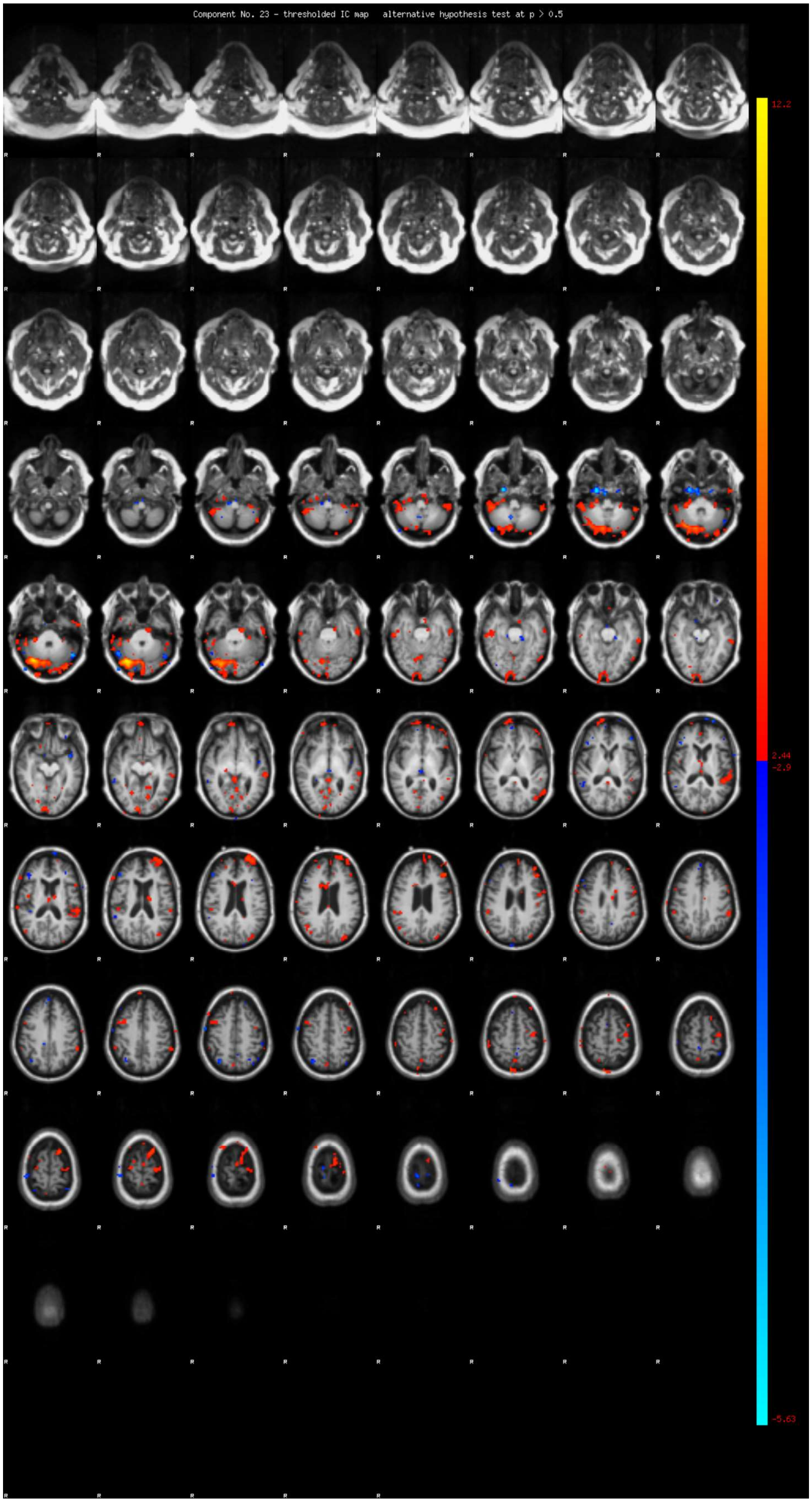

AROMA – non-noise components for the example subject

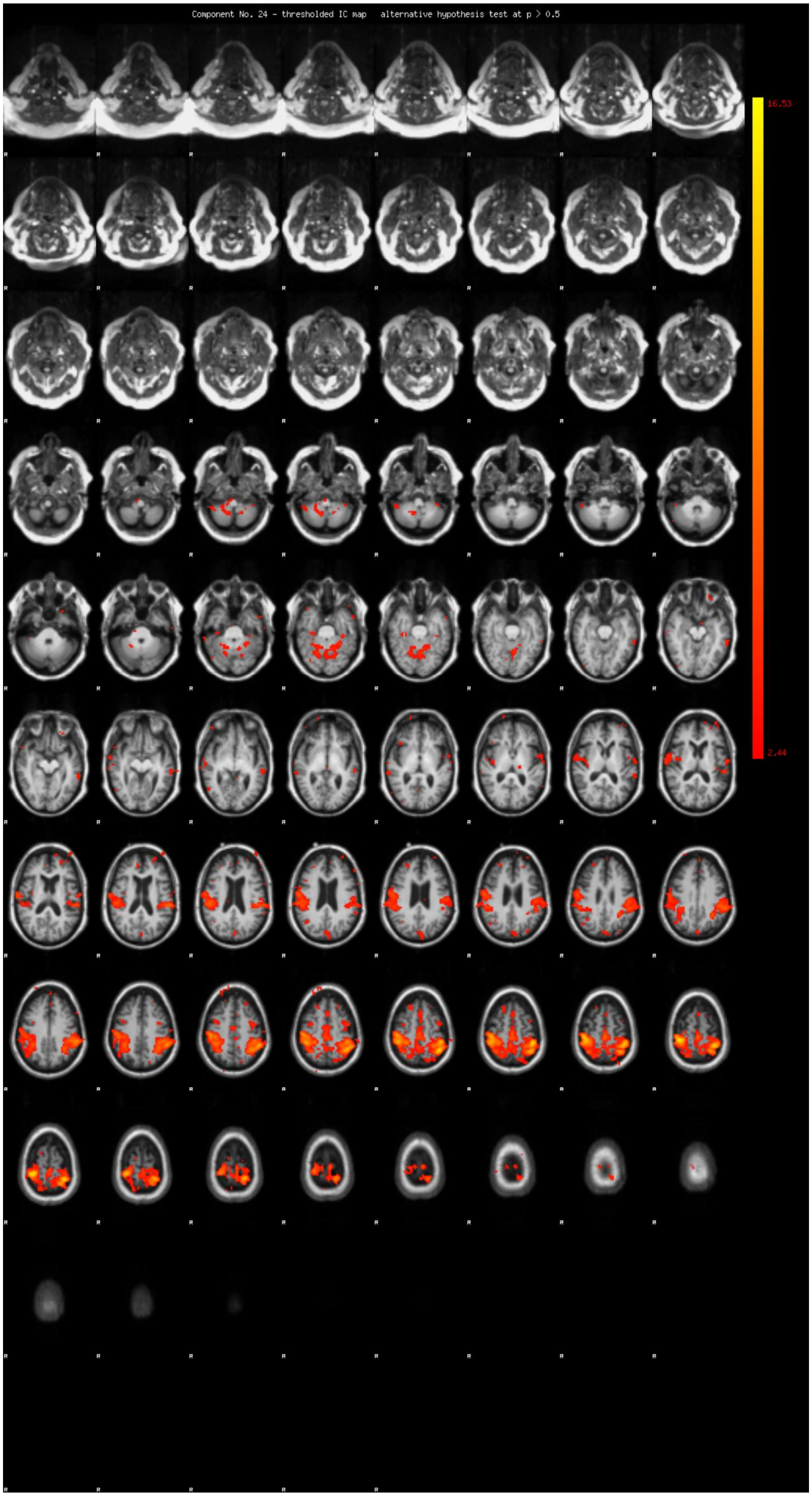

AROMA – non-noise components for the example subject

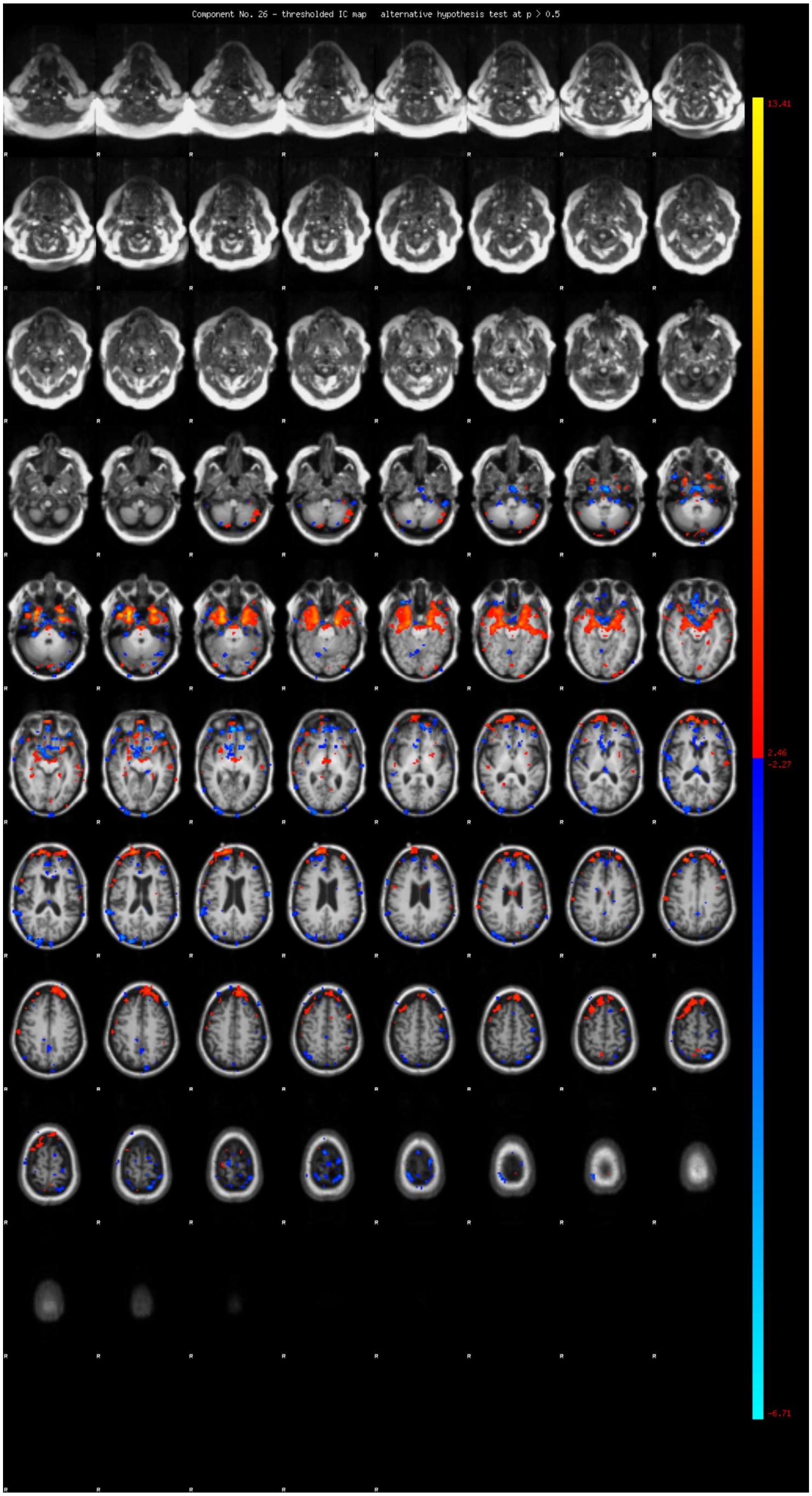

AROMA – non-noise components for the example subject

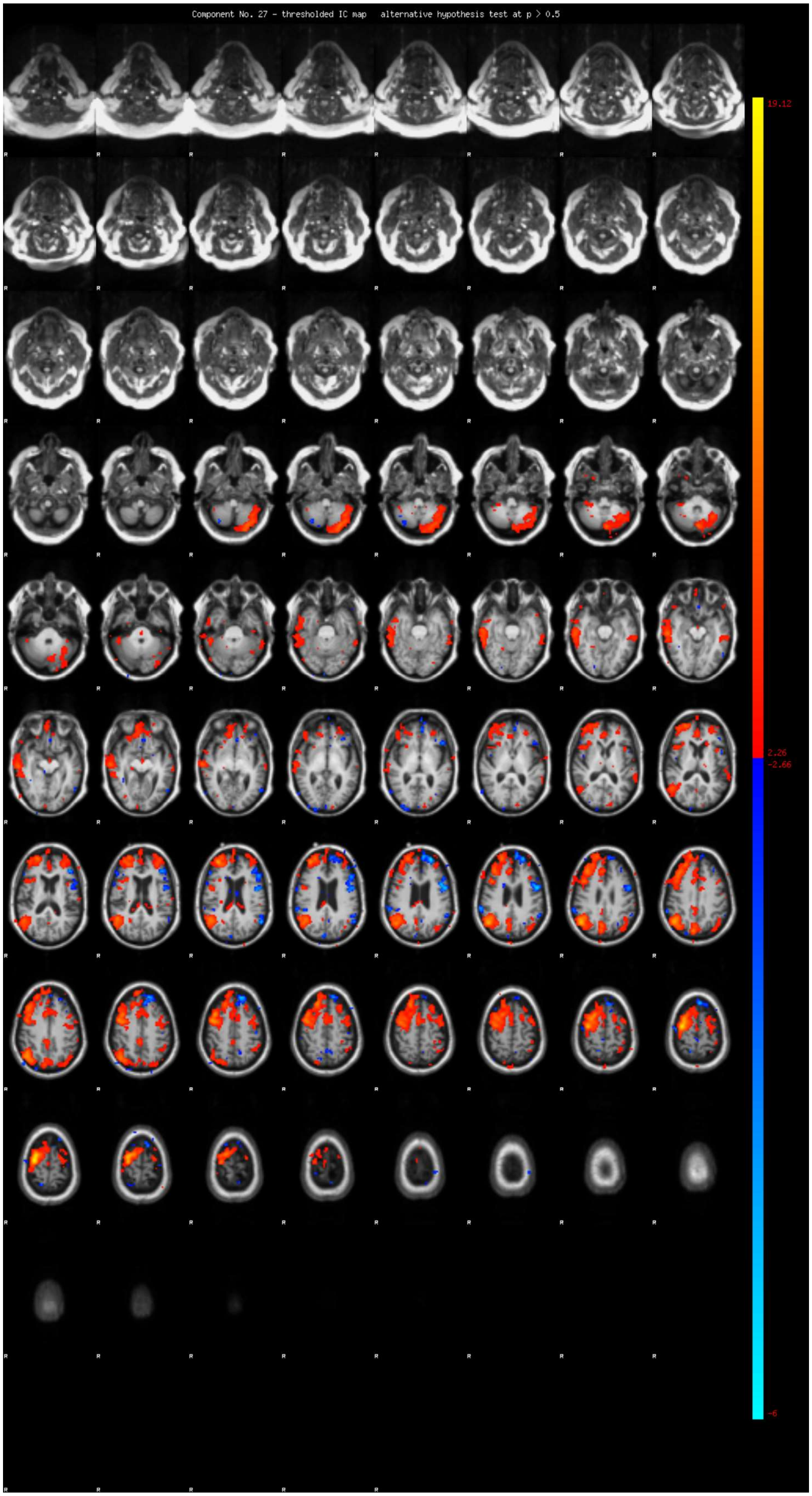

AROMA – non-noise components for the example subject

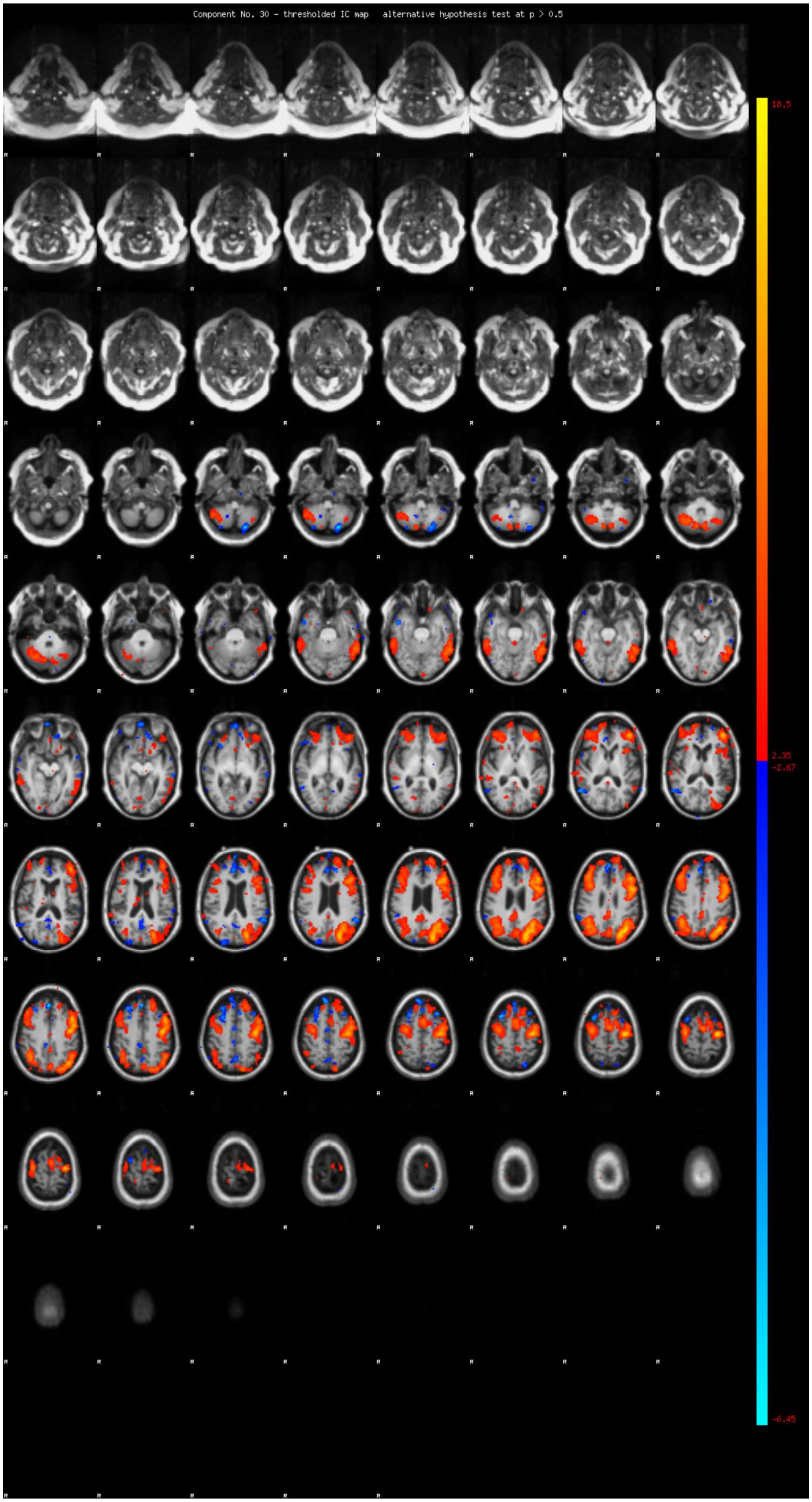

AROMA – non-noise components for the example subject

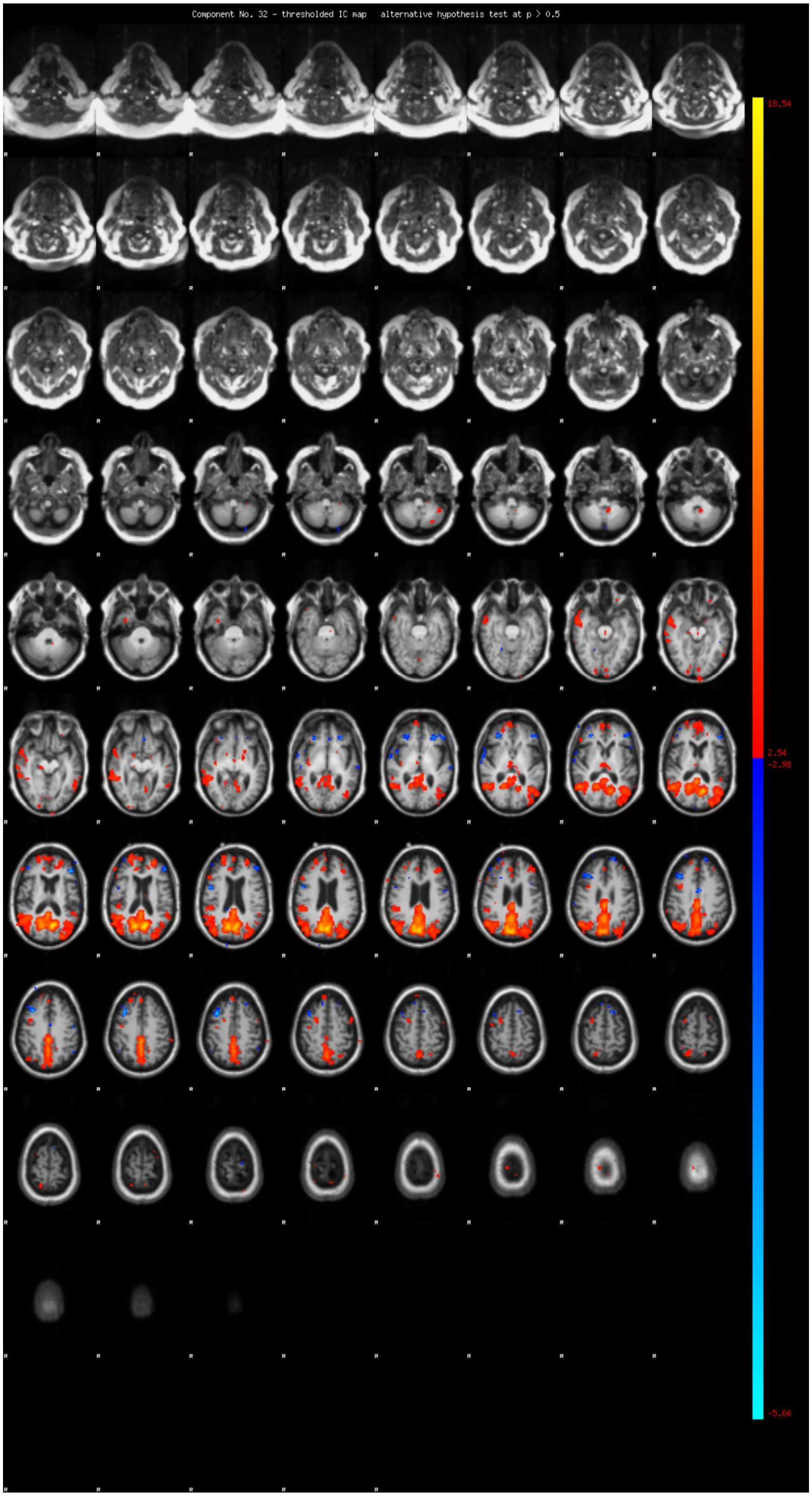

AROMA – non-noise components for the example subject

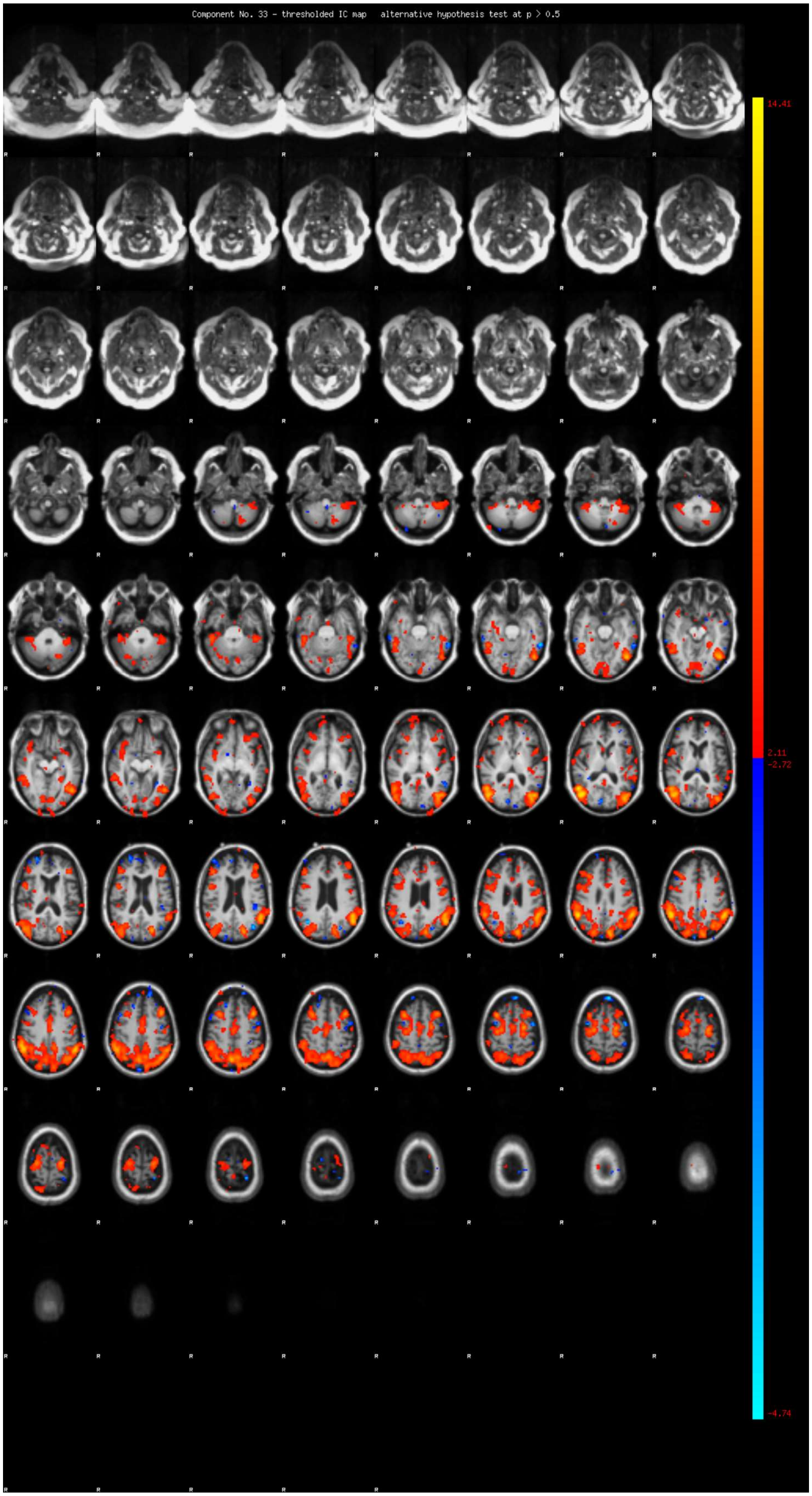

AROMA – non-noise components for the example subject

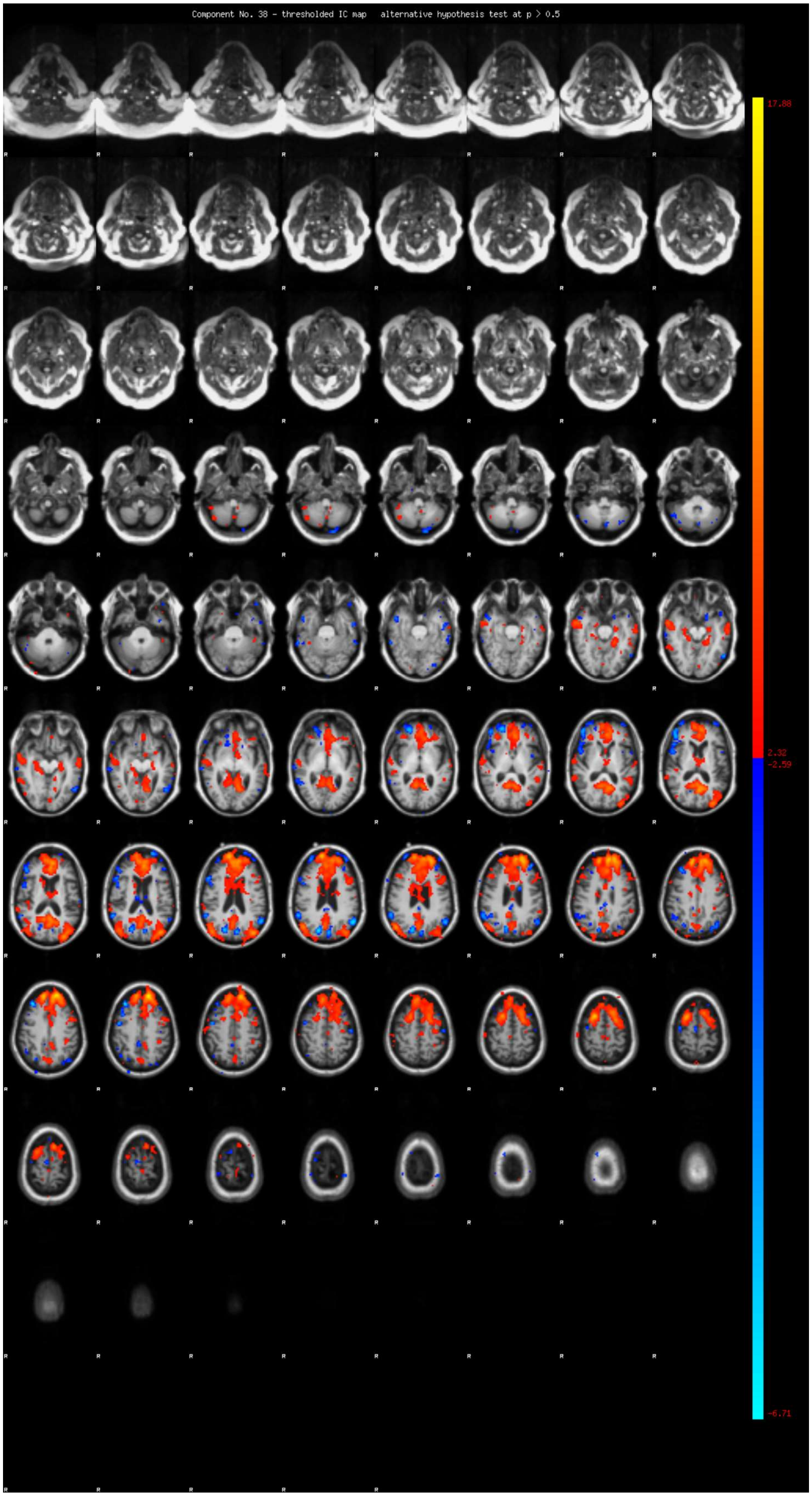

AROMA – non-noise components for the example subject

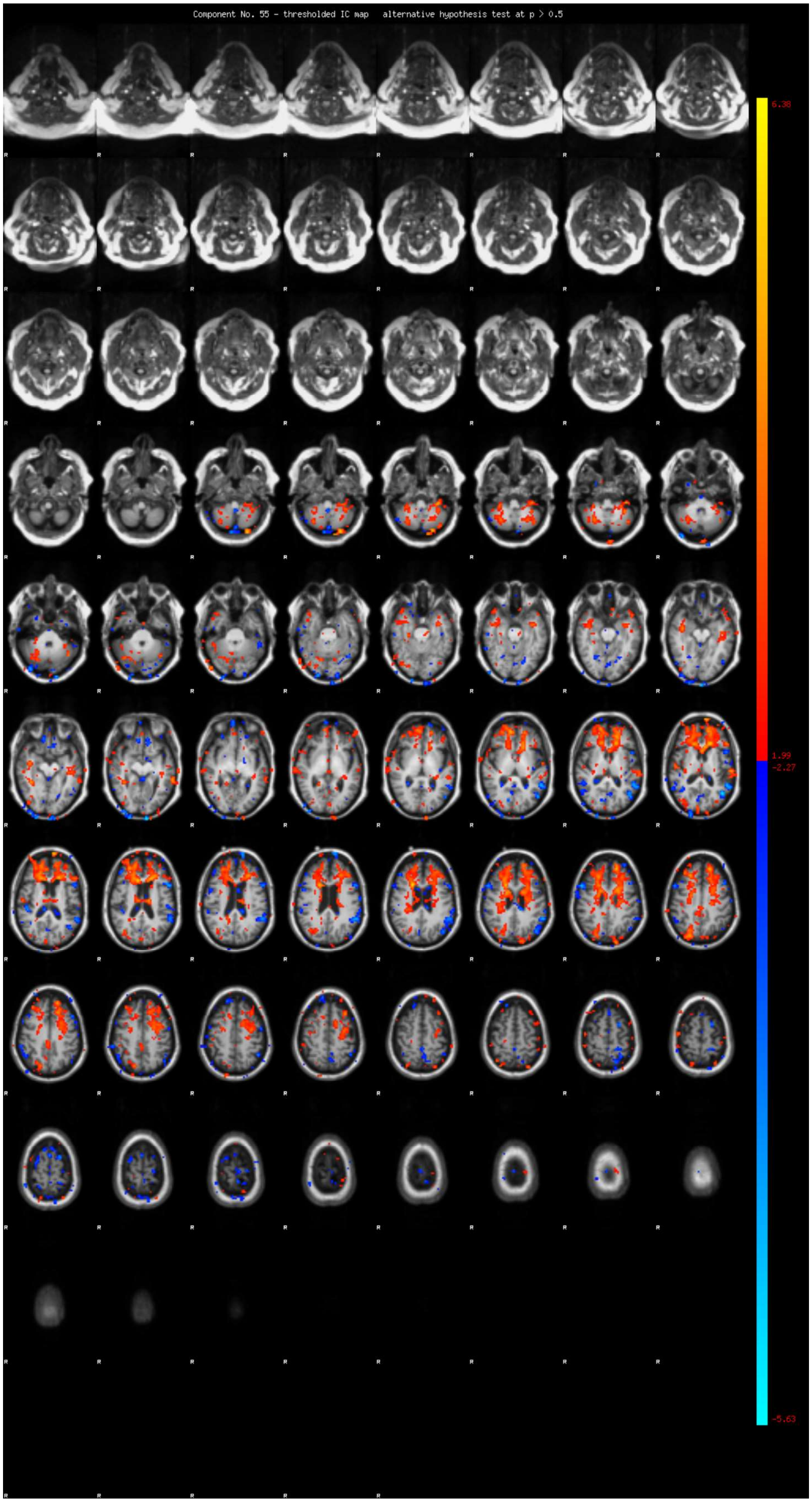

Supplement: Supplementary file 1 [file Data_Sheet_1.pdf]
